# The prevalence of prolonged grief disorder according to the international classification of diseases 11: a scoping review

**DOI:** 10.1007/s00406-025-02046-4

**Published:** 2025-06-25

**Authors:** Kiana Nafarieh, Sophia Krüger, Karl Deutscher, Stefanie Schreiter, Adrian P. Mundt, Andreas Jung, Seena Fazel, Andreas Heinz, Stefan Gutwinski

**Affiliations:** 1 Department of Psychiatry and Psychotherapy, Charité– Universitätsmedizin Berlin, Corporate Member of Freie Universität Berlin, Humboldt-Universität zu Berlin, Berlin Institute of Health, Charité– Universitätsmedizin Berlin, 10117 Berlin, Germany; 2 Medical Faculty, Universidad Diego Portales, Santiago, Chile; 3 EX-IN Hessen e.V, Marburg, Germany; 4 Department of Psychiatry, University of Oxford, Oxford, UK; 5 Department of Psychiatry and Psychotherapy, Tübingen Center for Mental Health (TüCMH), Medical School and University Hospital, Eberhard Karls University of Tübingen, Tübingen, Germany; 6 German Center for Mental Health (DZPG), Partner Site Tübingen, Tübingen, Germany

**Keywords:** Prolonged grief disorder, Complicated grief, ICD-11, Epidemiology, Psychiatry

## Abstract

**Background:**

The eleventh revision of the International Classification of Diseases (ICD-11) introduces Prolonged Grief Disorder (PGD) as a new diagnostic category. This paper summarizes methodological approaches and prevalence estimates of studies on PGD in ICD-11.

**Methods:**

This review follows the JBI Manual of Evidence Synthesis and PRISMA-ScR guidelines. We searched MEDLINE, Embase, Web of Science, and PsycINFO (2011–2024), along with grey literature sources (Web of Science, Science.gov, NDLTD Global ETD Search). Included studies were cross-sectional or longitudinal, evaluating PGD prevalence using ICD-11 criteria. Two reviewers (KN, SK) independently screened studies, with a third (SG) resolving disagreements. Methodological quality was not assessed. Data extraction covered bibliographic details, study period, location, sample characteristics, diagnostic tools, algorithms, and prevalence.

**Results:**

Of 124 screened records, 35 studies were included in a qualitative synthesis. Seven main study categories emerged, primarily bereaved adults and representative national samples. Of 46 study samples, 24 were from Europe, followed by North America (*n* = 10) and Asia (*n* = 5), with none from South America. The PG-13 was the most commonly used tool, often omitting and raising ICD-11 PGD criteria simultaneously. ICD-11 PGD prevalence ranged from 1.5 to 15.3% in bereaved adults and 9.9–11.4% in national samples.

**Conclusions:**

Findings reveal heterogeneous study populations but limited geographic diversity. Standardized PGD assessments aligned with ICD-11 criteria, using tools specifically designed for ICD-11, along with detailed sample reporting, are needed to improve study comparability and consistency of prevalence. Important gaps by geographical and demographic groups remain.

**Supplementary Information:**

The online version contains supplementary material available at 10.1007/s00406-025-02046-4.

## Introduction

The eleventh revision of the International Statistical Classification of Diseases (ICD-11) was announced by the World Health Organization (WHO) in June 2018 and approved by the 72nd World Health Assembly in May 2019, following arguably the most extensive review process in its history [1, 2]. Key milestones in this revision included the release of the ICD-11 alpha browser in May 2011 and the beta draft in 2015 [3, 4].

Chapter six of ICD-11, which focuses on “Mental, behavioural or neurodevelopmental disorders,” introduces significant changes from ICD-10, including revised diagnostic criteria and several new diagnostic categories [3, 5].

One of the newly introduced diagnoses is Prolonged Grief Disorder (PGD), which represents a formal classification of what was previously referred to as “complicated grief” [3, 6–13].

As discussed by Kristensen et al., Jordan et al., Nakajima et al., Eisma et al. and Prigerson et al. there is significant variation within reactions to bereavement and trajectories of symptoms [9, 10, 13–16]. Factors such as the circumstances of death, the closeness of the bond, available social support, and individual psychological predispositions all influence the grieving process [14]. Waves of grief can be triggered by reminders of the loss and, particularly in the initial phase of bereavement, are frequently characterized by intense longing [9, 14]. Normally, the intensity and frequency of grief symptoms, such as yearning for and preoccupation with the deceased, eventually diminish. Symptoms are reduced by reconnection with positive emotions and other persons: this is achieved through loss focused and restoration tasks which permit the integration of the reality of bereavement into the narrative of one’s life and allow an adaptation to new requirements of daily life resulting from the death of a beloved [9]. Several proposals for diagnostic entities describe constructs of complicated grief as the deviation of this recovery process and a state of “severe, persistent and disabling grief, which interferes with daily functioning” [13]. Among these, the diagnostic criteria for disordered grief proposed by the ICD-11 in the form of PGD requires the criteria outlined in Table 1 [13, 17].

**Table 1:** Diagnostic criteria for PGD per ICD-11 and DSM-5-TR. Adapted from Eisma [13]

ICD-11 PGD criteria	
A. Event criterion	History of bereavement following the death of a partner, parent, child or other person close to the bereaved.
B. Separation distress	A persistent and pervasive grief response characterized by one of the following symptoms: 1. Longing for the deceased 2. Persistent preoccupation of the deceased
C. Intense emotional pain	Accompanied by intense emotional pain, for example, sadness, guilt, anger, denial and blame Difficulty accepting the death Feeling that one has lost a part of one’s self An inability to experience positive mood Emotional numbness Difficulty engaging with social or other activities
D. Functional impairment criterion	The disturbance results in significant impairment in personal, family, social, educational, occupational or other important areas of functioning. If functioning is maintained, it is only through significant additional effort.
E. Cultural and time criteria	The pervasive grief response has persisted for an atypically long period of time following the loss, markedly exceeding expected social, cultural or religious norms for the individual’s culture and context. Grief responses lasting for less than 6 months, and for longer periods in some cultural contexts, should not be regarded as meeting this requirement.

The inclusion of PGD has drawn significant attention to the broader discussion around the medicalization of grief [18–21]. Critics argue that high PGD prevalence rates reflect overpathologization of natural responses to loss, while proponents counter that such claims often rely on prevalence data from high-risk subgroups rather than general populations [16]. Furthermore, questions have been raised about whether all mood alterations elicited by life events should be classified as mental disorders, or whether such classifications should be limited to conditions that impair functions essential for human life and are associated with significant individual harm [22]. There is concern that overly broad diagnostic categories may divert resources from individuals with severe psychiatric illnesses [23]. Prigerson et al. call for “the provision and dissemination of accurate information on the rates, risks, resolution, and treatment of PGD” [16] to “advance the field, provide precise and reliable diagnoses and estimates, and refer mourners to proven effective care” [16].

Relevant literature on PDG prevalence has been published over the past six years [17–21]. This scoping review aimed to assess how the diagnoses of PDG were established in prevalence research and what the prevalence was in different populations —reporting ranges of prevalence estimates, methodologies used, and research gaps identified [24]. Although several methodological elements mirror those of a systematic review, we did not perform a critical appraisal or meta-analysis, as our aim was to provide a field-wide mapping in line with scoping review methodology.

## Methods

### Search strategy and selection criteria

A protocol detailing the methodology of this scoping review was published in BMJ Open [24]. We follow the JBI Manual of Evidence Synthesis [25] and, with one exception, report in accordance with the PRISMA Extension for Scoping Reviews (PRISMA-ScR) [26]. We deviate by not reporting the aims of included studies. We assume prevalence studies on psychiatric disorders to mainly be published in journal articles or conference abstracts, with unpublished reports likely being dissertations or documents from national institutes. Therefore, MEDLINE, Embase, Web of Science, and PsycINFO were searched for primary research studies from the 1st of January 2011 to present on the 12th of April 2024. The lower bound of 2011 was selected as it marks the release of the ICD-11 alpha version, providing the first publicly accessible framework for PGD research [4]. No language restrictions were applied. The search strategy consists of three elements:

1. Components to retrieve results containing the term “Prolonged Grief Disorder” or variations.
2. Components to retrieve results containing the term “International Statistical Classification of Diseases and Related Health Problems 11” or variations.
3. Components to retrieve results containing terms like “observational, longitudinal, cross-sectional, epidemiology, prevalence, incidence, rate, polydiagnostic” or variations, to identify studies assessing prevalences.

The search term for all databases are included in the supplements.

Our grey literature search consisted of the following databases with their respective collections and websites:

- Web of Science:
  - The “ProQuest™ Dissertations & Theses Citation Index (PQDT)” collection.
  - The “Web of Science Core Collection” contains the “Conference Proceedings Citation Index - Science (CPCI-S)” as well as the “Conference Proceedings Citation Index– Social Sciences & Humanities (CPCI-SSH)” and as such identifies conference literature.
- Science.gov.
- NDLTD Global ETD Search.

Documentation of the search of Science.gov and the NDLTD Global ETD Search is provided below in the form of Supplement B.

As per our protocol, we included

- cross-sectional and longitudinal studies.
- assessing the prevalence of the mental, behavioural or neurodevelopmental disorders listed […].
- as per ICD-11 criteria.” [24].

In this research paper, (b) can be considered equivalent to “assessing the prevalence of PGD”.

The inclusion criterion “as per ICD-11 criteria” was applied as follows: during the title and abstract screening, we included all publications using the term PGD or explicitly stating the use of ICD-11 criteria. For data extraction, we included studies that used ICD-11 criteria as well as those with diagnostic modifications, excluding only those applying DSM-5 criteria. This approach served two purposes: first, what one study defined as “ICD-11 criteria” was often considered a modification in another, so we included both to enhance comparability; second, this allowed us to capture the variety of diagnostic approaches and to reflect the range of thresholds, highlighting the uncertainties and differing perspectives within the field.

Due to the time frame for this paper and expected response times, authors were not contacted for further information. The search results were exported to Rayyan, and deduplication was performed by KN. Title and abstract screening and full-text screening were conducted by two reviewers, KN and SK, according to inclusion and exclusion criteria. Disagreements were resolved by consensus of a third reviewer, namely SG.

Summary estimates were extracted from published reports or calculated, if sufficient information was reported by KN and reviewed by SK and SG. As required by the JBI Manual of Evidence, our data extraction tool is included as Supplement C. No assessment of study quality was performed. We contacted authors when publications were inaccessible and requested access. Documentation of contact attempts and outcomes is provided in Supplement D.

### Data analysis

Once the full-text screening was completed, our data extraction tool (Supplement C) was adapted based on the studies included and in accordance with the objectives of this paper.

Data were organized in tabular and diagrammatic form including a map for study locations. These suggestions were discussed with SG, and changes were made to improve data visualization.

### Role of the funding source

There was no funding source for this study.

## Results

**Fig. 1:**
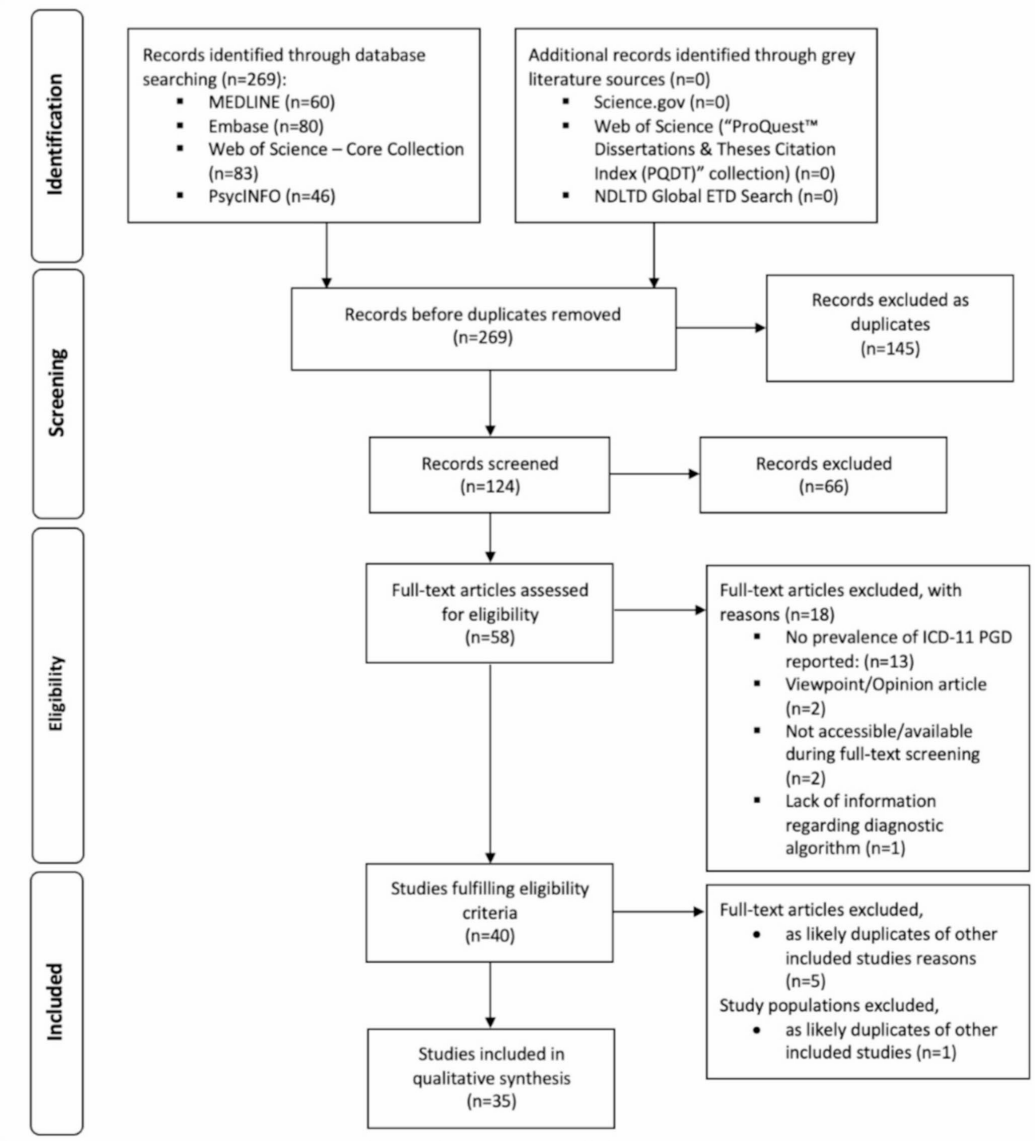
Flow chart of the screening process

Our comprehensive database search retrieved 269 results (Fig. 1). No relevant entries were found through grey literature sources. 145 duplicates were removed before title-abstract screening, and 66 publications were excluded. Thus, 58 publications underwent full-text screening. Of these, 18 were excluded for not meeting eligibility. Before data synthesis, five studies were removed to avoid data duplication, and one study population was excluded as it appeared in a newer included publication. A total of 35 studies were included for qualitative synthesis. Supplement E lists exclusions and reasons. Supplement F contains included studies with extracted data in table format.

Most studies were published between 2019 and 2024. Only 22 studies reported the time frame of data collection, with nearly half beginning between 2019 and 2021. Of the 35 publications, 31 applied a cross-sectional methodology, while four were longitudinal. For this synthesis, longitudinal projects reporting only one time point available were considered cross-sectional. Two publications, Lundorff et al. and Redican et al., likely originated from the same project [27, 28]. Therefore, three out of 34 study designs were considered to be longitudinal.

Study populations were categorized into:

1. General population (GP).
2. Non-representative community adults (NRCA).
3. Bereaved adults (BA).
4. Bereaved adults with intense/impaired grief (BA_IG).
5. Bereaved caregivers (BC).
6. Bereaved parents (BP).
7. Bereaved children and adolescents (BCA).

**Fig. 2:**
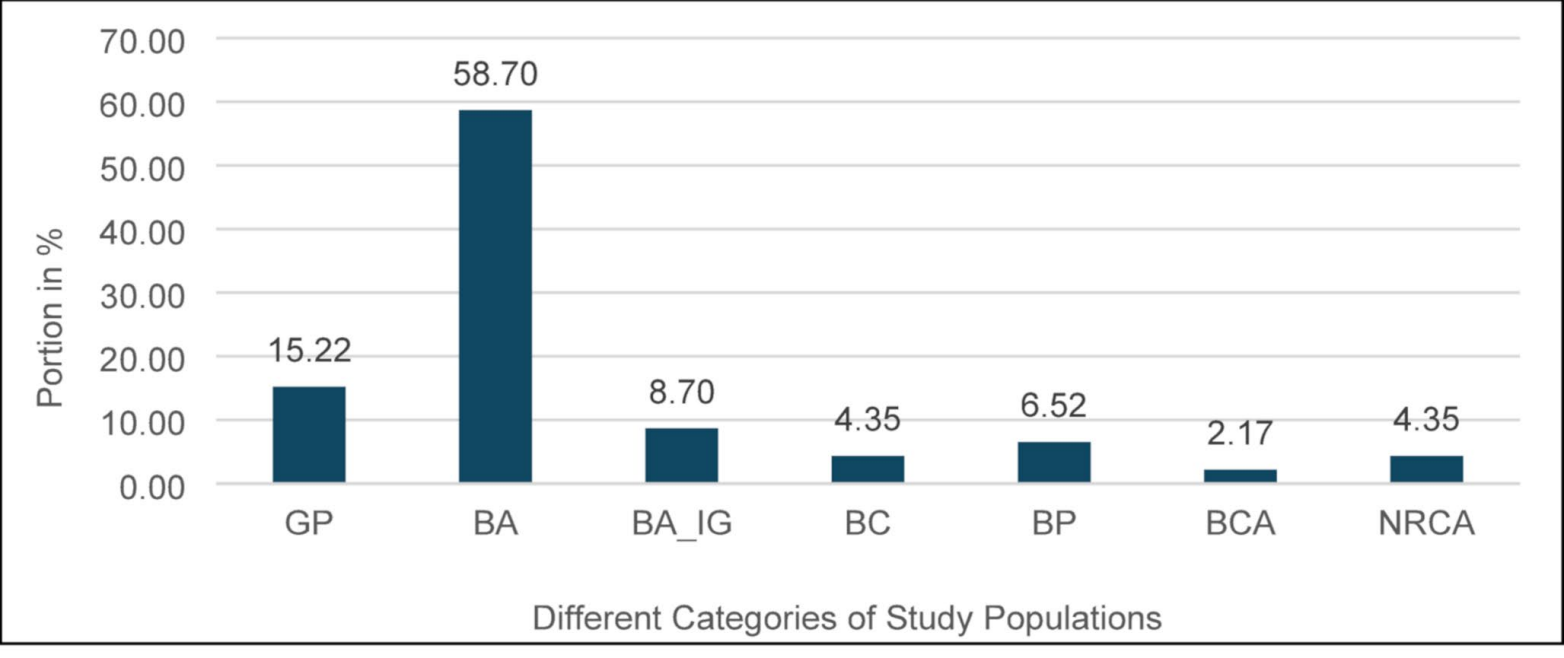
Proportion of different study populations among the included studies. Abbreviations: GP, general population; BA, bereaved adults; BA_IG, bereaved adults with intense/impaired grief; BC, bereaved caregivers; BP, bereaved parents; BCA, bereaved children and adolescents; NRCA, non-representative community adults

**Fig. 3:**
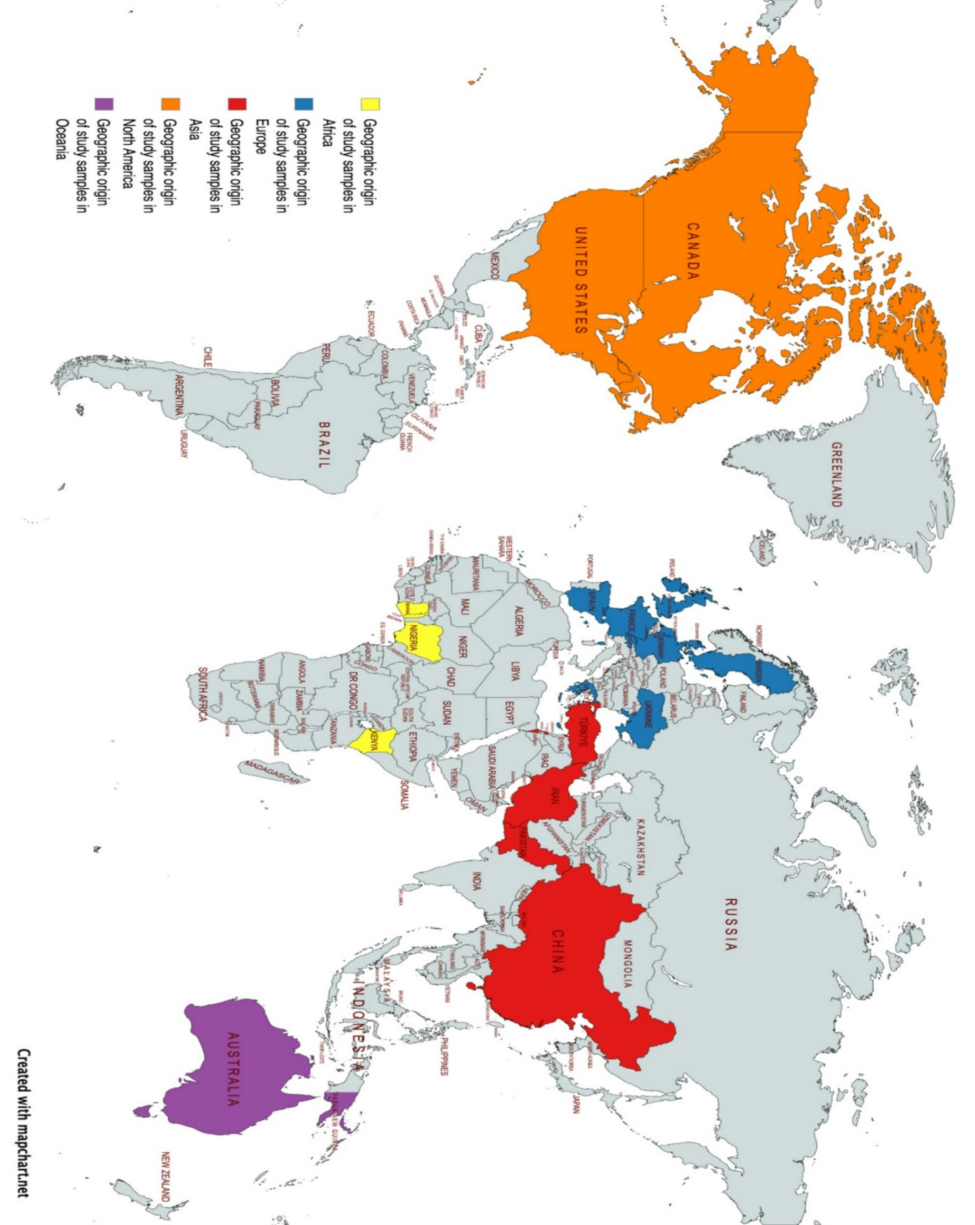
Geographic origin of study populations

**Fig. 4:**
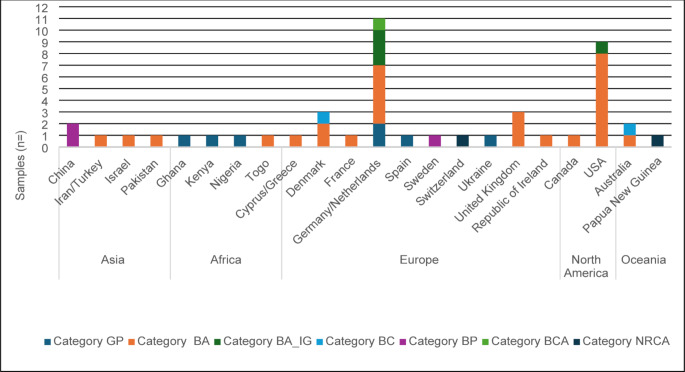
Geographic origin of study populations. Abbreviations: GP, general population; BA, bereaved adults; BA_IG, bereaved adults with intense/impaired grief; BC, bereaved caregivers; BP, bereaved parents; BCA, bereaved children and adolescents; NRCA, non-representative community adults

**Fig. 5:**
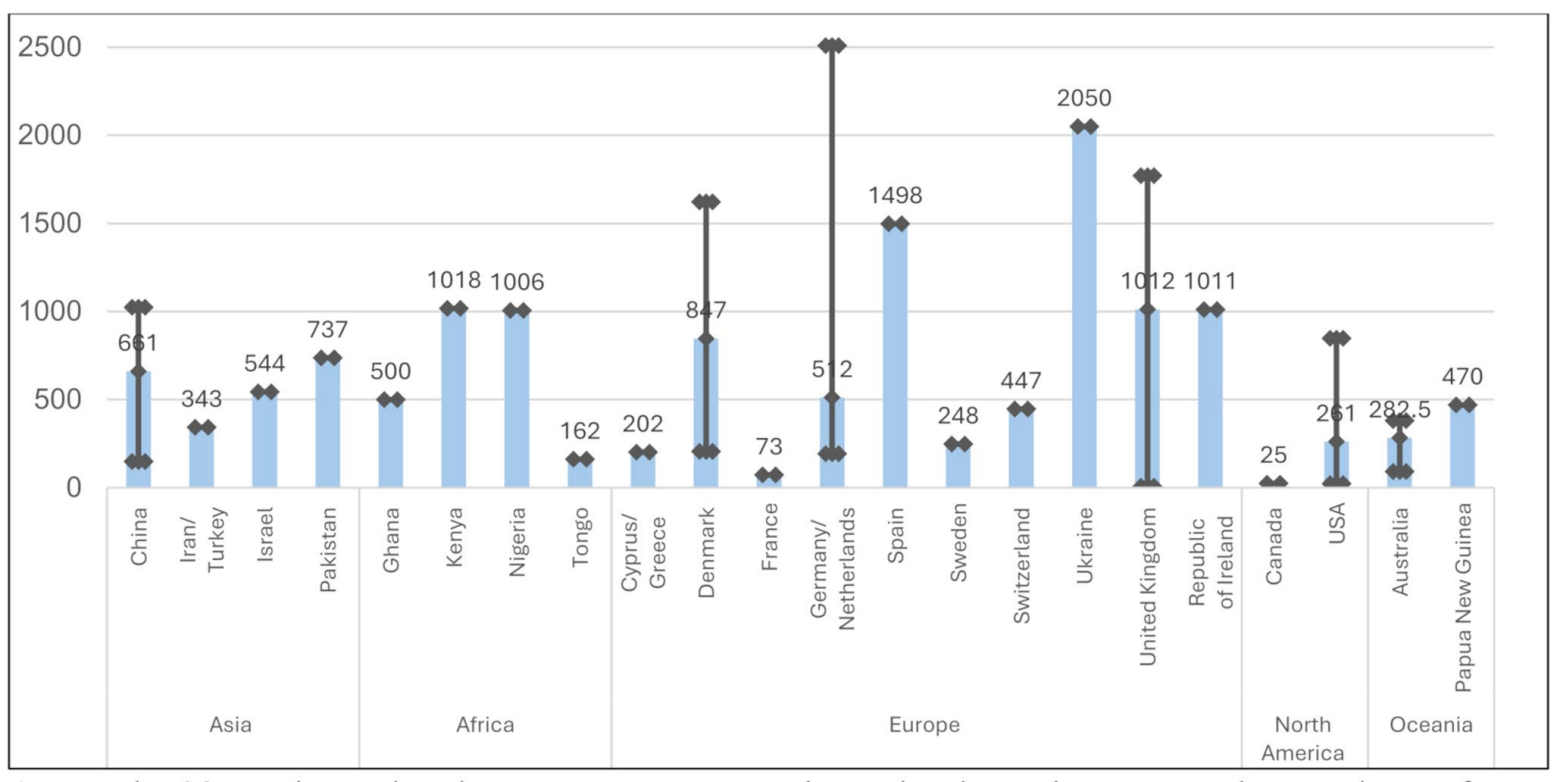
Sample size of study populations by geographic origin

The category “general population” consists of representative national samples. Studies on disturbed grief or treatment-seeking bereaved populations were categorized as “BA_IG,” and those on support groups were not automatically included under this label. Lundorff et al. and Redican et al. were considered one study, yielding 46 study populations from 34 publications (Fig. 2) [27, 28]. Over half consisted of samples of bereaved adults, and only one study represented bereaved children and adolescents. The geographic origin of study samples was visualized in Fig. 3. Figure 4 details the total number and categories of study populations by region. Figure 5 illustrates the range of and median sample size(s). Table 5 of Supplement F presents the percentage of female participants, mean age, mean time since loss, and the range of unnatural deaths across study populations by region. As several studies reported prevalence across two countries, we categorized them by geographic regions. Where no location was provided, institutional affiliation was used as a proxy, if deemed appropriate. Medians included all time points for longitudinal studies.

Among the 46 samples analyzed, seven were representative national samples. Data on the prevalence of Prolonged Grief Disorder (PGD) in the general population was available for Ghana, Kenya, Nigeria, Germany/Netherlands, Spain, and Ukraine. The grouping ‘Germany/Netherlands’ reflects multi-site studies that did not disaggregate data by country. This clustering avoids double counting. However, no general population samples were identified from Asia, North or South America, or Oceania. The only study assessing prevalence among bereaved children and adolescents came from the “Germany/Netherlands” region. In Asia, samples focused on bereaved parents and adults, while North American samples represented bereaved adults, both with and without impaired grief.

Sample characteristics varied across geographic regions. Female participants ranged from 33 to 92%. Median ages of participants spanned from 27 to 64 years, with the mean time since loss ranging from six to 268 months. Additionally, the proportion of bereavement due to unnatural deaths ranged from 0 to 100%. Sample sizes also showed wide variability, from as few as eight participants to as many as 2509. Of the 46 samples included, 24 were from Europe, with 11 originating specifically from the “Germany/Netherlands” region. North America contributed 10 samples, followed by Asia with five. Africa and Oceania were represented by four and three samples, respectively, while no samples were found from South America. Notably, for 16 out of 22 regions, data were drawn from a single study.

A variety of grief-specific measurement tools were used to assess PGD according to ICD-11 criteria (summarized in more detail in Table C of Supplement F). The most tools most frequently used were the Prolonged Grief Disorder (PG-13), the Inventory of Complicated Grief-Revised (ICG-R), and the International Prolonged Grief Disorder Questionnaire (IPGDS). Among the 43 applications of the tools, PG-13 accounts for 23%, followed by ICG-R at 21% and IPGDS at 9%. Other instruments used in the studies were the 15-item depression subscale from the Symptom-Checklist-90 (SCL depression), the Beck Depression Inventory (BDI-II), the Child PTSD Symptom Scale (CPSS), and the Children’s Depression Inventory (CDI).

To synthesize the diagnostic algorithms, we defined four classes of ICD-11 PGD assessments:

- ICD-11 PGD standard threshold as per ICD-11,
- ICD-11 PGD low diagnostic threshold,
- ICD-11 PGD high diagnostic threshold,
- and ICD-11 PGD combined low and high diagnostic thresholds.

The latter involves omitting some PGD criteria while raising symptom requirements for others. Table 2 summarizes the ICD-11 PGD algorithms. Tay et al. is excluded for lacking clear diagnostic criteria [29]. Out of 81 algorithms, only one shows a raised threshold for emotional pain symptoms. Eight assessments followed the ICD-11 algorithm without variations. 25 algorithms simplified the diagnostic procedure, mainly by omitting Criterion E’s culture component. The most common combination for a combined raised and lowered threshold was an elevated symptom for Criterion C coupled with the omission of the culture component in Criterion E.

**Table 2:** Diagnostic algorithms applied for ICD-11 PGD

ICD-11 PGD high diagnostic threshold	Number	Specific criterion raised	Number of assessments in which the scale score requirement was raised		
	1	• Criterion A: - • Criterion B: - • Criterion C: 1 • Criterion D: - • Criterion E (time criterion): - • Criterion E (cultural criterion):-	0		
ICD-11 PGD low diagnostic threshold	**Number**	**Criteria omitted for lowered diagnostic thresholds**	**Number of assessments in which the scale score requirement was lowered**		
	25	• Criterion A: - • Criterion B: - • Criterion C: 1 • Criterion D: - • Criterion E (time criterion): 1 • Criterion E (cultural criterion): 22	4		
ICD-11 PGD combined low and high diagnostic thresholds	**Number**	**Raised diagnostic threshold**	**Lowered diagnostic threshold**	**Criteria omitted for PGD assessments as per ICD-11 with a combined raised and lowered diagnostic threshold**	**Number of PGD assessments as per ICD-11 with a combined low and high diagnostic threshold with changes in scale score requirements**
	48	• Criterion A: • Criterion B: • Criterion C: 47 • Criterion D: • Criterion E (time criterion): 1 • Criterion E (cultural criterion):	• Criterion A: - • Criterion B: - • Criterion C: - • Criterion D: - • Criterion E (time criterion): - • Criterion E (cultural criterion): -	• Criterion A: • Criterion B: • Criterion C: • Criterion D: 10 • Criterion E (time criterion): 1 • Criterion E (cultural criterion): 48	0
ICD-11 PGD standard threshold as per ICD-11	**Number**				
	8				

Note: In the included studies, we distinguished between threshold types, showing whether diagnosis criteria were met by requiring more symptoms or a longer duration, or by using a higher scale score to confirm a symptom

**Fig. 6:**
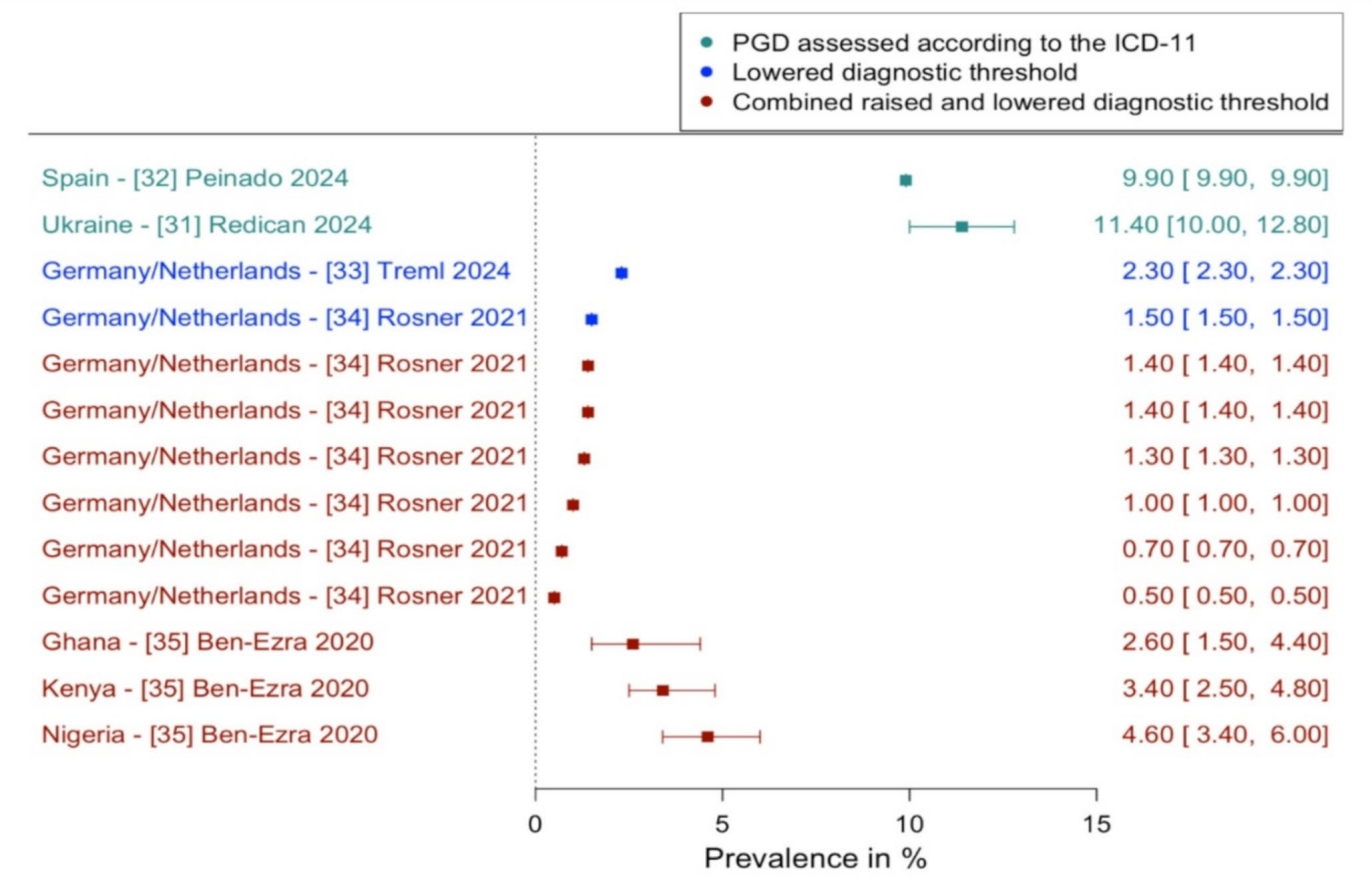
Prevalences of ICD-11 PGD in representative national samples. Note: The 95% confidence intervals are presented in square brackets. When the prevalence estimate and the 95% confidence interval are identical, the publication did not report a 95% CI. When multiple estimates are reported from the same study, they reflect the application of different diagnostic algorithms

All prevalence estimates from the study populations are provided in Supplement G, along with contextual information on the mean time between bereavement and assessment, which varies significantly from two (lowered threshold) to 134 months. Figure 6 exclusively focuses on prevalence estimates for the general population. The prevalence estimates for representative national samples assessed for PGD according to ICD-11 ranged from 9·9% to 11·4%. A lowered diagnostic threshold shows prevalences between 1·5% and 2·3%. A combined lowered and raised threshold results in rates from 0·5% to 4·6%. For Ghanaian, Kenyan, and Nigerian populations, rates range from 2·6% to 4·6% when applying a combined raised and lowered threshold. Nine prevalence estimates for PGD according to ICD-11 in bereaved adults (BA) range from 1·5% to 15·3%, the median being 6·9%. Diagnostic algorithms with a lowered threshold, result in prevalence estimates between 1·5% and 33%. For bereaved adults with impaired grief no applications of the criteria for PGD as per ICD-11 criteria were identified. Utilization of a lowered diagnostic threshold leads to prevalences between 19·2% and 95·8%. The median prevalence reported for bereaved caregivers was 7·3%, those for bereaved parents 29% and those for bereaved children and adolescents 27·7%.

Within the category NRCA the ICD-11 PGD prevalence for an adult community sample was reported by Eberle as lying at 0·22% [30]. As for longitudinal data, Boelen et al. assessed the prevalence of ICD-11 PGD within a sample of bereaved individuals at a mean time of 28·64 months (time point 1) and 25·65 months (time point 2) post-loss [31]. A decrease of 10% points in ICD-11 PGD prevalence from 18% to 8·2% within their sample of bereaved adults was observed. This notable change in mean time since loss resulted from dropouts between the assessments. Nielsen et al. report prevalences of 7·3% and 1·9% six months and three years post bereavement in a population of bereaved caregivers, Lundorff et al. and Redican et al. observed a decrease in ICD-11 PGD cases from 20·9% to 2·5% over 48 months using a lowered threshold [28, 32, 33].

## Discussion

In this systematic review of 35 studies involving 31,789 individuals, we found that prevalence estimates of PGD in national samples ranged from 0.5 to 11.4%. Most studies focused on bereaved adult samples from the United States, Germany, and the Netherlands. Notably, there were no studies from South America and no general population samples from Asia, highlighting a significant geographic gap in the literature. Only one study included a sample of bereaved children and adolescents.

A prominent finding was the wide variation in diagnostic approaches used across studies. Many studies modified the standard ICD-11 diagnostic thresholds, reflecting ongoing challenges in operationalizing disturbed grief. Similar to earlier debates surrounding the conceptualization of PTSD, the criteria for PGD continue to evolve and raise questions about the balance between universal diagnostic standards and cultural variations in symptom expression [34]. Long before ICD-11 PGD, several frameworks emerged: Horowitz et al. introduced the concept in the 1990s, followed by major diagnostic models from Prigerson et al., Shear et al., and Maciejewski et al. [35–38]. These frameworks required a higher threshold for emotional pain compared to ICD-11, specifying five, two, and three symptoms, respectively. Consequently, most ICD-11 PGD assessments that use a combined lowered and raised diagnostic threshold impose stricter requirements for Criterion C (Intense emotional pain). The omission of Criterion E’s cultural component further distinguishes ICD-11 PGD from earlier constructs, as this element is largely absent in the measurement tools used in the studies.

For this review, we categorized diagnostic approaches into four classes: ICD-11 PGD standard threshold, low diagnostic threshold, high diagnostic threshold, and combined low and high thresholds. While one might expect that lower thresholds would lead to higher prevalence estimates, this assumption did not hold consistently. For example, under ICD-11 criteria, representative national samples in Spain and Ukraine showed prevalence rates between 9.9% and 11.4%, whereas lower-threshold applications in Germany and the Netherlands yielded considerably lower estimates of 2.3% and 1.5%, respectively [32, 39–41]. This inconsistency highlights the research challenges posed by ICD-11’s lack of quantitative symptom thresholds, as single-word symptoms can lead to variable interpretations across screening tools. A meta-analysis by Lundorff et al. demonstrated a 17% discrepancy in pooled prevalence estimates depending on the instrument—such as the PG-13 or ICG—used to assess grief [18]. Similarly, Treml et al. found that existing instruments often fail to cover all ICD-11 criteria, with symptoms such as denial, blame, or inability to experience positive mood frequently omitted [21]. These inconsistencies in tools, symptom scales, and cut-offs contribute significantly to the variation in reported PGD prevalence.

Among bereaved adult samples, ICD-11 PGD prevalence estimates ranged from 1·5% to 15·3%, while using lower diagnostic thresholds led to higher estimates of 8·2–33% [27, 28, 31, 42, 43]. Although the lower diagnostic thresholds were generally associated with increased prevalence, the differences were relatively modest. Notably, the ranges for bereaved adult samples were comparable to those found in representative national samples. Beyond the issue of diagnostic tools, a broader problem in the literature is the lack of detailed sample characteristics. Critical information such as pre-loss mental health conditions (e.g., anxiety or depression), circumstances of death, and sociodemographic variables—including gender, education, and income—was frequently missing, limiting meaningful contextual interpretation [44]. As one example, PGD risk is 2.5 times higher in individuals who experienced a loss within the previous 6–12 months compared to those bereaved over a decade ago [45].

Several studies reported gender-specific distributions, with female participants often overrepresented (range 33–92%). Evidence suggesting gendered patterns in grief response and help-seeking behavior calls for future research to explicitly stratify prevalence by gender [46, 47]. Unfortunately, the limited granularity of data in existing studies precluded subgroup meta-analysis or robust interpretation in this review.

Despite ICD-11 Criterion E emphasizing cultural norms, only 8 out of 81 diagnostic algorithms in the included studies incorporated this criterion explicitly. This is a striking omission given that expressions and durations of grief are highly culture-dependent. The absence of studies from South America and limited coverage of Asia further limits insights into sociocultural moderators. Future research should aim to integrate culturally validated tools and assess sociocultural determinants such as religion, community structure, and grief-related stigma.

The limitations of this study have been previously discussed. Many of the included studies lacked comprehensive demographic data, including pre-loss mental health variables, which are likely to influence PGD prevalence. Geographic representation was skewed, with most studies conducted in Europe and North America, and a lack of data from South America and parts of Asia. The heterogeneity in diagnostic instruments and thresholds used also hindered direct comparability. Furthermore, the limited inclusion of children and adolescents restricts the generalizability of our findings to younger populations.

Concerns have been raised about the generalizability of scientific evidence supporting the ICD-11’s introduction of PGD as a distinct diagnostic category [13]. With some authors suggesting to abstain from new diagnostic categories and focus instead on established criteria of depression [22]. This review highlights the need for standardized ICD-11 PGD assessments within representative samples. Standardized reporting of demographic factors, such as gender, time since bereavement, and education, is crucial for meaningful comparisons, as these elements influence PGD risk. Utilizing tools like the IPGDS [48], which was specifically developed to assess ICD-11 PGD criteria, may help reduce methodological variability and improve the consistency of prevalence estimates. Moreover, systematic adjustments to core diagnostic parameters—such as the duration threshold or quantitative symptom requirements for Criterion C—should be empirically tested to establish the most reliable and valid diagnostic model [15]. Our findings support these recommendations and emphasize the importance of embedding future PGD research in longitudinal designs to better capture symptom trajectories over time.

Statements and Declarations.

## Electronic supplementary material

Below is the link to the electronic supplementary material.

Supplementary Material 1

## Data Availability

All data was obtained from published reports, and the extracted and synthesized content is fully presented in this manuscript and its supplementary materials.
