# Supplementary material for: The prevalence of prolonged grief disorder according to the international classification of diseases 11: a scoping review
Source: Eur Arch Psychiatry Clin Neurosci. 2025 Jun 25;276(5):2239–50. doi: 10.1007/s00406-025-02046-4 (PMC13481476; doi:10.1007/s00406-025-02046-4)
Supplement: Supplementary file 1 [file 406_2025_2046_MOESM1_ESM.pdf]

Supplementary Appendix

Supplement A:
- Search term 1: Embase via Ovid
- Search term 2: PsycINFO
- Search term 3: Web of Science

Supplement B:
- Table 1: Documentation of NDLTD Global ETD Search search
- Table 2: Screening of entries identified through the search of NDLTD_Global_ETD_Search
- Table 3: Documentation of Science.gov search

Supplement C
- Data Extraction Form

Supplement D
- Table 1: Documentation of contact attempts

Supplement E
- Table 1: Publications excluded during full-text screening due to non-fulfillment of eligibility criteria
- Table 2: Publications excluded as likely duplicates of other included studies
- Table 3: Study populations excluded as likely duplicates of other included studies

Supplement F
- Table 1: Items 1-6a of "Table 3: Elements of the utilized data extraction tool for every included study"
- Table 2: Items 1a-b, 5, 6b-c, and 7a of "Table 3: Elements of the utilized data extraction tool for every included study"
- Table 3: Items 1a-b, 5, and 7b-j of "Table 3: Elements of the utilized data extraction tool for every included study"
- Table 4: Items 1a-b and 8-11 of "Table 3: Elements of the utilized data extraction tool for every included study"
- Table 5: Demographic characteristics of study samples by geographic origin

Supplement G

- Table 1A: Prevalence estimates from the category "general population"
- Table 1B: Mean, IQR, Q1 and Q3 for the category "general population" for all estimates and divided by application of ICD-11 criteria
- Table 2A: Prevalence estimates for the category "bereaved adults"
- Table 2B: Mean, IQR, Q1 and Q3 for the category "bereaved adults" for all estimates and divided by application of ICD-11 criteria
- Table 3A: Prevalence estimates from the category "bereaved adults with impairing/intense grief"
- Table 3B: Mean, IQR, Q1 and Q3 for the category "bereaved adults with impairing/intense grief" for all estimates and divided by application of ICD-11 criteria
- Table 4: Prevalence estimates from the category "bereaved caregivers"
- Table 5A: Prevalence estimates from the category "bereaved parents"
- Table 5B: Mean, IQR, Q1 and Q3 for the category "bereaved parents" for all estimates and divided by application of ICD-11 criteria
- Table 6: Prevalence estimates from the category "bereaved children and adolescents"
- Table 7: Prevalence estimates for the category "non-representative community adults"

Supplement A

Search term 1: MEDLINE via Ovid

- Prolonged Grief Disorder

  (grie* OR mourn* OR "complex-bereavement").ti,ab. OR Exp Bereavement/

- ICD-11

  (("International Classification of Disease?" Adj3 11) OR ICD-11 OR ICD11 OR ("International Statistical Classification of Disease?" adj3 11) or ICD-Eleven).ti,ab. OR International Classification of Diseases

- Observational Studies[1]

  Observational Studies:
  "observation* stud*".ti,ab,kw. or observational study/

  Cohort studies:
  (cohort or longitudinal or follow-up or prospective or "concurrent stud*").ti,ab,kw. Or cohort studies/ or longitudinal studies/ or follow-up studies/ or prospective studies/

  Cross sectional studies:
  (cross-sectional or "disease frequenc*" or transversal or prevalence).ti,ab,kw.or Cross-Sectional Studies/ or Prevalence/ or Epidemiologic Measurements/

  Epidemiology:
  Epidemiolog*.ti,ab,kw. or epidemiology/

  Incidence:
  (incidence or rate).ti,ab,kw. or incidence/

  Polydiagnostic Studies:
  Polydiagnostic.ti,ab,kw.

The applied search strategy consists of three elements combined by the operator "and".
The search element "observational studies" is an adaptation of a Search Filter. The components of the search element "observational studies" were separated by the search operator "or".

Search term 2: Embase via Ovid

- Prolonged Grief Disorder

  (grie* OR mourn* OR "complex-bereavement").ti,ab. OR Exp Grief/ OR Bereavement/

- ICD-11

  (("International Classification of Disease?" Adj3 11) OR ICD-11 OR ICD11 OR ("International Statistical Classification of Disease?" adj3 11) or ICD-Eleven).ti,ab. OR International Classification of Diseases/ OR ICD-11/

- Observational Studies[1]

  Observational Studies:

"observation* stud*".ti,ab,kw. or observational study/

Cohort studies:
(cohort or longitudinal or follow-up or prospective or "concurrent stud*").ti,ab,kw. or cohort analysis/ or longitudinal study/ or follow up/ or prospective study/

Cross sectional studies:
(cross-sectional or "disease frequenc*" or transversal or prevalence).ti,ab,kw. or cross-sectional study/ or prevalence/ or lifetime prevalence/ or period prevalence/ or point prevalence/ or prevalence ratio/

Epidemiology:
Epidemiolog*.ti,ab,kw. or epidemiology/

Incidence:
(incidence or rate).ti,ab,kw. or incidence/

Polydiagnostic Studies:
Polydiagnostic.ti,ab,kw.

The applied search strategy consists of three elements combined by the operator "and".
The search element "observational studies" is an adaptation of a Search Filter. The components of the search element "observational studies" were separated by the search operator "or".

Search term 3: PsycINFO

- Prolonged Grief Disorder

  TI ("grie*" OR "mourn*" OR "complex-bereavement") OR AB ("grie*" OR "mourn*" OR "complex-bereavement") OR DE "Prolonged Grief Disorder" OR DE "Bereavement"

- ICD-11

  TI (("International Classification of Disease#" N2 "11") OR "ICD-11" OR "ICD11" OR ("International Statistical Classification of Disease#" N2 "11") or " ICD-Eleven") OR AB (("International Classification of Disease#" N2 "11") OR "ICD-11" OR "ICD11" OR ("International Statistical Classification of Disease#" N2 "11") or " ICD-Eleven") OR DE "International Classification of Diseases"

- Observational Studies[1]

  Observational Studies:
  TI ("observation* stud*") OR AB ("observation* stud*") OR KW ("observation* stud*")

  Cohort studies:
  TI ("cohort" or "longitudinal" or "follow-up" or "prospective" or "concurrent stud*") OR AB ("cohort" or "longitudinal" or "follow-up" or "prospective" or "concurrent stud*") OR KW ("cohort" or "longitudinal" or "follow-up" or "prospective" or "concurrent stud*") OR DE "Cohort Analysis" OR DE "Longitudinal Studies" OR DE "Prospective Studies" OR DE "Followup Studies"

  Cross sectional studies:

TI ("cross-sectional" or "Disease Frequenc*" or "transversal" or "prevalence") OR AB ("cross-sectional" or "Disease Frequenc*" or "transversal" or "prevalence") OR KW ("cross-sectional" or "Disease Frequenc*" or "transversal" or "prevalence")

Epidemiology:
TI ("Epidemiolog*") OR AB ("Epidemiolog*") OR KW ("Epidemiolog*") OR DE "Epidemiology"

Incidence:
TI ("incidence" or "rate") OR AB ("incidence" or "rate") OR KW ("incidence" or "rate")

Polydiagnostic Studies:
TI ("polydiagnostic") OR AB ("polydiagnostic") OR KW ("polydiagnostic")

The applied search strategy consists of three elements combined by the operator "and".
The search element "observational studies" is an adaptation of a Search Filter. The components of the search element "observational studies" were separated by the search operator "or".

Search term 4: Web of Science

- Prolonged Grief Disorder

  ("grie*" OR "mourn*" OR "complex-bereavement") (Topic)

- ICD-11

  (("International Classification of Disease$" NEAR/2 "11") OR "ICD-11" OR "ICD11" OR ("International Statistical Classification of Disease$" NEAR/2 "11") or "ICD-Eleven") (Topic)

- Observational Studies[1]

  Observational Studies:
  ("observation* stud*") (Topic)

  Cohort studies:
  ("cohort" or "longitudinal" or "follow-up" or "prospective" or "concurrent stud*") (Topic)

  Cross sectional studies:
  ("cross-sectional" or "disease frequenc*" or "transversal" or "prevalence") (Topic)

  Epidemiology:
  ("epidemiolog*") (Topic)

  Incidence:
  ("incidence" or "rate") (Topic)

  Polydiagnostic Studies:
  ("polydiagnostic") (Topic)

The applied search strategy consists of three elements combined by the operator "and".
The search element "observational studies" is an adaptation of a Search Filter. The components of the search element "observational studies" were separated by the search operator "or".

References

1. Search Filters for Various Databases: Ovid Medline [Internet]. Search Filters for Various Databases. [cited 2024 Jan 15]. Available from: https://libguides.sph.uth.tmc.edu/search_filters/ovid_medline_filters

Supplement B:

Table 1: Documentation of NDLTD Global ETD Search search

Name of resource	URL	Date searched	Searcher	Pathway followed (Browsed Headings and Terms searched on the respective sites within the website)
NDLTD Global ETD Search	http://search.ndltd.org/	04/13/2024	KN	• The following term was searched for: title:"International Classification of Diseases 11" or title:"ICD-11" or title:"ICD 11" or title:"ICD11" or title:"International Statistical Classification of Diseases 11" or title:"ICD-Eleven" or description:"International Classification of Diseases 11" or description:"ICD-11" or description:"ICD 11" or description:"ICD11" or description:"International Statistical Classification of Diseases 11" or description:"ICD-Eleven"<br>• The search was limited to entries with a publication year between 2011 and 2024<br>• The following entries were identified:<br>○ Evaluating clinicians' differential diagnostic decisions for ICD-11 psychotic disorders *Peterson, Destiny Lynn 09 May 2015 (has links)*<br>○ A Case study of a new era in disease classification: an investigation of the socio-technical requirements for inclusive standardization development. *Bougie, Gargi 26 April 2012 (has links)*<br>○ Psykoterapi med patienter med komplex PTSD : - en intervjustudie / Psychotherapy for complex PTSD : - an interwiew study *Järnvall, Charlotte, Hanquist, Lisa January 2022 (has links)*<br>○ Pathways Linking Clinician Demographics to Mental Health Diagnostic Accuracy: An International Perspective *Brechbiel, Julia 01 January 2017 (has links)*<br>○ Klinický obraz vývojové dysfázie v konceptu neurovývojových poruch / The clinical picture of developmental dysphasia in the concept of neuorodevelopmental disorders *Pospíšilová, Lenka January 2022 (has links)*<br>○ Incongruência de Gênero : um estudo comparativo entre os critérios diagnósticos CID-10, CID-11 e DSM-5 *Soll, Bianca Machado Borba January 2016 (has links)*<br>○ Incongruência de Gênero : um estudo comparativo entre os critérios diagnósticos CID-10, CID-11 e DSM-5 *Soll, Bianca Machado Borba January 2016 (has links)*<br>○ Incongruência de Gênero : um estudo comparativo entre os critérios diagnósticos CID-10, CID-11 e DSM-5 *Soll, Bianca Machado Borba January 2016 (has links)*<br>○ Women-specific mental disorders in DSM-V: are we failing again? *Wittchen, Hans-Ulrich 20 February 2013 (has links)*<br>○ Zařazení žáků s Aspergerovým syndromem do běžné třídy na prvním stupni základní školy / Inclusion of Pupils with Asperger syndrome in primary school *Belhajová, Hana January 2020 (has links)*<br>○ Therapie der Binge-Eating-Störung *Hilbert, Anja 27 September 2022 (has links)*<br>○ Complex trauma and the influence of emotional regulation and interpersonal problems : a review of Complex-PTSD and an empirical study in a prison setting *Browne, Richard January 2017 (has links)*<br>○ Äldre personers erfarenheter av depressiva tillstånd i ålderdomen: En systematisk litteraturstudie *Pettersson, Joel, Kornhall, Sunna January 2019 (has links)*<br>○ The Study of SNOMED CT Applies to the Medical Facilities / SNOMED CT應用於醫療機構之研究*Song-yang Huang, 黃頌揚 January 2012*<br>○ Psychological predictors of post-traumatic stress disorder in children and adolescents *Memarzia, Jessica January 2018 (has links)*

				○ The Position of Anxiety Disorders in Structural Models of Mental Disorders *Wittchen, Hans-Ulrich, Beesdo, Katja, Gloster, Andrew T. 23 April 2013*<br>○ Gender dysforie u osob s poruchou autistického spektra / Gender dysphoria in persons with autism spectrum disorder *Lukina, Julie January 2020*<br>○ Unga individers upplevelser av hur gaming påverkar deras hälsa : En litteraturöversikt *Fluur, Kajsa, Kaveh, Maria January 2023*<br>○ *Autism spectrum disorders:an epidemiological and clinical study Mattila, M.-L. (Marja-Leena) 21 January 2014*<br>○ *Cultural Encounters in Medicine: (Re)Constituting Traditional Medicine in Taiwan under Colonization, Modernity, and Exchange Tsai, Hung-Yin 04 August 2021*<br>○ *APPLYING EVIDENCE MAPPING METHODOLOGIES TO THE WORLD HEALTH ORGANIZATION'S TUBERCULOSIS GUIDELINES Hajizadeh, Anisa January 2020*<br>○ *A espiritualidade na obra de autores da psicologia, saúde e educação Lousada, Márcia Gouvêa 22 September 2017*<br>○ *Global mortality attributable to alcoholic cardiomyopathy Manthey, Johann Jakob 04 September 2020*<br>○ *Från sorg till professionellt samtalsstöd i sorgearbetet efter en närståendes död : - En kvalitativ studie utifrån fem sörjandes upplevelser. / From grief to use of professional therapeutic conversation in mourning the death of a loved one : - A qualitative study of the experiences of five bereaved. Elfström, Teresa January 2012*

Table 2: Screening of entries identified through the search of NDLTD_Global_ETD_Search

Entry	Description	Inclusion/Exclusion	Reason for Exclusion
1. Evaluating clinicians' differential diagnostic decisions for ICD-11 psychotic disorders *Peterson, Destiny Lynn 09 May 2015 (has links)*	The ICD is currently under revision and this study is the first phase of the electronic field trials for ICD-11 for psychotic disorders. The present study compared ICD-10 and ICD-11 with regards to changes made to the diagnostic guidelines and changes amongst specific disorders. Of specific interest was clinicians' ability to accurately diagnose disorders that can present with overlapping features. We found that both diagnostic systems were accurate in differentiating disorders that have the potential to be easily confused. For some of the diagnostic guidelines that were undergoing substantial changes from ICD-10 to ICD-11, we did find improvements in the proposed guidelines. Subsequent studies will expand on our findings prior to the release of ICD-11. However, based on our initial findings, the proposed changes do appear to be increasing clinical utility of the diagnostic guidelines.	Exclusion	The topic of PGD is not addressed.
2. A Case study of a new era in disease classification: an investigation of the socio-technical requirements for inclusive standardization development. *Bougie, Gargi 26 April 2012 (has links)*	Until recently, the development and maintenance of the standard international disease classification for diagnostic, epidemiological and health management purposes has been handled by a closed group of experts with little input from other members of the medical community, interested organizations, or patient groups. The eleventh revision of the World Health Organization's International Classification of Diseases (ICD-11) represents an attempt to involve a much broader stakeholder group in the process of redesigning a standardized classification. Our research is an exploratory case study of this revision effort. We examine the socio-technical ecosystem of the ICD-11 project and produce a set of five recommendations for developing inclusive standardization systems. These recommendations are supported by an analysis of two additional projects in the health information and informatics domain, as well as a varied collection of literature. Our first recommendation implores system designers to consider technology-readiness and collaboration-readiness. We also advocate for the support of articulation and coordination work, and address the need for a distinct purpose and clearly defined process surrounding any introduced technology. Finally, we shed light on the need for incremental openness when attempting to involve a wide audience of stakeholders in the development process.	Exclusion	Not an observational study assessing the prevalence of PGD according to ICD-11.

3.	Psykoterapi med patienter med komplex PTSD : - en intervjustudie / Psychotherapy for complex PTSD : - an interwiew study *Järnvall, Charlotte, Hanquist, Lisa January 2022 (has links)*	Komplex PTSD har tidigare gått under samma kriterier och riktlinjer för behandling som PTSD, men togs 2018 in som en egen diagnos efter att WHO gjort revideringar inför utgåvan ICD-11. I Sverige finns ännu inga specifika riktlinjer för behandling av komplex PTSD. Syftet med denna studie var att undersöka hur psykoterapeuter uppfattar patienter med komplex PTSD, om och i så fall hur de anpassar behandlingsinterventioner till patientgruppen. Sju erfarna psykoterapeuter intervjuades. Rekrytering av deltagare skedde via egna arbetsrelaterade kontakter, som i sin tur kunde ge förslag om kvalificerade deltagare. Psykoterapeuterna intervjuades utifrån en semistrukturerad intervjuguide. Tematisk analys applicerades på intervjumaterialet, vilket mynnade ut i sju huvudteman. Resultaten visade att psykoterapeuterna såg tydlig skillnad mellan patienter med PTSD och patienter med komplex PTSD, i enlighet med differentieringen i ICD-11. Dissociation beskrevs också som ett framträdande symtom och som behandlingen behöver inriktas mot. Psykoterapeuterna beskrev att de i terapiprocessen anpassade och modifierade interventioner och var följsamma inför patienternas behov. Psykoterapiprocessen beskrevs i termer av "pendling" och "spiraler" snarare än sekventiellt med stabiliseringsfas, exponeringsfas och integreringsfas i en följd. Studiens resultat stämmer överens med förslag från ny forskning som rekommenderar en flexibel patientcentrerad multi-komponentbehandling som inriktas på de specifika symtomklustren vid komplex PTSD. Att arbeta i team beskrevs av psykoterapeuterna som en viktig bidragande faktor till kompetensutveckling samt ökade deras förmåga att använda verksamma metoder i arbetet med patienter med komplex PTSD. / The diagnosis complex PTSD has previously been subject to the same criteria and guidelinesfor treatment as PTSD. Complex PTSD was introduced in 2018 as its own diagnosis, when theWHO made revisions for the release of ICD-11. In Sweden, there are no specific guidelines forthe treatment of complex PTSD. The purpose of this study was to investigate howpsychotherapists perceive patients with complex PTSD and how they adapt treatmentinterventions to the patient group. Seven experienced psychotherapists were interviewed.Recruitment of participants was done via strategic selection. The psychotherapists wereinterviewed from a semi-structured interview guide. Thematic analysis was applied to theinterview material, which resulted in seven main themes. The results showed thatpsychotherapists saw a clear difference between PTSD and complex PTSD, as described in ICD-11. They also described dissociation as a prominent symptom. The psychotherapistsdescribed that they adapted and modified interventions and were keen to be compliant with theneeds of patients. The therapeutic process was described in terms "spirals" rather thansequentially. The study's findings are consistent with suggestions from new researchrecommending a flexible patient-centered multi-component treatment that targets the specificsymptom clusters of complex PTSD. Working in teams was described as an important contributing factor to competence development and increased the potential to use active methods with patients with complex PTSD.	Exclusion	Not an observational study assessing the prevalence of PGD according to ICD-11.
4.	Pathways Linking Clinician Demographics to Mental Health Diagnostic Accuracy: An International Perspective *Brechbiel, Julia 01 January 2017 (has links)*	Significant research efforts have focused on examining the effect of patient factors on providing diagnoses across clinical settings; however, the influence of clinician demographics have received less attention. This study aimed to understand the impact of nonclinical factors such as clinician characteristics and response time on diagnostic accuracy. The study used data from a WHO field study of the ICD-11 development (n = 1822) that required clinicians to diagnose two case vignettes. Clinicians' slower response times had a significant positive impact on their rates of diagnostic accuracy. However, there was no evidence that clinicians' demographic features were directly related to their diagnostic accuracy. Rather, clinicians' age, years of experience, world region, and their clinical profession indirectly predicted accuracy through their overall response time. Contradictory to decision-making theories, older clinicians and clinicians with more years of experience had higher rates of diagnostic accuracy when they spent more time completing the study. Additionally, clinicians in South America with slower response times had higher accuracy compared to clinicians in North America. Clinicians in Asia had faster response times that negatively impacted their accuracy rates compared to North America clinicians. These findings suggest differences in response time and the applicability of the ICD-11 across cultures. Consistent with previous research, medical professionals with quicker response times had the lowest rates of accuracy compared to psychologists and other clinical professionals. These findings highlight the need for researchers and clinicians to consider the role their dispositional features have in the diagnostic process. Moreover, it is crucial that future research into diagnostic decision-making and accuracy should consider additional mediating factors such as response style, culture, and experience.	Exclusion	Not an observational study assessing the prevalence of PGD according to ICD-11.
5.	Klinický obraz vývojové dysfázie v konceptu neurovývojových poruch / The clinical picture of developmental dysphasia in the concept of neurodevelopmental disorders *Pospíšilová,*	Introduction: Developmental dysphasia (DD) is a developmental language disorder with high prevalence, but also with low publication index and confusion of the terminology (including the Czech version of ICD - "speech" disorder instead of "language" disorder). The scientific view has passed the development from a symptom, through the specific nozologic unit to the current term Developmental language disorder (DLD) and its classification among a new category of diseases in ICD-11 (existing in DSM-5) called Neurodevelopmental disorders. Another disorder with the characteristic pathology in communication is the autism spectrum disorder (ASD) which is classified as a disease of the same category as DD. Compared to DD, it has a higher publication index, lower prevalence, and even more serious consequences. Three different domains that cause confusion, both in ASD and DD, are "speech", "language", and "communication". The same confusion applies to the current subtypes in ICD-11 with the term "functional language". In the Czech	Exclusion	Not an observational study assessing the prevalence of PGD according to ICD-11.

	Lenka January 2022 (has links)	Republic there are very few studies dealing with the patients with DD, their profile of a clinic picture has not been processed so far. In spite of the increase of Czech research of ASD there is a missing speech, language, and communication profile of these individuals. This has been a subject of a long-term and so far unfinished discussion in the world of science. The aim of our study was to describe the clinic picture of the children with DD, i.e. to create the speech, language, and communication profile, to apply analogical procedures among children with ASD and to find the mutual relationship between the diagnoses by comparing those profiles		
6.	Incongruência de Gênero : um estudo comparativo entre os critérios diagnósticos CID-10, CID-11 e DSM-5 *Soll, Bianca Machado Borba January 2016 (has links)*	A presente dissertação tem o objetivo de discutir a proposta dos critérios diagnósticos da CID-11 para Incongruência de Gênero e comparar as diretrizes dos manuais diagnósticos DSM-5 e CID-10 para Disforia de Gênero e Transtorno de Identidade de Gênero, respectivamente. A Organização Mundial da Saúde (OMS) está em processo de revisão da Classificação Internacional de Doenças (CID). Diferentemente do sistema de classificação vigente (CID-10), as modificações propostas pela CID-11 no que diz respeito à condição transexual são norteadas pela compreensão de que esta não é doença mental e que o acesso à saúde desta população necessita ser ampliado. O artigo derivado desta dissertação compara os critérios nos manuais diagnósticos existentes, o DSM-5 e da CID-10, em uma amostra brasileira de pessoas transexuais que procuraram serviços de saúde especificamente para a transição física. Este é um estudo transversal multicêntrico que inclui uma amostra de 103 indivíduos que procuraram os serviços em um dos dois principais centros de referência no Brasil especializados em identidade de gênero. O método da pesquisa consiste na aplicação, por profissionais previamente treinados, de uma entrevista estruturada desenvolvida pelo WHO´s Field Study Coordination Group for ICD-11 Mental and Behavioural Disorders que contempla os critérios diagnósticos. Os resultados revelam que, embora exista desacordo teórico nos critérios há uma sobreposição entre os dois sistemas quanto à confirmação do diagnóstico, com o DSM-5 mais inclusivo. Adicionalmente, a média do tempo de espera para ter acesso a este tipo de serviço é de quase uma década. Nossos achados confirmam a ideia de que há pouco consenso quanto aos critérios diagnósticos dos comportamentos transgêneros, considerando a diversidade de contextos sociais e culturais e que seguem com pouca diferenciação tanto etiológica quanto clínica para fins diagnósticos. / The current work aims to discuss the proposed diagnostic criteria of ICD-11 for Gender Incongruence and compare the diagnostic criteria of DSM-5 and ICD-10 Gender Dysphoria and Gender Identity Disorder, respectively. The World Health Organization (WHO) is reviewing the International Classification of Diseases (ICD). Despite the existing classification system (ICD-10), changes proposed by ICD-11 concerning transgender condition are guided by the understanding that it is not a mental illness and that this population needs health service access to be expanded. The study derived from this work aim to compare the criteria in the existing diagnostic manuals, the DSM-5 and the ICD-10, among a Brazilian sample of transgender persons who sought health services specifically for physical transition. This is a multicenter cross-sectional study that includes a sample of 103 subjects who sought services for gender identity disorder in one of two main reference centers in Brazil. The research method consists of applying a structured interview, which is comprised of the diagnostic criteria from the two manuals. The results reveal that although the theoretical disagreement in the criteria, there is an overlap among the two systems as diagnosis confirmation, to the DSM-5 more inclusive. Additionally, the average waiting time to access this type of service is nearly a decade. Although there is not a consensus concerning such on transgenderism in the diversity of social and cultural contexts, the findings confirm previous impression that despite efforts to determine the diagnostic settings, they follow slightly different as to etiology and different clinical presentations of this condition.	Exclusion	Not an observational study assessing the prevalence of PGD according to ICD-11.
7.	Incongruência de Gênero : um estudo comparativo entre os critérios diagnósticos CID-10, CID-11 e DSM-5 *Soll, Bianca Machado Borba January 2016 (has links)*	A presente dissertação tem o objetivo de discutir a proposta dos critérios diagnósticos da CID-11 para Incongruência de Gênero e comparar as diretrizes dos manuais diagnósticos DSM-5 e CID-10 para Disforia de Gênero e Transtorno de Identidade de Gênero, respectivamente. A Organização Mundial da Saúde (OMS) está em processo de revisão da Classificação Internacional de Doenças (CID). Diferentemente do sistema de classificação vigente (CID-10), as modificações propostas pela CID-11 no que diz respeito à condição transexual são norteadas pela compreensão de que esta não é doença mental e que o acesso à saúde desta população necessita ser ampliado. O artigo derivado desta dissertação compara os critérios nos manuais diagnósticos existentes, o DSM-5 e da CID-10, em uma amostra brasileira de pessoas transexuais que procuraram serviços de saúde especificamente para a transição física. Este é um estudo transversal multicêntrico que inclui uma amostra de 103 indivíduos que procuraram os serviços em um dos dois principais centros de referência no Brasil especializados em identidade de gênero. O método da pesquisa consiste na aplicação, por profissionais previamente treinados, de uma entrevista estruturada desenvolvida pelo WHO´s Field Study Coordination Group for ICD-11 Mental and Behavioural Disorders que contempla os critérios diagnósticos. Os resultados revelam que, embora exista desacordo teórico nos critérios há uma sobreposição entre os dois sistemas quanto à confirmação do diagnóstico, com o DSM-5 mais inclusivo. Adicionalmente, a média do tempo de espera para ter acesso a este tipo de serviço é de quase uma década. Nossos achados confirmam a ideia de que há pouco consenso quanto aos critérios diagnósticos dos comportamentos transgêneros, considerando a diversidade de contextos sociais e culturais e que seguem com pouca diferenciação tanto etiológica quanto clínica para fins diagnósticos. / The current work aims to discuss the	Exclusion	Not an observational study assessing the prevalence of PGD according to ICD-11.

		proposed diagnostic criteria of ICD-11 for Gender Incongruence and compare the diagnostic criteria of DSM-5 and ICD-10 Gender Dysphoria and Gender Identity Disorder, respectively. The World Health Organization (WHO) is reviewing the International Classification of Diseases (ICD). Despite the existing classification system (ICD-10), changes proposed by ICD-11 concerning transgender condition are guided by the understanding that it is not a mental illness and that this population needs health service access to be expanded. The study derived from this work aim to compare the criteria in the existing diagnostic manuals, the DSM-5 and the ICD-10, among a Brazilian sample of transgender persons who sought health services specifically for physical transition. This is a multicenter cross-sectional study that includes a sample of 103 subjects who sought services for gender identity disorder in one of two main reference centers in Brazil. The research method consists of applying a structured interview, which is comprised of the diagnostic criteria from the two manuals. The results reveal that although the theoretical disagreement in the criteria, there is an overlap among the two systems as diagnosis confirmation, to the DSM-5 more inclusive. Additionally, the average waiting time to access this type of service is nearly a decade. Although there is not a consensus concerning such on transgenderism in the diversity of social and cultural contexts, the findings confirm previous impression that despite efforts to determine the diagnostic settings, they follow slightly different as to etiology and different clinical presentations of this condition.		
8.	Incongruência de Gênero : um estudo comparativo entre os critérios diagnósticos CID-10, CID-11 e DSM-5 *Soll, Bianca Machado Borba January 2016 (has links)*	A presente dissertação tem o objetivo de discutir a proposta dos critérios diagnósticos da CID-11 para Incongruência de Gênero e comparar as diretrizes dos manuais diagnósticos DSM-5 e CID-10 para Disforia de Gênero e Transtorno de Identidade de Gênero, respectivamente. A Organização Mundial da Saúde (OMS) está em processo de revisão da Classificação Internacional de Doenças (CID). Diferentemente do sistema de classificação vigente (CID-10), as modificações propostas pela CID-11 no que diz respeito à condição transexual são norteadas pela compreensão de que esta não é doença mental e que o acesso à saúde desta população necessita ser ampliado. O artigo derivado desta dissertação compara os critérios nos manuais diagnósticos existentes, o DSM-5 e da CID-10, em uma amostra brasileira de pessoas transexuais que procuraram serviços de saúde especificamente para a transição física. Este é um estudo transversal multicêntrico que inclui uma amostra de 103 indivíduos que procuraram os serviços em um dos dois principais centros de referência no Brasil especializados em identidade de gênero. O método da pesquisa consiste na aplicação, por profissionais previamente treinados, de uma entrevista estruturada desenvolvida pelo WHO´s Field Study Coordination Group for ICD-11 Mental and Behavioural Disorders que contempla os critérios diagnósticos. Os resultados revelam que, embora exista desacordo teórico nos critérios há uma sobreposição entre os dois sistemas quanto à confirmação do diagnóstico, com o DSM-5 mais inclusivo. Adicionalmente, a média do tempo de espera para ter acesso a este tipo de serviço é de quase uma década. Nossos achados confirmam a ideia de que há pouco consenso quanto aos critérios diagnósticos dos comportamentos transgêneros, considerando a diversidade de contextos sociais e culturais e que seguem com pouca diferenciação tanto etiológica quanto clínica para fins diagnósticos. / The current work aims to discuss the proposed diagnostic criteria of ICD-11 for Gender Incongruence and compare the diagnostic criteria of DSM-5 and ICD-10 Gender Dysphoria and Gender Identity Disorder, respectively. The World Health Organization (WHO) is reviewing the International Classification of Diseases (ICD). Despite the existing classification system (ICD-10), changes proposed by ICD-11 concerning transgender condition are guided by the understanding that it is not a mental illness and that this population needs health service access to be expanded. The study derived from this work aim to compare the criteria in the existing diagnostic manuals, the DSM-5 and the ICD-10, among a Brazilian sample of transgender persons who sought health services specifically for physical transition. This is a multicenter cross-sectional study that includes a sample of 103 subjects who sought services for gender identity disorder in one of two main reference centers in Brazil. The research method consists of applying a structured interview, which is comprised of the diagnostic criteria from the two manuals. The results reveal that although the theoretical disagreement in the criteria, there is an overlap among the two systems as diagnosis confirmation, to the DSM-5 more inclusive. Additionally, the average waiting time to access this type of service is nearly a decade. Although there is not a consensus concerning such on transgenderism in the diversity of social and cultural contexts, the findings confirm previous impression that despite efforts to determine the diagnostic settings, they follow slightly different as to etiology and different clinical presentations of this condition.	Exclusion	Not an observational study assessing the prevalence of PGD according to ICD-11.
9.	Women-specific mental disorders in DSM-V: are we failing again? *Wittchen, Hans-Ulrich 20 February 2013 (has links)*	Despite a wealth of studies on differences regarding the biobehavioral and social–psychological bases of mental disorders in men and women and repeated calls for increased attention, women-specific issues have so far not been comprehensively addressed in past diagnostic classification systems of mental disorders. There is also increasing evidence that this situation will not change significantly in the upcoming revisions of ICD-11 and DSM-V. This paper explores reasons for this continued failure, highlighting three major barriers: the fragmentation of the field of women's mental health research, lack of emphasis on diagnostic classificatory issues beyond a few selected clinical conditions, and finally, the “current rules of game” used by the current DSM-V Task Forces in the revision process of	Exclusion	Not an observational study assessing the prevalence of PGD according to ICD-11.

	DSM-V. The paper calls for concerted efforts of researchers, clinicians, and other stakeholders within a more coherent and comprehensive framework aiming at broader coverage of women-specific diagnostic classificatory issues in future diagnostic systems		
10. Zařazení žáků s Aspergerovým syndromem do běžné třídy na prvním stupni základní školy / Inclusion of Pupils with Asperger syndrome in primary school *Belhajová, Hana January 2020 (has links)*	This work aims to analyze and describe how the inclusion of pupils with Asperger's syndrome takes place in a regular school and to verify the success of inclusion in practice through qualitative research. The theoretical part deals mainly with the possibilities of inclusion and its benefits in the school system, manifestations, and disorders associated with Asperger's syndrome, but also by defining basic concepts such as autism spectrum disorder, Asperger's syndrome, integration, and inclusion. Furthermore, the diagnosis of this spectrum of disorders according to the world criteria ICD 10 and DSM5 and ICD 11, which will come into force in the Czech Republic in 2022. The theoretical part also discusses the methods of approach and the position of teaching assistant. The empirical part of the thesis contains two case studies and evaluation of the success of the inclusion of pupils in mainstream schools based on qualitative research, in which I apply the knowledge from the theoretical part. The methods that were used for data collection and analysis include observation, interview, and expert documents. The research showed interesting results when most of the information and knowledge from the theoretical part was confirmed KEYWORDS Inclusion, Asperger's syndrome, elementary school, first grade, method...	Exclusion	Not an observational study assessing the prevalence of PGD according to ICD-11.
11. Therapie der Binge-Eating-Störung *Hilbert, Anja 27 September 2022 (has links)*	Die Binge-Eating-Störung (BES), als eigenständige Essstörung erstmals im Diagnostischen und Statistischen Manual psychischer Störungen DSM-5 definiert, ist durch wiederkehrende Essanfälle ohne gewichtskompensatorische Verhaltensweisen gekennzeichnet. Die breitere Definition in der avisierten International Classification of Diseases ICD-11 wird zu Veränderungen in Präsentation und Prävalenz dieser Störung führen. Die BES tritt vor dem<br>Hintergrund einer komplexen, multifaktoriellen Ätiologie auf und geht mit einer erhöhten Essstörungs- und allgemeinen Psychopathologie, psychischen und körperlichen Komorbidität einschließlich Adipositas und verringertem Funktionsniveau einher. Trotz dieser Beeinträchtigungen wird die BES häufig weder diagnostiziert noch behandelt.<br>Evidenzbasierte Therapien für die BES umfassen die Psychotherapie, wobei die Kognitive Verhaltenstherapie das etablierteste Verfahren darstellt, und die strukturierte Selbsthilfebehandlung. Andere Therapien wie die Pharmakotherapie, behaviorale Gewichtsreduktionstherapie und Kombinationstherapien erhielten in den aktuellen evidenzbasierten S3-Essstörungsleitlinien einen geringeren Empfehlungsgrad für spezielle Indikationen.	Exclusion	Not an observational study assessing the prevalence of PGD according to ICD-11.
12. Complex trauma and the influence of emotional regulation and interpersonal problems : a review of Complex-PTSD and an empirical study in a prison setting *Browne, Richard January 2017 (has links)*	Background The effects of prolonged, interpersonal trauma have long been recognised. Such traumatic events can lead to the development of post-traumatic stress disorder (PTSD), but are also associated with a range of other psychological difficulties. The forthcoming ICD-11 has proposed the inclusion of a new diagnostic category to cover such trauma reactions, named complex-PTSD (CPTSD). CPTSD is conceptualised as including the core elements of PTSD with additional difficulties with affect regulation, self-concept, interpersonal relationships. This thesis presents a systematic review of the research into the proposed CPTSD diagnosis. In addition, this thesis investigates the association between difficulties with emotional regulation, interpersonal problems and PTSD symptoms in a group of male prisoners, and a male community sample. Aims This project aims to investigate whether the proposed CPTSD diagnosis accurately describes the difficulties seen following complex trauma, and examines whether it is best to view CPTSD is different from exiting disorders, including PTSD and borderline personality disorder (BPD). In addition, it aims to investigate the association between difficulties with emotional regulation, interpersonal problems and PTSD among men in prison. Methods We systematically assessed and synthesised the available research regarding the proposed ICD-11 CPTSD diagnosis. In the second paper, data regarding PTSD, emotional regulation, and interpersonal problems were collected from HMP Glenochil, a male-only prison in Scotland (n=51), and matched to an existing community data set (n=46). Results The results of the systematic review provide partial support for the factorial validity of CPTSD. In addition, they indicate that CPTSD can be conceptualised as distinct from both PTSD and BPD, and that CPTSD is more closely related to prolonged interpersonal trauma than PTSD. However, there is overlap between PTSD and CPTSD in terms of both symptomology and aetiology. The results also indicate high levels of PTSD among male prisoners. In addition, PTSD was found to be strongly associated difficulties with emotional regulation, but not interpersonal problems, in the forensic sample. In the community sample emotional regulation was a less strong predictor of PTSD symptoms, and both emotional regulation, and interpersonal problems were associated with the severity of PTSD. Conclusions This thesis supports the inclusion of CPTSD as a distinct diagnostic entity. Inclusion of CPTSD may allow survivors a better understanding of the aetiology of their difficulties, and may initiate research into effective ways of working with individuals who have experienced complex-trauma. I addition, they demonstrate the need for trauma-informed prison services, which prioritise the development of emotional regulation strategies in recovery and rehabilitation.	Exclusion	Not an observational study assessing the prevalence of PGD according to ICD-11.

13.	Äldre personers erfarenheter av depressiva tillstånd i ålderdomen: En systematisk litteraturstudie *Pettersson, Joel, Kornhall, Sunna January 2019 (has links)*	Bakgrund: Depressioner hos äldre kan vara svårupptäckta till följd av en diffus symtombild samt då diagnossystemen DSM-5 och ICD-11 inte är anpassade för äldre. Många sjuksköterskor har svårigheter i att korrekt identifiera depressioner hos äldre personer. Mer kunskap och förståelse kring depressiva tillstånd hos äldre kan underlätta för psykiatrisjuksköterskor och andra specialistsjuksköterskor att identifiera dessa tillstånd samt leda till en förbättrad psykiatrisk omvårdnad av äldre personer. Syfte: Syftet var att belysa äldre personers erfarenheter av depressiva tillstånd i ålderdomen. Metod: En systematisk litteraturstudie baserad på nio kvalitativa studier genomfördes med Statens beredning för medicinsk och social utvärderings [SBU] metod som grundstruktur. Syntes av de kvalitativa studiernas resultat genomfördes enligt Howell Major och Savin-Baden. Resultat: Två tredje nivåns tema framkom: Det första tredje nivåns temat Att befinna sig på botten speglade den psykiska och existentiella förtvivlan de äldre befann sig i; en situation präglad av orkeslöshet, hopplöshet, självnedvärdering, skuld, skam, stigma, brist på stöd, ensamhet och alienation. Det andra tredje nivåns temat Att ta sig uppåt genom coping och andras stöd återgav de copingstrategier de äldre upplevde hjälpsamma samt betydelsen av andra människors stöd. Slutsats: Äldre personer som lider av depressiva tillstånd i ålderdomen bär erfarenheter av djup förtvivlan ur flera hänseenden och finner kraft i copingstrategier och stöd från andra. Nyckelord: depressiva tillstånd, erfarenheter, psykiatrisk omvårdnad, systematisk litteraturstudie, äldre / Background: Depressions in the elderly can be difficult to detect due to a diffuse symptomatology and because the diagnostic manuals DSM-5 and ICD-11 are not adapted for the elderly. Many nurses have difficulties in correctly identifying depressions in older people. More knowledge and understanding of depressive conditions in the elderly may facilitate for psychiatric-mental health nurses and other advanced practice nurses to identify these conditions and may conduce to an improved psychiatric-mental health nursing care for the elderly. Aim: To shed light on elderly persons' experiences of depressive conditions in old age. Method: A systematic literature review based on nine qualitative studies was conducted, using Swedish Agency for Health Technology Assessment and Assessment of Social Services [SBU] as basic structure. Synthesis of the qualitative studies was carried out according to Howell Major and Savin-Baden. Result: Two third order themes emerged: The first third order theme, Being at rock bottom, reflected the existential and mental despair the elderly was in; a situation characterized by decrepitude, hopelessness, self-deprecating, guilt, shame, stigma, lack of support, loneliness and alienation. The second third order theme, Moving forward through coping and support from others, portrayed the coping strategies the elderly experienced as helpful and the importance of support from others. Conclusion: Elderly persons suffering from depressive conditions in old age experience different aspects of despair and find their path forward through coping strategies and support from others.	Exclusion	Not an observational study assessing the prevalence of PGD according to ICD-11.
14.	The Study of SNOMED CT Applies to the Medical Facilities / SNOMED CT應用於醫療機構之研究 *Song-yang Huang, 黃頌揚 January 2012*	碩士 / 中臺科技大學 / 健康產業管理研究所 / 100 / SNOMED CT has been used abroad for many years, such as the United States, England and the others countries. It could unify the description of the clinical diagnosis, and help doctors to key some ICD code with its mapping. So, we can call SNOMED CT as the "input code" of electronic medical records, and ICD code be the "output code" of electronic medical records. American Health Information Management Association (AHIMA) will combined ICD-11 and SNOMED CT in 2016. At that time, electronic medical record will integrated to be a new style. Taiwan"s NHI developed to EMR, and SNOMED CT should be the future direction. So the purpose of this study are try to introduce SNOMED CT, by interviewing with relevant experts to realize the possibility of SNOMED CT could be applied in Taiwan's medical institutions.<br>In this study, we used the purposive sampling of one central Taiwan's medical center in depth-interview method. The results showed that SNOMED CT might become an inevitable trend, but Taiwan's hospitals still unfamiliar of SNOMED CT now. So, SNOMED CT seems not to suit of electronic medical record process in this moment. Before apply SNOMED CT, model it need to establish information system the whole premises. And it will also influence the quality and quantity of the manpower structure in medical records department. It may not be able to apply on-line SNOMED CT in short term, but we could not ignore its international medical trend	Exclusion	Not an observational study assessing the prevalence of PGD according to ICD-11.
15.	Psychological predictors of post-traumatic stress disorder in children and adolescents *Memarzia, Jessica January 2018 (has links)*	Background: The experience of trauma in childhood, for a minority of individuals, can lead to chronic and distressing symptoms of post-traumatic stress disorder (PTSD) and other mental health difficulties. Cognitive models of PTSD demonstrate key factors involved in the development of symptoms, however, research evidence regarding the role of different pre- peri- and post-trauma predictors of PTSD in children and adolescents is limited and variable. Furthermore, there is scope to understand predictors of mental health outcomes other than PTSD. With the expected publication of ICD-11 in 2018, further research is also necessary to develop our understanding of the new diagnostic category of 'Complex PTSD' in children and adolescents. Methods: Firstly, a systematic literature review and meta-analysis was conducted, summarising the current evidence regarding the role of peritraumatic psychological risk factors in the development of PTSD. Secondly, empirical analysis of pre-existing data from a longitudinal study of children and adolescents experiencing a single-event trauma was conducted. Multiple linear regression models were used to assess four theory-derived predictive models of mental health outcomes (PTSD, CPTSD, depression and anxiety) of trauma in this sample. Results: Population estimates of effect size were moderate for peritraumatic subjective threat and fear as risk factors for PTSD. Effect size estimates for peritraumatic dissociation were small, and evidence for data-driven processing was limited. The empirical study indicated that a cognitive model of predictors was most powerful in	Exclusion	Not an observational study assessing the prevalence of PGD according to ICD-11.

	predicting the development of all four disorders following trauma, and psychosocial and objective event severity models were weak predictors of mental health outcomes. Conclusions: Cognitive processes occurring during and after trauma may be valuable markers of which individuals may be at risk of developing PTSD, CPTSD, depression or anxiety after trauma. Further research of multiple predictors and outcomes of trauma is required in children and adolescents, particularly related to CPTSD.		
16. The Position of Anxiety Disorders in Structural Models of Mental Disorders *Wittchen, Hans-Ulrich, Beesdo, Katja, Gloster, Andrew T. 23 April 2013*	„Comorbidity" among mental disorders is commonly observed in both clinical and epidemiological samples. The robustness of this observation is rarely questioned; however, what is at issue is its meaning. Is comorbidity „noise" – nuisance covariance that researchers should eliminate by seeking „pure" cases for their studies – or a „signal" – an indication that current diagnostic systems are lacking in parsimony and are not „carving nature at its joints?" (Krueger, p. 921).<br>With these words, Krueger started a discussion on the structure of mental disorders, which suggested that a 3-factor model of common mental disorders existed in the community. These common factors were labeled „anxious-misery," „fear" (constituting facets of a higher-order internalizing factor), and „externalizing." Along with similar evidence from personality research and psychometric explorations and selective evidence from genetic and psychopharmacologic studies, Krueger suggested that this model might not only be phenotypically relevant, but might actually improve our understanding of core processes underlying psychopathology. Since then, this suggestion has become an influential, yet also controversial topic in the scientific community, and has received attention particularly in the context of the current revision process of the Manual of Mental Disorders (Fifth Edition) (DSM-V) and the International Classification of Diseases, 11th Revision (ICD-11).<br>Focusing on anxiety disorders, this article critically discusses the methods and findings of this work, calls into question the model's developmental stability and utility for clinical use and clinical research, and challenges the wide-ranging implications that have been linked to the findings of this type of exploration. This critical appraisal is intended to flag several significant concerns about the method. In particular, the concerns center around the tendency to attach wide-ranging implications (eg, in terms of clinical research, clinical practice, public health, diagnostic nomenclature) to the undoubtedly interesting statistical explorations.	Exclusion	Not an observational study assessing the prevalence of PGD according to ICD-11.
17. Gender dysforie u osob s poruchou autistického spektra / Gender dysphoria in persons with autism spectrum disorder *Lukina, Julie January 2020*	The master thesis copes with gender dysphoria (GD) in persons with autism spectrum disorder (ASD). The quantitative research was chosen to study this topic. The main goal was to determine whether people with mild ASD have a higher prevalence of GD or higher rate of GD compared to the general neurotypical population (NT). The main methods used in this research were: Autism Quotient Questionnaire (AQ-10) and the Gender Identity/Gender Dysphoria Questionnaire in Adolescents and Adults (GIDYQ-AA), which made it possible to determine the degree of GD and the potential co-occurrence of a diagnosis of gender incongruence (GI; according to ICD-11). In the research there were 196 subjects with the age ranging from 16 to 74 years. The ASD group was compromised 90 persons and in the NT group there were 106 people. While in the NT group no one admitted transgender identity, in the ASD group 2.2 % of subjects informed about transgender identity and 14.4 % of the autistic group were not sure about it. Based on the GIDYQ-AA results, 4.4 % of people with ASD had a low score indicating a possible presence of a diagnosis of GD (GI). Furthermore, the research found that women with autism had significantly higher rate of GD than both women in the NT group and men with ASD. Overall, subjects with ASD achieved...	Exclusion	Not an observational study assessing the prevalence of PGD according to ICD-11.
18. Unga individers upplevelser av hur gaming påverkar deras hälsa : En litteraturöversikt *Fluur, Kajsa, Kaveh, Maria January 2023*	Gaming, eller datorspelande, är en global hobby som växer sig allt starkare bland ungdomar och unga vuxna. År 2013 ingick Internet gaming disorder som en egen benämning i American Psychiatric Associations diagnosmanual DSM-5 och även WHO har valt att inkludera Gaming disorder i ICD-11. Gaming kan ses som en oskyldig hobby men forskning tyder på att det även kan övergå till ett beroendeliknande tillstånd med flertalet negativa konsekvenser. Syftet med denna kandidatuppsats var att belysa unga individers upplevelser av hur gaming påverkar deras hälsa. Vald metod för att svara på syftet var att utföra en litteraturöversikt med ett strukturerat analysförfarande där både kvantitativa och kvalitativa artiklar analyserades. Litteraturöversikten utgick från ett induktivt förhållningssätt. Resultatet visade på att individer upplevde att det fanns både positiva och negativa aspekter av gaming. Delvis så upplevde de att spelandet gav dem ett syfte och var ett sätt att skapa sociala sammanhang på. Samtidigt upplevde vissa att nära relationer kunde påverkas negativt av spelandet och somliga menade även att det fanns ett samband mellan psykisk ohälsa och gaming. Under diskussionen fördjupar sig och reflekterar författarna till denna litteraturöversikt kring vald metod och resultatets huvudfynd i relation till etik och hållbar utveckling.<br><br>Gaming is a global hobby that is growing in popularity among adolescents and young adults. In 2013, Internet gaming disorder was included as a separate term in the American Psychiatric Association's diagnostic manual DSM-5 and the WHO has also chosen to include gaming disorder in ICD-11. Gaming can be seen as an innocent hobby, but research suggests that it can also turn into an addiction-like	Exclusion	Not an observational study assessing the prevalence of PGD according to ICD-11.

	condition with several negative consequences. The purpose of this bachelor thesis was to highlight young individuals' experiences of how gaming affects their health. The chosen method to answer the purpose was to perform a literature review with a structured analysis procedure where both quantitative and qualitative articles were analyzed. The literature review was based on an inductive approach. The results showed that individuals felt that there were both positive and negative aspects of gaming. In part, they felt that gaming gave them a purpose and was a way to create social connections. At the same time, some felt that close relationships could be negatively affected by gaming and some also felt that there was a connection between mental illness and gaming. During the discussion, the authors of this literature review delve into and reflect on the chosen method and the main findings in relation to ethics and sustainable development. Translated with DeepL.com (free version)		
19. Autism spectrum disorders:an epidemiological and clinical study Mattila, M.-L. (Marja-Leena) 21 January 2014	Background: Autism spectrum disorders (ASDs), defined as pervasive developmental disorders (PDDs) in DSM-IV and ICD-10, become manifest in childhood, ranging from a severe form, autism, to milder forms, Asperger syndrome (AS) and pervasive developmental disorder not otherwise specified (PDD-NOS)/atypical autism. AS is also specified in criteria by Gillberg and by Szatmari et al. Different diagnostic criteria sets, overlaps, inaccuracies and exclusion criteria of many comorbid psychiatric disorders in PDDs have caused confusion. In DSM-5, PDDs were replaced by one diagnosis called ASD.<br>Aims and methods: This is an epidemiological study of 8-year-old children and a clinical study of 9- to 16-year-old high-functioning outpatients with AS/autism (HFA). The epidemiological target population (n = 4,422) was rated via the Autism Spectrum Screening Questionnaire (ASSQ) by parents and/or teachers and a screened sample was examined in order to estimate the prevalence of ASDs, to discover deficits in the diagnostic criteria of ASDs, to evaluate DSM-5 draft criteria for ASD, and to assess cut-off scores for the Finnish ASSQ. Comorbid psychiatric disorders were identified and overall level of functioning rated in 50 subjects with AS/HFA.<br>Results: The prevalence of AS according to DSM-IV was 2.5, to ICD-10 2.9, to Gillberg 2.7, and to Szatmari et al. 1.6 per 1,000. The prevalence of autism was 4.1 and that of ASDs 8.4 per 1,000 (DSM-IV). DSM-5 draft criteria were less sensitive in detecting AS/HFA. For 7- to 12-year-old children (IQ ≥ 50), the optimal cut-off scores were 30 in clinical settings and 28 in total population screening using summed parent-rated and teacher-rated ASSQ scores. Comorbid psychiatric disorders were common (prevalence 74%) and often multiple; behavioral disorders in 44%, anxiety disorders in 42%, and tic disorders in 26%. Oppositional defiant disorder, depressive disorder and anxiety disorders as comorbidities indicated significantly lower levels of functioning.<br>Conclusions: Our results indicate the need to standardize the diagnostic criteria. The ICD-11 criteria should be uniform and harmonize with DSM-5. Determining cut-off scores for ASD screening instruments in different languages and cultures is of utmost importance. Clinicians are reminded to investigate psychiatric comorbidity in ASDs in order to target treatment and rehabilitation precisely. / Tiivistelmä	Exclusion	Not an observational study assessing the prevalence of PGD according to ICD-11.
20. Cultural Encounters in Medicine: (Re)Constituting Traditional Medicine in Taiwan under Colonization, Modernity, and Exchange Tsai, Hung-Yin 04 August 2021	Today we have many alternative medicines, not a few of which connect back to aboriginal cultures. Some of these alternative medicines were born under the influence of European imperialism, as they were not "alternative" until modern empires and modern medicine came to these distant regions. The present study begins with a broad question: how did conceptions of the relationship between modern Western medicine and traditional local non-Western medicine come to be? To explore the historical origins of these two conceptions, I focus herein on Japanese colonial Taiwan (1895–1945), where modern medicine became dominant while traditional medicine also flourished. My research finds that the historical realities of colonial Taiwan were not reflected in the progressive narrative of medicine. According to this narrative, modern medicine became dominant around the world while traditional medicines were swept into the ash heap of history because only modern medicine was the true, effective science of preventing, diagnosing, and treating physical ailments. The history of colonial Taiwan teaches us a much different lesson: practitioners of traditional medicine there were a significant part of the public health system during the colonial period. For example, they rallied against the plague in the late 19th century, diagnosing and treating patients when antibiotics had yet to be developed. Even so, the island witnessed an institutional medical shift, in which licensed practitioners of modern medicine deified modern medicine and denigrated traditional medicine, labeling the latter "primitive" and "non-medicine." In response, practitioners of traditional medicine produced new narratives aiming to challenge this colonial boundary between medicine and non-medicine. These practitioners' fundamental argument was that traditional medicine, though epistemologically different from modern medicine, was still legitimate medicine. From this effort, we now have the widely held belief today that both modern medicine and traditional medicine are legitimate, but distinct, medicines. This historical outcome of colonial resistance occurred worldwide. In my study, I identify the social, political, and colonial contexts of medical resistance in Japanese Taiwan, revealing their roots in issues related to inequality, distrust, economic affordability, and conceptions of body and health care. / Doctor of Philosophy / In this study, I explore conceptions of modern and traditional medicine through a historical lens, and break down two related myths: the first myth is the progressive narrative of modern medicine, which holds that modern medicine became dominant because of its medical	Exclusion	Not an observational study assessing the prevalence of PGD according to ICD-11.

	superiority; and the second myth is the narrative held by extremist supporters of traditional medicine, who insist that only millennia-old traditional medicine can resolve human ailments without giving rise to untoward side effects and exorbitant costs. I show that, in the case of Japanese colonial Taiwan (1895–1945), both modern and traditional medicine flourished. The history of colonial Taiwan shows us that modern medicine on the island became dominant for two main reasons: first, the public health system successfully dealt with epidemics, which were the most significant threat to life at that time; and second, the colonial government recognized only modern medicine and labeled traditional medicine a non-medicine despite relying on its practitioners in the pre-antibiotic age. The history of colonial Taiwan also shows us that traditional medicine is not "old wisdom" unchanged for thousands of years. Beginning in the 19th century, practitioners of Taiwanese traditional medicine re-constituted it for colonial consumption, arguing that traditional medicine was also true medicine, though epistemologically distinct from modern medicine. This conception of traditional medicine has since informed many current views of traditional medicine. In 2018, the World Health Organization (WHO) published the eleventh revision of the International Classification of Diseases (ICD-11), which, for the first time, featured a chapter on traditional Chinese medicine covering such topics as diagnostic techniques for Qi, blood, and fluid disorders. This inclusion of traditional medicine into the ICD-11 is a major step forward in this process of medical integration and may help resolve the historical confrontation between modern and traditional medicine. However, the WHO decision limits recognition of traditional medicine to Chinese medicine, excluding all other kinds of traditional medicine. Thus, the historical question of whether or not traditional medicine is a true medicine remains ultimately unanswered.		
21. *APPLYING EVIDENCE MAPPING METHODOLOGIES TO THE WORLD HEALTH ORGANIZATION'S TUBERCULOSIS GUIDELINES* *Hajizadeh, Anisa January 2020*	Background: Tuberculosis (TB) is the number one infectious disease killer in the world. TB is both preventable, and curable. Since 1997, the World Health Organization's (WHO) Global TB (GTB) Programme has released evidence-informed publications to guide member states. In their EndTB strategy, the WHO set a mandate to eradicate TB by 2035, in part by intensifying TB research and innovation. As an effort towards this goal, this project applies evidence mapping methodologies to published WHO TB recommendations, in an innovative process called "recommendation mapping" (RM).<br>Objectives: The prime objective of RM is to allow guideline developers and key stakeholders to identify gaps and clusters of recommendations across publications, serve as an instrumental tool in the sequence of guideline development (from intelligent priority setting, to the assembly of final recommendations) and increase the accessibility of key guideline components. The secondary objective of this work is to poise guideline components for live update and refinement in a rapidly learning health system.<br>Methods: In this mixed methods study, a methodological framework for mapping guideline components is proposed, with both a quantitative and narrative assessment of raw data and final map outputs. A qualitative analysis from the perspective of key stakeholders, policy-makers, researchers and WHO-GTB liaisons working in guideline development is also included. For the methodological piece, all publications containing WHO TB recommendations were eligible for the mapping exercise. Each recommendation was extracted according to all subdomains of their PICO backbone. Subsections of recommendations are coded using existing ontologies (SNOMED-CT, ATC, ICD-11). A centralized database containing extracted and coded recommendations was then presented in an online and interactive schematic. For the qualitative assessment of palatability of this approach within the organization, semi-structured interviews and a survey was delivered to eligible participants at two Guideline Development Group meetings for WHO tuberculosis treatment and screening guidelines.<br>Results: The notable result of this work is the development, refinement and application of recommendation mapping methodologies. 20 WHO-GTB guidelines underwent an application of the novel recommendation methodologies proposed in this thesis to create an interactive map, and a searchable database. In-depth interviews and survey results with 21 participants (WHO GTB staff, WHO TB- guideline development group members and technical experts) pointed to concerns in the current accessibility and organization of WHO-GTB guidelines.<br>Conclusions: Recommendation mapping may have utility in charting the terrain of recommendations, inform priority setting, and provide a scaffold for the future transition to living guidelines. / Thesis / Master of Public Health (MPH) / The World Health Organization (WHO) issues guidelines to help clinicians, policy-makers, and researchers make informed decisions in their work. Guidelines contain recommendations that can be thought of as bottom-line answers to the questions we ask the scientific literature (based on the evidence available to us today). The WHO's Tuberculosis (TB) Department is partaking in a novel digital reorganization of their guideline recommendations using the evidence-mapping methods proposed in this thesis. This thesis uses the principles of evidence	Exclusion	Not an observational study assessing the prevalence of PGD according to ICD-11.

	mapping to create recommendation maps that, like any map, chart the landscape in a given domain (in this case, TB recommendations). The recommendation map will help guide the WHO in setting priorities for future research and guideline development.		
22. *A espiritualidade na obra de autores da psicologia, saúde e educação Lousada, Márcia Gouvêa 22 September 2017*	The objective of this research was to understand the concept of "spirituality" in the bibliographic productions of the main authors of the areas of Psychology, Health and Education, from 2010 to 2015, trying to identify their research objectives, the definitions assigned to the term, the relationships established with other concepts and, finally, the contributions they offer. In order to achieve this objective, a bibliographic research was carried out on the state of knowledge about scientific journals (related to Anpepp, Abrasco and Nupes) and the thesis portal of Capes, and an integrative review was used as a methodology to carry out such mapping. The results of the research seek to synthesize and order the conjunctures that presented themselves in the researched material and, with regard to the definition of spirituality, it was clear that none of the three areas investigated uses a single homogeneous concept, which confers an impression of intangibility and immateriality to the concept. It was observed that the theme is presented in a beneficial and useful way, always associated with improvement in self-concept, search of autonomy, better health and quality of life, self-realization, search for the encounter with the meaning of life that is found in the experience with the Sacred. The results indicate a need for paradigm revision, due to the epistemic place that spirituality should occupy, as well as a more complex and broad conception of human being, which embraces spirituality as an inherent and constitutive aspect of the active subject in the care of him/herself. The imminence of more studies on the subject and the need to include it in professional training courses are also present, especially because of the possibility of signifying advances and generating new fields of work in the three areas. The authors researched in the area of psychology understand "spirituality" as an object of psychology, since it impacts subjectivity, suggesting a "Psychology of Spirituality". The articles of the authors of the area of health understand Spirituality as part of practices accepted recently in public health programs and recommended the inclusion of a text on spirituality in the International Code of Diseases – 11th Revision (ICD-11). In Education, the abstracts of selected theses indicated a greater concern in a more humane education, believing that "spirituality" is the foundation of the educational practice, and may help in the task of educating a more cohesive, whole and complete human being and postulate that the teacher would need to experience spirituality in his process of self-education. In conclusion, the texts of the authors of the three areas of knowledge point to experiences that have resulted in an increasing of self-esteem and self-confidence, including reports in the area of education and psychology that have indicated journeys and experiences that enabled the transformation of subjectivity and resignification of self, showing that the concept of spirituality would need to be considered a dimension of the human being and proposing the conception of a subject that transcends the biological, psychological and social dimensions	Exclusion	Not an observational study assessing the prevalence of PGD according to ICD-11.
23. *Global mortality attributable to alcoholic cardiomyopathy Manthey, Johann Jakob 04 September 2020*	Introduction:<br>Globally, around 2.6 billion people have consumed alcohol in 2017. In the same year, nearly 3 million or 5% of all deaths were attributable to alcohol consumption, the majority of which were non-communicable diseases, such as cancer, digestive and cardiovascular diseases. Chronic heavy alcohol consumption in particular causes harm to the cardiovascular system and is linked to an elevated risk on the occurrence of ischemic heart diseases and cardiomyopathies. The latter constitutes a heterogeneous group of cardiovascular diseases, which can generally be characterized by a weakened heart muscle. The causal link between chronic heavy alcohol consumption and	Exclusion	Not an observational study assessing the prevalence of PGD according to ICD-11.

	cardiomyopathy has long been recognized, with the Tenth Revision of the International Classification of Diseases (ICD-10) listing alcoholic cardiomyopathy (ACM) as a fully alcohol-attributable diagnosis. For a few, predominately high-income countries, civil registries provide valuable information of ACM mortality. However, for the majority of countries and global population, the cardiomyopathy burden attributable to alcohol consumption needs to estimated. Established methods for estimating alcohol-attributable fractions (AAF), i.e. proportion of an outcome which could be avoided in a scenario of zero alcohol consumption, could not be applied for cardiomyopathy as the link between alcohol consumption levels and risk of cardiomyopathy could not be specified. Accordingly, a global assessment of the contribution of alcohol consumption to the disease burden from cardiomyopathy was lacking.<br><br>Aims and objectives:<br>First, to develop methods for estimating the contribution of alcohol consumption to cardiomyopathy that can be used globally (study I). Second, to apply the method developed in study I to estimate the global mortality from ACM (study II). Third, to assess differences between this method and an alternative method for estimating the contribution of alcohol consumption to cardiomyopathy proposed during pursuit of these aims (study III).<br><br>Design:<br>Statistical modelling study with country-level data as unit of analyses.<br>Study I. Based on mortality data from civil registries, the proportion of deaths from ACM among deaths from any cardiomyopathy (=AAF) was used as proxy for the link between alcohol consumption and cardiomyopathy. To generalize this link to countries without available civil registry data, associations of population alcohol exposure and registered AAF were established. Cardiomyopathy deaths that are attributable to alcohol use were quantified in those countries with available registry data.<br>Study II. For countries without available civil registry data, ACM mortality was estimated using population alcohol exposure data based on the methods from study I. As a result, national, regional and global estimates of the mortality attributable to ACM were obtained for the year 2015.<br>Study III. In the alternative method developed by the Global Burden of Disease (GBD) study team, the contribution of alcohol consumption to cardiomyopathy was estimated taking into account that actual ACM deaths may be incorrectly coded as so-called garbage codes (disease codes that do not accurately describe the underlying cause of death). In the alternative method, garbage codes were redistributed to both cardiomyopathy and ACM using statistical procedures. The underlying assumptions for the redistribution of garbage codes were examined by comparing registered and estimated ACM mortality data taking into account the distribution of alcohol exposure.<br><br>Data sources:<br>Data on population alcohol exposure (alcohol per capita consumption, prevalence of heavy episodic drinking, prevalence of alcohol use disorders) were sourced from publicly available World Health Organization (WHO) data bases. As outcome data, sex-specific mortality counts from different disease groups (ACM, any cardiomyopathy, and selected garbage codes) were obtained at the country level from three different sources: First, WHO mortality data base, which provide civil registry mortality data on nearly half of all member states, coded according to the ICD-10. Second and third, ‘Global Health Estimates’ and ‘GBD Results Tool’ data bases, which provide complete and consistent mortality estimates aggregated into larger disease groups for all WHO member states. Data on covariates were obtained from the United Nations and the World Bank.<br><br>Statistical analyses:<br>In study I, the dependent variable – AAF for cardiomyopathy – was calculated by dividing deaths from ACM by deaths from any cardiomyopathy, based on civil registry data from N=52 countries. Taking into account country-specific crude mortality rates of ACM, AAF were modeled in two-step sex-specific regression analyses using population alcohol exposure as covariate. AAF were estimated for the same set of N=52 countries, in addition to N=43 countries without civil registry data. Estimated AAF were compared to registered AAF available for N=52 countries.<br>In study II, the global mortality of ACM was estimated by combining civil registry ACM mortality data for N=91 countries and estimated ACM mortality for N=99 countries without available civil registry data. For the latter set of countries, ACM mortality data were calculated by estimating AAF based on the methodology outlined in the first study and subsequently applied to all cardiomyopathy deaths. As a proxy for under-reporting of ACM in civil registries, estimated ACM deaths were compared to registered ACM deaths for N=91 countries.		

	In study III, ACM mortality estimates from the GBD study were compared against registered ACM mortality data for N=77 countries, aiming to test underlying assumptions for redistribution of garbage-coded deaths in the alternative method. For this purpose, descriptive statistics and Pearson correlations were used to assess the association of estimated and registered deaths and to examine consistency of estimates with population alcohol exposure.<br><br>Results:<br>In study I, population alcohol exposure and ACM mortality were closely linked (spearman correlation=0.7), supporting the proposed modelling strategy. For N=95 countries, the AAF for cardiomyopathy was estimated at 6.9% (95% confidence interval (CI): 5.4-8.4%), indicating that one in 14 of all cardiomyopathy deaths were attributable to alcohol in the year 2013 or the last available year. The findings were robust, with 78% of all estimated AAF deviating less than 5% from registered AAF.<br>In study II, it was estimated that 25,997 (95% CI: 17,385-49,096) persons died from ACM in 2015 globally, with 76.0% of ACM deaths being located in Russia. Globally, 6.3% (95% CI: 4.2-11.9%) of all deaths from cardiomyopathy were estimated to be caused by alcohol. Furthermore, indications of underreporting in civil registration mortality data were found, with two out of three global ACM deaths being possibly misclassified.<br>In study III, findings suggested that only one in six ACM deaths were correctly coded in civil registries of N=77 countries. However, the algorithm accounting for misclassifications in the GBD study was not aligned with population alcohol exposure, which has led to implausibly high ACM mortality estimates for people aged 65 years or older. Specifically, registered and estimated ACM mortality rates diverged in the elderly, which was corroborated with decreasing correlations in these age groups.		
24. Från sorg till professionellt samtalsstöd i sorgearbetet efter en närståendes död : - En kvalitativ studie utifrån fem sörjandes upplevelser. / From grief to use of professional therapeutic conversation in mourning the death of a loved one : - A qualitative study of the experiences of five bereaved. Elfström, Teresa January 2012	The purpose of the study was to investigate why, how and with what results some bereaved people seek professional counseling support after the death of a loved one. A qualitative descriptive study comprising semi-structured interviews with five bereaved people was chosen as the research design. The data material was analyzed according to Theme Analysis. The results of the study indicate that the participants have experienced that their grief and need for a longer sick leave in the mourning process has not been taken seriously by either doctors, the insurance fund or employers, which has caused them unnecessary stress that has impaired the mourning process. Counseling support in public health care at hospitals, emergency rooms and health care centers has been perceived as unsatisfactory due to the staff's lack of ability to meet, inform and support mourners. It has also been felt that treatment methods are not adapted to patients' specific needs for help but rather to political demands for profitability and efficiency. The CBT treatment offered is not perceived to be suitable for processing complicated grief. Instead, mourners demand psychodynamic crisis/grief therapy individually and/or in groups, which they have experienced positively from private care providers, but which is considered too expensive. The Church of Sweden's free grief groups have also been perceived positively, but non-believers and those of other faiths are reluctant to attend. There is a strong need for improvement in the professional counseling support currently offered to bereaved people in Sweden. It would be desirable that<br>Translated with DeepL.com (free version)	Exclusion	Not an observational study assessing the prevalence of PGD according to ICD-11.

Name of resource	URL	Date searched	Searcher	Pathway followed (Browsed Headings and Terms searched on the respective sites within the website)
Science.gov	https://www.science.gov/	04/13/2024	KN	• We searched for "International Classification of Disease* 11" or ICD-11 or "ICD 11" or ICD11 OR "International Statistical Classification of Disease* 11" or ICD-Eleven or " ICD Eleven" within the title of Science.gov. entries.<br>• The search retrieves 100 top results from 2468 found in all sources which are displayed.<br>• Based on the Title of the document relevance was assessed and inclusion for the full-text-screening considered.<br>• We clicked on the following entries to further evaluate relevance:<br>▪ N01CO72400-11<br>▪ Microsoft Word - CTPM Executive Summary 7-1-11 _2_.doc: Colon and Rectal Cancers Clinical Trials Planning Meeting Executive Summary<br>▪ 11<br>▪ M6-Ch.11: Involving Diverse Community Organizations in Tobacco Control Activities<br>▪ Breves de HINTS Numero 11: Spanish pamphlet<br>▪ CCR-11-1105 6634..6640: Publication entitled " Adaptive Clinical Trial Designs for Simultaneous Testing of Matched Diagnostics and Therapeutics"<br>• No relevant entries were identified through Science.gov.

Table 3: Documentation of Science.gov search

Supplement C

Data Extraction Form

1. Bibliographic information
    a. First author:
    b. Publication year:
    c. Title:

2. Inclusion/Exclusion:
3. Reason for Exclusion:
4. Assessment years:
    a. Year of assessment (beginning)
    b. Year of assessment (end)
5. Location:
    a. Location of data collection
    b. Location of affiliated institution
6. Study Details:
    a. Study type
    b. Setting:
        i. Target population
        ii. Was the study population described as representative?
        iii. Recruitment process
        iv. Survey type
7. Eligibility Criteria:
    a. Bereavement or specification of type of bereavement
    b. Time since bereavement
    c. Elevated grief symptoms
    d. Language or writing reading level/proficiency
    e. Age criterion
    f. Citizenship
    g. Residence
    h. Nature of death
    i. Health status
    j. Other
8. Sample details:
    a. Sample size
    b. Portion of female study participants in percent
    c. Portion of male study participants in percent
    d. Portion of diverse/gender non-conforming participants in percent
    e. Mean age
    f. Standard deviation of age
    g. Educational level of study participants
    h. Mean time in months since death at time of assessment
    i. Relationship to the deceased ("deceased is")
    j. Portion of study participants bereaved through a violent death/unnatural, non-violent/natural death in percent
9. Instruments used for assessments
10. Diagnostic algorithm
    a. Which diagnostic criteria were applied?
    b. Which ICD-11 criteria were fulfilled due to the eligibility criteria or assessment/analysis process?
    c. Further relevant specification of diagnostic algorithm
    d. Do the applied criteria represent a raised diagnostic threshold for ICD-11?
    e. Do the applied criteria represent a lowered diagnostic threshold for ICD-11?
    f. Which ICD-11 criteria were omitted?
11. Estimates

a. Prevalence estimates
b. Prevalence estimates for dual time points

Supplement D

Table 1: Documentation of contact attempts

	**Publication**	**Access**	**Contact attempt**	**Result**
1	Aoun SM, Kissane DW, Cafarella PA, Rumbold B, Hogden A, Jiang L, Bear N. Grief, depression, and anxiety in bereaved caregivers of people with motor neurone disease: a population-based national study. Amyotroph Lateral Scler Frontotemporal Degener. 2020 Nov;21(7-8):593-605. doi: 10.1080/21678421.2020.1790610. Epub 2020 Jul 16. PMID: 32668960.	Only accessible through "Taylor & Francis Online". As the Charité provides no institutional access, I am unable to consider the publication.	An email asking for the publication was sent to Prof. Aoun on the 13th of April 2024.	The publication was kindly provided by Prof. Aoun on the 14th of April 2024.
2	Bonanno GA, Malgaroli M. Trajectories of grief: Comparing symptoms from the DSM-5 and ICD-11 diagnoses. Depress Anxiety. 2020 Jan;37(1):17-25. doi: 10.1002/da.22902. Epub 2019 Apr 22. PMID: 31012187.	Only accessible through "Wiley Online Library". As the Charité provides no institutional access, I am unable to consider the publication.	An email asking for the publication was sent to Dr. Malgaroli on the 13th of April 2024.	The publication was kindly provided by Dr. Malgaroli on the 13th of April 2024.
3	Burns E, Prigerson HG, Quinn SJ, Abernethy AP, Currow DC. Moving on: Factors associated with caregivers' bereavement adjustment using a random population-based face-to-face survey. Palliat Med. 2018 Jan;32(1):257-267. doi: 10.1177/0269216317717370. Epub 2017 Jun 19. PMID: 28627971.	Only accessible through "Sage Journals". As the Charité provides no institutional access, I am unable to consider the publication.	An email asking for the publication was sent to Prof. Currow on the 13th of April 2024.	The publication was kindly provided by Prof. Currow on the 15th of April 2024.
4	Haneveld J, Rosner R, Vogel A, Kersting A, Rief W, Steil R, Comtesse H. Same name, same content? Evaluation of DSM-5-TR and ICD-11 prolonged grief criteria. J Consult Clin Psychol. 2022 Apr;90(4):303-313. doi: 10.1037/ccp0000720. PMID: 35446077.	Only accessible through "Sage Journals". As the Charité provides no institutional access, I am unable to consider the publication.	An email asking for the publication was sent to Dr. Haneveld on the 13th of April 2024.	The publication was kindly provided by Dr. Haneveld on the 13th of April 2024.
5	Kokou-Kpolou CK, Lenferink LIM, Brunnet AE, Park S, Megalakaki O, Boelen P, Cénat JM. The ICD-11 and DSM-5-TR prolonged grief criteria: Validation of the Traumatic Grief Inventory-Self Report Plus using exploratory factor analysis and item response theory. Clin Psychol Psychother. 2022 Nov;29(6):1950-1962. doi: 10.1002/cpp.2765. Epub 2022 Jul 20. PMID: 35751156.	Only accessible through "Wiley Online Library". As the Charité provides no institutional access, I am unable to consider the publication.	An email asking for the publication was sent to Dr. Kokou-Kpolou on the 14th of April 2024.	No response was received by the 2nd of July 2024.
6	Lavenda O, Mahat-Shamir M, Lorenz L, Hamama-Raz Y, Greenblatt-Kimron L, Pitcho-Prelorentzos S, Ring L, Bar-Shua E, Ben-Ezra M. Revalidation of Adjustment Disorder-New Module-4 screening of adjustment disorder in a non-clinical sample: Psychometric reevaluation and correlates with other ICD-11 mental disorders. Psych J. 2019 Sep;8(3):378-385. doi: 10.1002/pchj.286. Epub 2019 Apr 15. PMID: 30983131.	Only accessible through "Wiley Online Library". As the Charité provides no institutional access, I am unable to consider the publication.	An email asking for the publication was sent to Prof. Ben-Ezra on the 14th of April 2024.	The publication was kindly provided by Prof. Ben-Ezra on the 15th of April 2024.
7	Singer J, McLean E, Kahler J, Papa A. An evaluation of common risk factors for prolonged grief disorder using the international classification of diseases-11 criteria. Aging Ment Health. 2022 Nov;26(11):2202-2207. doi: 10.1080/13607863.2021.1998359. Epub 2021 Nov 5. PMID: 34738488.	As the Charité provides no institutional access to "Taylor & Francis Online", I am unable to consider the publication.	An email asking for the publication was sent to Dr. Singer on the 26th of April 2024.	The publication was kindly provided by Dr. Singer on the 26th of April 2024.
8	Song C, Fu Z, Li W, Yu Y, Ma J, Li X, Wang J. Social support and prolonged grief disorder symptoms in parents who lost their only child from urban and rural China. Psychol Trauma. 2023 Feb;15(2):304-312. doi: 10.1037/tra0001116. Epub 2021 Aug 26. PMID: 34435813.	As the Charité provides no institutional access to "Taylor & Francis Online", I am unable to consider the publication.	An email asking for the publication was sent to Dr. Zhongfang Fu on the 15th of April 2024.	No response was received by the 2nd of July 2024.

Supplement E

Table 1: Publications excluded during full-text screening due to non-fulfillment of eligibility criteria

	Author, Publication Year	Title	Reason for Exclusion
1.	Boelen, 2022 [1]	Prolonged grief disorder in DSM-5-TR: Early predictors and longitudinal measurement invariance	No prevalence of ICD-11 PGD was reported.
2.	Burns, 2018 [2]	Moving on: Factors associated with caregivers' bereavement adjustment using a random population-based face-to-face survey	No prevalence of ICD-11 PGD was reported.
3.	Chen, 2021 [3]	Profiles of grief, post-traumatic stress, and post-traumatic growth among people bereaved due to COVID-19	No prevalence of ICD-11 PGD was reported.
4.	Eisma, 2020 [4]	ICD-11 Prolonged Grief Disorder Criteria: Turning Challenges Into Opportunities With Multiverse Analyses	Opinion article
5.	Harrison, 2022 [5]	Inclusion of the other in the self as a potential risk factor for prolonged grief disorder: A comparison of patients with matched bereaved healthy controls	No prevalence of ICD-11 PGD was reported.
6.	Harrison, 2021 [6]	Mental Imagery in Patients with Prolonged Grief Disorder: a Comparison with Matched Bereaved Healthy Controls	No prevalence of ICD-11 PGD was reported.
7.	Karatzias, 2022 [7]	The Network Structure of ICD-11 Disorders Specifically Associated with Stress: Adjustment Disorder, Prolonged Grief Disorder, Posttraumatic Stress Disorder, and Complex Posttraumatic Stress Disorder	No prevalence of ICD-11 PGD was reported.
8.	Kokou-Kpolou, 2022 [8]	The ICD-11 and DSM-5-TR prolonged grief criteria: Validation of the Traumatic Grief Inventory-Self Report Plus using exploratory factor analysis and item response theory	Not available during full-text screening.
9.	Lavenda, 2019 [9]	Revalidation of Adjustment Disorder-New Module-4 screening of adjustment disorder in a non-clinical sample: Psychometric reevaluation and correlates with other ICD-11 mental disorders	No prevalence of ICD-11 PGD was reported.
10.	O'Connor, 2023 [10]	Development and validation of the Aarhus PGD scale for operationalizing ICD-11 and DSM-5-TR TR Prolonged Grief Disorder	No prevalence of ICD-11 PGD was reported.
11.	Prigerson, 2017 [11]	Rebuilding Consensus on Valid Criteria for Disordered Grief	Opinion article
12.	Shear, 2014 [12]	Testing sensitivity of different criteria for complicated grief	Lack of information regarding the diagnostic algorithm.
13.	Smith, 2022 [13]	Psychometric Characteristics of the Oxford Grief Memory Characteristics Scale and Its Relationship With Symptoms of ICD-11 and DSM-5-TR Prolonged Grief Disorder	No prevalence of ICD-11 PGD was reported.
14.	Song, 2023 [14]	Social support and prolonged grief disorder symptoms in parents who lost their only child from urban and rural China	Not available during full-text screening.
15.	Song, 2021 [15]	Loss of a co-twin at birth and subsequent risk of psychiatric disorders	No prevalence of ICD-11 PGD was reported.
16.	Stelzer, 2020 [16]	Cross-cultural generalizability of the ICD-11 PGD symptom network: Identification of central symptoms and culturally specific items across German-speaking and Chinese bereaved	No prevalence of ICD-11 PGD was reported.
17.	Tay, 2016 [17]	Factorial structure of complicated grief: associations with loss-related traumatic events and psychosocial impacts of mass conflict amongst West Papuan refugees	No prevalence of ICD-11 PGD was reported.
18.	Xu, 2022 [18]	Network analysis of PGD, PTSD and insomnia symptoms in Chinese shidu parents with PGD	No prevalence of ICD-11 PGD was reported.

Table 2: Publications excluded as likely duplicates of other included studies

	Author, Publication Year	Title	Reason for Exclusion
1.	Comtesse, 2020 [19]	When does grief become pathological? Evaluation of the ICD-11 diagnostic proposal for prolonged grief in a treatment-seeking sample	Excluded as it likely originates from the same project as Haneveld, 2022. In an effort to avoid the duplication of prevalence data.
2.	Hennemann, 2023 [20]	Somatic symptom distress and ICD-11 prolonged grief in a large intercultural sample	Excluded as it is a likely originates from the same project as Killikelly, 2023. In an effort to avoid the duplication of prevalence data.
3.	Lundorff, 2020 [21]	Are there gender differences in prolonged grief trajectories?	Excluded as it is a likely originates from the same project as Lundorff, 2021. In an effort to avoid the duplication of prevalence data.
4.	Shevlin, Redican, 2023 [22]	Symptoms and levels of ICD-11 Prolonged Grief Disorder in a representative community sample of UK adults	Excluded as it is a likely originates from the same project as Shevlin, 2023. In an effort to avoid the duplication of prevalence data.
5.	Vang, 2022 [23]	Do we all grieve the same? A multigroup test of the dimensional structure of	Excluded as it is a likely

		prolonged grief disorder among Danish bereaved partners and children	originates from the same project as Lundorff, 2021. In an effort to avoid the duplication of prevalence data.

Table 3: Study populations excluded as likely duplicates of other included studies

	Author, Publication Year	Title	Reason for Exclusion
1.	Lenferink, 2022 [24]	Valid measurement of DSM-5 persistent complex bereavement disorder and DSM-5-TR and ICD-11 prolonged grief disorder: The Traumatic Grief Inventory-Self Report Plus (TGI-SR+). Compr Psychiatry.	The "community sample of Dutch adults bereaved of a family member or other close person due to various causes" originates from the same project as Eisma, 2023. In an effort to avoid the duplication of prevalence data.

References

1. Boelen PA, Lenferink LI. Prolonged grief disorder in DSM-5-TR: Early predictors and longitudinal measurement invariance. Aust N Z J Psychiatry. 2022 Jun;56(6):667–74.

2. Burns E, Prigerson HG, Quinn SJ, Abernethy AP, Currow DC. Moving on: Factors associated with caregivers' bereavement adjustment using a random population-based face-to-face survey. Palliat Med. 2018 Jan;32(1):257–67.

3. Chen C, Tang S. Profiles of grief, post-traumatic stress, and post-traumatic growth among people bereaved due to COVID-19. Eur J Psychotraumatology. 2021 Jan;12(1):1947563.

4. Eisma MC, Rosner R, Comtesse H. ICD-11 Prolonged Grief Disorder Criteria: Turning Challenges Into Opportunities With Multiverse Analyses. Front Psychiatry. 2020 Aug 7;11:752.

5. Harrison O, Windmann S, Rosner R, Steil R. Inclusion of the other in the self as a potential risk factor for prolonged grief disorder: A comparison of patients with matched bereaved healthy controls. Clin Psychol Psychother. 2022 May;29(3):1101–12.

6. Harrison O, Wiedenmann C, Rosner R, Steil R. Mental Imagery in Patients with Prolonged Grief Disorder: a Comparison with Matched Bereaved Healthy Controls. Psychiatr Q. 2021 Dec;92(4):1361–79.

7. Karatzias T, Knefel M, Maercker A, Cloitre M, Reed G, Bryant RA, Ben-Ezra M, Kazlauskas E, Jowett S, Shevlin M, Hyland P. The Network Structure of ICD-11 Disorders Specifically Associated with Stress: Adjustment Disorder, Prolonged Grief Disorder, Posttraumatic Stress Disorder, and Complex Posttraumatic Stress Disorder. Psychopathology. 2022;55(3–4):226–34.

8. Kokou-Kpolou CK, Lenferink LIM, Brunnet AE, Park S, Megalakaki O, Boelen P, Cénat JM. The ICD-11 and DSM-5-TR prolonged grief criteria: Validation of the Traumatic Grief Inventory-Self Report Plus using exploratory factor analysis and item response theory. Clin Psychol Psychother. 2022 Nov;29(6):1950–62.

9. Lavenda O, Mahat-Shamir M, Lorenz L, Hamama-Raz Y, Greenblatt-Kimron L, Pitcho-Prelorentzos S, Ring L, Bar-Shua E, Ben-Ezra M. Revalidation of Adjustment Disorder–New Module-4 screening of adjustment disorder in a non-clinical sample: Psychometric reevaluation and correlates with other *ICD-11* mental disorders. PsyCh J. 2019 Sep;8(3):378–85.

10. O'Connor M, Vang ML, Shevlin M, Elklit A, Komischke-Konnerup KB, Lundorff M, Bryant R. Development and validation of the Aarhus PGD scale for operationalizing ICD-11 and DSM-5-TR Prolonged Grief Disorder. J Affect Disord. 2023 Dec;342:201–9.

11. Prigerson HG, Maciejewski PK. Rebuilding Consensus on Valid Criteria for Disordered Grief. JAMA Psychiatry. 2017 May 1;74(5):435.

12. Shear* MK, Mauro C, Wang Y, Skritskaya N, Reynolds C, SImon N, Zisook S, Lebowitz B, Michael F. ACNP 53rd Annual Meeting: Poster Session II. In: Neuropsychopharmacology [Internet]. Phoenix, Arizona; 2014 [cited 2024 May 19]. p. S291–472. Available from: https://www.nature.com/articles/npp2014281

13. Smith KV, Wild J, Ehlers A. Psychometric Characteristics of the Oxford Grief Memory Characteristics Scale and Its Relationship With Symptoms of ICD-11 and DSM-5-TR Prolonged Grief Disorder. Front Psychiatry. 2022 Mar 16;13:814171.

14. Song C, Fu Z, Li W, Yu Y, Ma J, Li X, Wang J. Social support and prolonged grief disorder symptoms in parents who lost their only child from urban and rural China. Psychol Trauma Theory Res Pract Policy. 2023 Feb;15(2):304–12.

15. Song H, Fang F, Larsson H, Pedersen NL, Magnusson PK, Almqvist C, Valdimarsdóttir UA. Loss of a co-twin at birth and subsequent risk of psychiatric disorders. eLife. 2021 Jan 28;10:e63514.

16. Stelzer EM, Höltge J, Zhou N, Maercker A, Killikelly C. Cross-cultural generalizability of the ICD-11 PGD symptom network: Identification of central symptoms and culturally specific items across German-speaking and Chinese bereaved. Compr Psychiatry. 2020 Nov;103:152211.

17. Tay AK, Rees S, Chen J, Kareth M, Silove D. Factorial structure of complicated grief: associations with loss-related traumatic events and psychosocial impacts of mass conflict amongst West Papuan refugees. Soc Psychiatry Psychiatr Epidemiol. 2016 Mar;51(3):395–406.

18. Xu X, Xie T, Zhou N, Shi G, Wen J, Wang J, Li X, Poppen PJ. Network analysis of PGD, PTSD and insomnia symptoms in Chinese shidu parents with PGD. Eur J Psychotraumatology. 2022 Jul 29;13(1):2057674.

19. Comtesse H, Vogel A, Kersting A, Rief W, Steil R, Rosner R. When does grief become pathological? Evaluation of the ICD-11 diagnostic proposal for prolonged grief in a treatment-seeking sample. Eur J Psychotraumatology. 2020 Dec 31;11(1):1694348.

20. Hennemann S, Killikelly C, Hyland P, Maercker A, Witthöft M. Somatic symptom distress and ICD-11 prolonged grief in a large intercultural sample. Eur J Psychotraumatology. 2023 Nov 23;14(2):2254584.

21. Lundorff M, Bonanno GA, Johannsen M, O'Connor M. Are there gender differences in prolonged grief trajectories? A registry-sampled cohort study. J Psychiatr Res. 2020 Oct;129:168–75.

22. Shevlin M, Redican E, Hyland P, Murphy J, Karatzias T, McBride O, Bennett K, Butter S, Hartman TK, Vallières F, Bentall RP. Symptoms and levels of ICD-11 Prolonged Grief Disorder in a representative community sample of UK adults. Soc Psychiatry Psychiatr Epidemiol. 2023 Oct;58(10):1535–47.

23. Vang ML, Prigerson HG, Elklit A, Komischke-Konnerup KB, O'Connor M. Do we all grieve the same? A multigroup test of the dimensional structure of prolonged grief disorder among Danish bereaved partners and children. Psychiatry Res. 2022 Dec;318:114937.

24. Lenferink LIM, Eisma MC, Smid GE, De Keijser J, Boelen PA. Valid measurement of DSM-5 persistent complex bereavement disorder and DSM-5-TR and ICD-11 prolonged grief disorder: The Traumatic Grief Inventory-Self Report Plus (TGI-SR+). Compr Psychiatry. 2022 Jan;112:152281.

Supplement F

Table 1: Items 1-6a of "Table 3: Elements of the utilized data extraction tool for every included study"

Bibliographic information		Inclusion/ Exclusion	Reason for exclusion	Year of assessment (beginning)	Year of assessment (end)	Location of data collection	Location of affiliated institution	Study type
First author, publication year	Title							
Aoun, 2020 [1]	Grief, depression, and anxiety in bereaved caregivers of people with motor neurone disease: a population-based national study	Inclusion	N/A	2019	2019	Australia	Australia	Cross-sectional
Ben-Ezra, 2020 [2]	A cross-country psychiatric screening of ICD-11 disorders specifically associated with stress in Kenya, Nigeria and Ghana	Inclusion	N/A	No information	No information	African Cross-national	Israel, Ireland, Scotland, Switzerland and Northern Ireland	Cross-sectional
						Nigeria		
						Kenya		
						Ghana		
Boelen, 2020 [3]	Comparison of six proposed diagnostic criteria sets for disturbed grief	Inclusion	N/A	No information	No information	Netherlands	Netherlands	Cross-sectional
Boelen, 2018 [4]	Evaluation of the factor structure, prevalence, and validity of disturbed grief in DSM-5 and ICD-11	Inclusion	N/A	No information	No information	Netherlands	Netherlands and Australia	Longitudinal
Boelen, 2019 [5]	Further evaluation of the factor structure, prevalence, and concurrent validity of DSM-5 criteria for Persistent Complex Bereavement Disorder and ICD-11 criteria for Prolonged Grief Disorder	Inclusion	N/A	No information	No information	No information	Netherlands	Cross-sectional
Boelen, Spuji, 2019 [6]	Comparison of DSM-5 criteria for persistent complex bereavement disorder and ICD-11 criteria for prolonged grief disorder in help-seeking bereaved children	Inclusion	N/A	No information	No information	No information	Netherlands	Cross-sectional
Bonanno, 2020 [7]	Trajectories of grief: Comparing symptoms from the DSM-5 and ICD-11 diagnoses	Inclusion	N/A	No information	No information	No information	USA	Cross-sectional
Eberle, 2023 [8]	Stress-associated symptoms and disorders: A transdiagnostic comparison	Inclusion	N/A	2021	2022	Switzerland	Switzerland	Cross-sectional
Eisma, 2023 [9]	Co-occurrence of approach and avoidance in prolonged grief: a latent class analysis	Inclusion	N/A	2019	2020	Netherlands	Netherlands	Cross-sectional

Haneveld, 2022 [10]	Same name, same content? Evaluation of DSM-5-TR and ICD-11 prolonged grief criteria	Inclusion	N/A	2018	2019	Germany	Germany	Cross-sectional
Hyland, 2024 [11]	The International Grief Questionnaire (IGQ): A new measure of ICD-11 prolonged grief disorder	Inclusion	N/A	2022	2022	Republic of Ireland<br>United Kingdom	Ireland and United Kingdom	Cross-sectional
Killikelly, 2023 [12]	Measurement and assessment of grief in a large international sample	Inclusion	N/A	2019	2020	Cross-national<br>Greece<br>Cyprus<br>Turkey<br>Iran<br>USA	Switzerland, Canada, Greece, Germany, USA, Iran and Turkey	Cross-sectional
Killikelly, 2019 [13]	Prolonged grief disorder: Its co-occurrence with adjustment disorder and post-traumatic stress disorder in a bereaved Israeli general-population sample	Inclusion	N/A	No information	No information	Israel	Switzerland and Israel	Cross-sectional
Kokou-Kpolou, 2020 [14]	A comparison of prevalence and risk factor profiles of prolonged grief disorder among French and Togolese bereaved adults	Inclusion	N/A	2015	2017	France<br>Togo	France, Canada and USA	Cross-sectional
Lenferink, 2022 [15]	Valid measurement of DSM-5 persistent complex bereavement disorder and DSM-5-TR and ICD-11 prolonged grief disorder: The Traumatic Grief Inventory-Self Report Plus (TGI-SR+)	Inclusion	N/A	2018	2020	Netherlands	Netherlands	Cross-sectional
Lenferink, 2023 [16]	The Traumatic Grief Inventory-Clinician Administered: A psychometric evaluation of a new interview for ICD-11 and DSM-5-TR prolonged grief disorder severity and probable caseness	Inclusion	N/A	2019	2020	Germany<br>Netherlands	Germany and Netherlands	Cross-sectional
Lenferink, van Dijk, 2024 [17]	Psychometric evaluation of the Swedish Traumatic Grief Inventory Self-Report Plus (TGI-SR+) in bereaved parents	Inclusion	N/A	No information	No information	Sweden	Netherlands, Sweden and Norway	Cross-sectional
Lundorff, 2021[18]	Time elapsed since loss or grief persistency? Prevalence and predictors of ICD-11 prolonged grief disorder using different applications of the duration criterion	Inclusion	N/A	No information	No information	Denmark	Denmark	Longitudinal
Redican, 2023 [19]	Operationalization, implications and correlates of the cultural deviance criterion for ICD-11 and DSM-5 prolonged grief disorder	Inclusion	N/A	No information	No information	Denmark	United Kingdom and Denmark	

Maciejewski, 2016 [20]	"Prolonged grief disorder" and "persistent complex bereavement disorder", but not "complicated grief", are one and the same diagnostic entity: an analysis of data from the Yale Bereavement Study	Inclusion	N/A	No information	No information	USA	USA, Switzerland and Netherlands	Cross-sectional
Matthews, 2019 [21]	Posttraumatic Stress Disorder, Depression, and Prolonged Grief Disorder in Families Bereaved by a Traumatic Workplace Death: The Need for Satisfactory Information and Support	Inclusion	N/A	2013	2015	Australia<br>Canada<br>USA<br>United Kingdom<br>Total Cohort	Australia	Cross-sectional
Mauro, 2019 [22]	Prolonged grief disorder: clinical utility of ICD-11 diagnostic guidelines	Inclusion	N/A	2010	2014	USA	USA	Cross-sectional
Nielsen, 2020 [23]	Exploring Functional Impairment in Light of Prolonged Grief Disorder: A Prospective, Population-Based Cohort Study	Inclusion	N/A	2012	2012	Denmark	Denmark	Longitudinal
O'Connor, 2019 [24]	Comparison of proposed diagnostic criteria for pathological grief using a sample of elderly bereaved spouses in Denmark: Perspectives on future bereavement research	Inclusion	N/A	2006	2007	Denmark	Denmark and Netherlands	Cross-sectional
Peinado, 2024 [25]	ICD-11 prolonged grief disorder: Prevalence, predictors, and co-occurrence in a large representative sample	Inclusion	N/A	2021	2021	Spain	Spain and United Kingdom	Cross-sectional
Redican, 2024 [26]	Prevalence and correlates of ICD-11 prolonged grief disorder among adults living in Ukraine during the war with Russia	Inclusion	N/A	2023	2023	Ukraine	United Kingdom, Ireland, Ukraine and Israel	Cross-sectional
Rosner, 2021 [27]	Prevalence of prolonged grief disorder	Inclusion	N/A	2019	2019	Germany	Germany	Cross-sectional
Shevlin, 2023 [28]	Symptoms and levels of ICD-11 Prolonged Grief Disorder in a representative community sample of UK adults	Inclusion	N/A	No information	No information	United Kingdom	United Kingdom and Ireland	Cross-sectional
Siddiqua, 2024 [29]	The smoldering pandemic: Prevalence of prolonged grief disorder. A cross-sectional study of bereaving adults during the COVID pandemic in Pakistan	Inclusion	N/A	2021	2021	Pakistan	Pakistan and USA	Cross-sectional
Singer, 2022 [30]	An evaluation of common risk factors for prolonged grief disorder using the international classification of diseases-11 criteria	Inclusion	N/A	No information	No information	USA	USA	Cross-sectional

Specht, 2022 [31]	Prolonged grief disorder in Arabic-speaking treatment-seeking populations: Relationship with socio-demographic aspects, loss- and trauma-related characteristics, and mental health support	Inclusion	N/A	2021	2022	Germany	Germany	Cross-sectional
Tay, 2019 [32]	The structure and psychosocial correlates of complicated bereavement amongst refugees from West Papua	Inclusion	N/A	2016	2016	Papua New Guinea	Australia	Cross-sectional
Treml, 2024 [33]	Prolonged grief disorder in ICD-11 and DSM-5-TR: differences in prevalence and diagnostic criteria	Inclusion	N/A	2021	2021	Germany	Germany	Cross-sectional
Zhang, 2020 [34]	Prolonged grief disorder in Chinese Shidu parents who have lost their only child	Inclusion	N/A	2015	2017	China	China	Cross-sectional
Zhou, 2020 [35]	Prevalence and associated factors of prolonged grief disorder in Chinese parents bereaved by losing their only child	Inclusion	N/A	2017	2018	China	China, Switzerland and USA	Cross-sectional

Table 2: Items 1a-b, 5, 6b-c, and 7a of "Table 3: Elements of the utilized data extraction tool for every included study"

**First author, publication year**	**Sample characteristics**					
	**Setting**	**Inclusion/Exclusion criteria**	**Location of Data Collection (Nation)**	**Location of affiliated institution (Nation)**	**Potential added specification**	**Sample size**

Aoun, 2020 [1]	Target population:<br>• Family caregivers bereaved through MND between 2016 and 2018<br><br>Was the study population described as representative?<br>• No<br><br>Recruitment process:<br>• Survey conducted across Australia<br>• Participants included clients from the five state Motor Neurone Disease (MND) Associations<br>• Study packets, containing an invitation letter from the respective MND association, an information sheet, the questionnaire, a list of support services for families, and a pre-paid return envelope, were sent to these five MND Associations<br>• The front of the questionnaire provided the online survey link<br><br>Survey type:<br>• Postal or online survey	Bereavement or specification of type of bereavement:<br>• Loss of a relative/friend between 2016 and 2018<br><br>Time since bereavement:<br>• Not applicable<br><br>Elevated grief symptoms:<br>• Not applicable<br><br>Language or writing reading level/proficiency:<br>• Ability to read and write in English<br><br>Age criterion:<br>• > 18 years of age<br><br>Citizenship:<br>• Not applicable<br><br>Residence:<br>• Not applicable<br><br>Nature of death:<br>• Due to MND<br><br>Health status:<br>• Not applicable<br><br>Other:<br>• Not applicable	Australia	Australia	None	381
Ben-Ezra, 2020 [2]	Target population:<br>• Representative national samples of Nigeria, Kenya and Ghana<br><br>Was the study population described as representative?<br>• Yes, representativeness of study population based on Age and Sex in the African countries' census	Bereavement or specification of type of bereavement:<br>• Not applicable<br><br>Time since bereavement:<br>• Not applicable<br><br>Elevated grief symptoms:<br>• Not applicable	African Cross-national	Israel, Ireland, Scotland, Switzerland and Northern Ireland	None	2524
			Nigeria			1006

	Recruitment process:<br>• Internet panels were used to obtain the study samples<br><br>Survey type:<br>• Online survey	Language or writing reading level/proficiency:<br>• Sufficient English proficiency to complete the survey<br><br>Age criterion:<br>• > 18 years of age<br><br>Citizenship:<br>• Citizenship of Nigeria, Kenya or Ghana<br><br>Residence:<br>• Not applicable<br><br>Nature of death:<br>• Not applicable<br><br>Health status:<br>• Not applicable<br><br>Other:<br>• Not applicable	Kenya			1018
			Ghana			500
Boelen, 2020 [3]	Target population:<br>• Dutch bereaved individuals<br><br>Was the study population described as representative?<br>• No<br><br>Recruitment process:<br>• Data gathered from previous studies<br>• This study utilized data from 855 Dutch individuals who had experienced bereavement, collected as part of a research program on cognitive-behavioral factors in grief<br>• Data were obtained from various sources<br>• The initial group was recruited through professional and non-professional bereavement caregivers who distributed the questionnaires<br>• Additional participants were recruited through online announcements<br><br>Survey type:<br>• Online or postal survey	Bereavement or specification of type of bereavement:<br>• Bereavement<br><br>Time since bereavement:<br>• ≥ 12 months earlier<br><br>Elevated grief symptoms:<br>• Not applicable<br><br>Language or writing reading level/proficiency:<br>• Not applicable<br><br>Age criterion:<br>• ≥ 18 years<br><br>Citizenship:<br>• Not applicable<br><br>Residence:<br>• Not applicable<br><br>Nature of death:<br>• Not applicable<br><br>Health status:<br>• Not applicable<br><br>Other:<br>• Not applicable	Netherlands	Netherlands	None	855

Boelen, 2018 [4]	Target population:<br>• Dutch bereaved community<br><br>Was the study population described as representative?<br>• No<br><br>Recruitment process:<br>• Initially gathered as part of an IRB-approved research project conducted in the Netherlands<br>• Professional and lay mental health care providers recruited participants (e.g., grief counselors, therapists) and distributed questionnaires to grieving individuals<br><br>Survey type:<br>• Written questionnaire	Bereavement or specification of type of bereavement:<br>• Bereavement<br><br>Time since bereavement:<br>• > 5 months < 10 years ago<br><br>Elevated grief symptoms:<br>• Not applicable<br><br>Language or writing reading level/proficiency:<br>• Not applicable<br><br>Age criterion:<br>• ≥ 18 years<br><br>Citizenship:<br>• Not applicable<br><br>Residence:<br>• Not applicable<br><br>Nature of death:<br>• Not applicable<br><br>Health status:<br>• Not applicable<br><br>Other:<br>• Not applicable	Netherlands	Netherlands and Australia	Study population at time point 1<br><br>Study population at time point 2	512<br><br>280
Boelen, 2019 [5]	Target population:<br>• Bereaved individuals<br><br>Was the study population described as representative?<br>• No<br><br>Recruitment process:<br>• Data were sourced from 551 bereaved individuals involved in two consecutive studies focusing on the characteristics and correlates of disturbed grief<br>• Participants for both studies were recruited through online advertisements<br>• Upon completing an application form, participants were either sent paper questionnaires or provided access to a secure website to complete the questionnaires online	Bereavement or specification of type of bereavement:<br>• Bereavement<br><br>Time since bereavement:<br>• ≥ 6 months and ≤ 20 years ago<br><br>Elevated grief symptoms:<br>• Not applicable<br><br>Language or writing reading level/proficiency:<br>• Not applicable<br><br>Age criterion:<br>• > 17 years<br><br>Citizenship:<br>• Not applicable<br><br>Residence:<br>• Not applicable	No information	Netherlands	None	551

	Survey type:<br>• Online or postal survey	Nature of death:<br>• Not applicable<br><br>Health status:<br>• Not applicable<br><br>Other:<br>• Not applicable				
Boelen, Spuji, 2019 [6]	Target population:<br>• Children seeking psychological help following their bereavement<br><br>Was the study population described as representative?<br>• No<br><br>Recruitment process:<br>• Data were obtained from 332 children and adolescents, aged 8–18 years, who participated in a research program exploring the nature and correlates of children's grief<br>• Roughly a third of the children were recruited from bereavement support organizations and completed questionnaires with a research assistant during a home visit, while the other two thirds were recruited through various outpatient clinics and completed questionnaires with their therapists present<br><br>Survey type:<br>• Written questionnaires in the presence of their therapists	Bereavement or specification of type of bereavement:<br>• Bereavement<br><br>Time since bereavement:<br>• > 6 months<br><br>Elevated grief symptoms:<br>• Not applicable<br><br>Language or writing reading level/proficiency:<br>• Not applicable<br><br>Age criterion:<br>• Not applicable<br><br>Citizenship:<br>• Not applicable<br><br>Residence:<br>• Not applicable<br><br>Nature of death:<br>• Not applicable<br><br>Health status:<br>• Not applicable<br><br>Other:<br>• Not applicable	No information	Netherlands	None	291
Bonanno, 2020 [7]	Target population:<br>• Bereaved individuals<br><br>Was the study population described as representative?<br>• No	Bereavement or specification of type of bereavement:<br>• Loss of a spouse<br><br>Time since bereavement:<br>• Not applicable	No information	USA	Study population at time point 1	282

	Recruitment process:<br>• Based on public death listings, obituaries, support group referrals invitation letters were sent and flyers, internet, and newspaper advertised the study<br>• Bereavement status was confirmed using death certificates<br><br>Survey type:<br>• Structured clinical assessment	Elevated grief symptoms:<br>• Not applicable<br><br>Language or writing reading level/proficiency:<br>• Not applicable<br><br>Age criterion:<br>• Not applicable<br><br>Citizenship:<br>• Not applicable<br><br>Residence:<br>• Not applicable<br><br>Nature of death:<br>• Not applicable<br><br>Health status:<br>• Not applicable<br><br>Other:<br>• Individuals for whom data were available for at least two time points			Study population at time point 2	
					Study population at time point 3	206

Eberle, 2023 [8]	Target population:<br>• Not described<br><br>Was the study population described as representative?<br>• No<br><br>Recruitment process:<br>• Public advertisements, personal contacts, mailing lists, and professional networks were used for recruitment<br><br>Survey type:<br>• Online survey	Bereavement or specification of type of bereavement:<br>• Not applicable<br><br>Time since bereavement:<br>• Not applicable<br><br>Elevated grief symptoms:<br>• Not applicable<br><br>Language or writing reading level/proficiency:<br>• Not applicable<br><br>Age criterion:<br>• ≥ 18 years<br><br>Citizenship:<br>• Not applicable<br><br>Residence:<br>• Not applicable<br><br>Nature of death:<br>• Not applicable<br><br>Health status:<br>• Not applicable<br><br>Other:<br>• Not applicable	Switzerland	Switzerland	None	447

Eisma, 2023 [9]	Target population:<br>• Dutch adults who experienced the death of a loved one<br><br>Was the study population described as representative?<br>• No<br><br>Recruitment process:<br>• Recruitment through Google Ads<br><br>Survey type:<br>• Online survey	Bereavement or specification of type of bereavement:<br>• Bereavement<br><br>Time since bereavement:<br>• > 12 months<br><br>Elevated grief symptoms:<br>• Not applicable<br><br>Language or writing reading level/proficiency:<br>• Not applicable<br><br>Age criterion:<br>• Not applicable<br><br>Citizenship:<br>• Not applicable<br><br>Residence:<br>• Not applicable<br><br>Nature of death:<br>• Not applicable<br><br>Health status:<br>• Not applicable<br><br>Other:<br>• Not applicable	Netherlands	Netherlands	None	288

Haneveld, 2022 [10]	Target population:<br>• Treatment-seeking bereaved sample<br><br>Was the study population described as representative?<br>• No<br><br>Recruitment process:<br>• Participants were recruited for an ongoing multicenter randomized controlled trial (RCT) to compare a grief-focused integrative cognitive-behavioral therapy with an active control condition, present-centered therapy<br>• The study was conducted at four university outpatient clinics in Germany<br>• The study utilized data from participants included as pilot cases (each therapist treated a supervised pilot case before the main trial), from main trial participants, and from patients excluded from the pilot or main trial<br><br>Survey type:<br>• Structured clinical assessment	Bereavement or specification of type of bereavement:<br>• Bereavement<br><br>Time since bereavement:<br>• ≥ 6 months<br><br>Elevated grief symptoms:<br>• Exceed the cut-off of 20 in the German version of the Inventory of Complicated Grief<br><br>Language or writing reading level/proficiency:<br>• Not applicable<br><br>Age criterion:<br>• 18–75 years old<br><br>Citizenship:<br>• Not applicable<br><br>Residence:<br>• Not applicable<br><br>Nature of death:<br>• Not applicable<br><br>Health status:<br>• Not applicable<br><br>Other:<br>• Not applicable	Germany	Germany	None	193

Hyland, 2024 [11]	Target population:<br>• Bereaved adults in the United Kingdom and Ireland<br><br>Was the study population described as representative?<br>• No<br><br>Recruitment process:<br>• Data were gathered from two bereaved adult samples from the United Kingdom and the Republic of Ireland<br>• The survey company Qualtrics collected these data, recruiting participants in each country from existing, actively managed, double opt-in research panels via email, text message, or in-app notifications<br><br>Survey type:<br>• No information	Bereavement or specification of type of bereavement:<br>• Lifetime bereavement<br><br>Time since bereavement:<br>• Not applicable<br><br>Elevated grief symptoms:<br>• Not applicable<br><br>Language or writing reading level/proficiency:<br>• Not applicable<br><br>Age criterion:<br>• ≥ 18 years<br><br>Citizenship:<br>• Not applicable<br><br>Residence:<br>• Not applicable<br><br>Nature of death:<br>• Not applicable<br><br>Health status:<br>• Not applicable<br><br>Other:<br>• Not applicable	Republic of Ireland	Ireland and United Kingdom	Irish study population	1011
			United Kingdom		UK study population	1012
Killikelly, 2023 [12]	Target population:<br>• None described<br><br>Was the study population described as representative?<br>• No<br><br>Recruitment process:<br>• The international sample was obtained from various countries through the Psi Chi Network for International Collaborative Exchange (NICE: CROWD) project<br>• The research sample was collected in five countries utilizing snowball sampling via social media platforms and institutional email lists in each respective	Bereavement or specification of type of bereavement:<br>• Bereavement<br><br>Time since bereavement:<br>• ≥ 6 months ≤10 years<br><br>Elevated grief symptoms:<br>• Not applicable<br><br>Language or writing reading level/proficiency:<br>• Not applicable<br><br>Age criterion:<br>• ≥ 18 years<br><br>Citizenship:	Cross-national	Switzerland, Canada, Greece, Germany, USA, Iran and Turkey	Cross-national sample	1393
			Greece		Greek sample	202
			Cyprus		Cypriot sample	

	country<br><br>Survey type:<br>• Online survey	• Not applicable<br><br>Residence:<br>• Not applicable<br><br>Nature of death:<br>• Not applicable<br><br>Health status:<br>• Not applicable<br><br>Other:<br>• Not applicable	Turkey		Turkish sample	343
			Iran		Iranian sample	
			USA		American sample	848
Killikelly, 2019 [13]	Target population:<br>• Bereaved Israeli population sample<br><br>Was the study population described as representative?<br>• Yes, close approximation of a general population sample<br><br>Recruitment process:<br>• The sample for this study was sourced from a national panel of 200,000 Israeli citizens<br>• Stratified and random sampling methods were employed to closely approximate a representative general population sample<br>• Out of all participants only those fulfilling the inclusion criteria constituted the sample for the present analysis<br>• Participants were recruited via online platforms<br><br>Survey type:<br>• No information	Bereavement or specification of type of bereavement:<br>• Bereavement<br><br>Time since bereavement:<br>• "recent years"<br><br>Elevated grief symptoms:<br>• Not applicable<br><br>Language or writing reading level/proficiency:<br>• Fluent in Hebrew<br><br>Age criterion:<br>• > 18 years<br><br>Citizenship:<br>• Not applicable<br><br>Residence:<br>• Not applicable<br><br>Nature of death:<br>• Not applicable<br><br>Health status:<br>• Not applicable<br><br>Other:<br>• Not applicable	Israel	Switzerland and Israel	None	544

Kokou-Kpolou, 2020 [14]	Target population:<br>• French bereaved adults<br><br>Was the study population described as representative?<br>• No<br><br>Recruitment process:<br>• French participants were recruited from online support groups and bereavement support groups in Catholic parish churches located in Amiens, a city in northern France<br>• For the church group, potential participants who had lost their spouse were identified using a death registry from 2014 to 2017<br>• These individuals were sent a postal package containing printed questionnaires<br><br>Survey type:<br>• Postal questionnaires	Bereavement or specification of type of bereavement:<br>• Loss of a partner<br><br>Time since bereavement:<br>• ≥ 6 months<br><br>Elevated grief symptoms:<br>• Not applicable<br><br>Language or writing reading level/proficiency:<br>• Not applicable<br><br>Age criterion:<br>• Not applicable<br><br>Citizenship:<br>• Not applicable<br><br>Residence:<br>• Not applicable<br><br>Nature of death:<br>• Not applicable<br><br>Health status:<br>• Not applicable<br><br>Other:<br>• Not applicable	France	France, Canada and USA	French population	73
	Target population:<br>• Togolese bereaved adults<br><br>Was the study population described as representative?<br>• No<br><br>Recruitment process:<br>• The survey was administered in three urban areas and seven rural locations<br>• Participants were enlisted through mutual-help groups for widowed individuals<br>• Survey supervisors distributed packages of printed questionnaires to participants during their monthly meetings<br><br>Survey type:<br>• Printed questionnaires	Bereavement or specification of type of bereavement:<br>• Loss of a partner<br><br>Time since bereavement:<br>• ≥ 6 months<br><br>Elevated grief symptoms:<br>• Not applicable<br><br>Language or writing reading level/proficiency:<br>• Sufficient education level to read and write<br><br>Age criterion:<br>• Not applicable<br><br>Citizenship:<br>• Not applicable<br><br>Residence:<br>• Not applicable	Togo		Togolese population	162

		Nature of death:<br>• Not applicable<br><br>Health status:<br>• Not applicable<br><br>Other:<br>• Not applicable				
Lenferink, 2022 [15]	Target population:<br>• Dutch adults who lost loved ones in a traffic accident<br><br>Was the study population described as representative?<br>• No<br><br>Recruitment process:<br>• Data were gathered as part of the ongoing "TrafVic project," focused on examining the consequences and care following the death of a loved one in a traffic accident<br>• The majority of participants were recruited through Victim Support, a Dutch organization that offers practical, judicial, and emotional support to trauma victims; additional sources included social media (e.g., Facebook) and referrals from family members or friends<br>• The remaining participants were recruited through various other channels<br><br>Survey type:<br>• Online survey	Bereavement or specification of type of bereavement:<br>• Loss of spouse, family member or friend<br><br>Time since bereavement:<br>• ≥ 12 months<br><br>Elevated grief symptoms:<br>• Not applicable<br><br>Language or writing reading level/proficiency:<br>• Not applicable<br><br>Age criterion:<br>• "Adults"<br><br>Citizenship:<br>• Not applicable<br><br>Residence:<br>• Not applicable<br><br>Nature of death:<br>• Not applicable<br><br>Health status:<br>• Not applicable<br><br>Other:<br>• Not applicable	Netherlands	Netherlands	None	280

Lenferink, 2023 [16]	Target population:<br>• Dutch and German bereaved adults<br><br>Was the study population described as representative?<br>• No<br><br>Recruitment process:<br>• Several recruitment methods were utilized, such as social media advertisements, convenience sampling (researchers contacting their personal networks), and snowball sampling (participants referring others to the study)<br>• First-year psychology students were given the opportunity to earn course credits by participating<br><br>Survey type:<br>• Telephone interview	Bereavement or specification of type of bereavement:<br>• Death of spouse, family member, or friend<br><br>Time since bereavement:<br>• ≥ 6 moths<br><br>Elevated grief symptoms:<br>• Not applicable<br><br>Language or writing reading level/proficiency:<br>• Not applicable<br><br>Age criterion:<br>• "Adults"<br><br>Citizenship:<br>• Not applicable<br><br>Residence:<br>• Not applicable<br><br>Nature of death:<br>• Not applicable<br><br>Health status:<br>• Excluded when they were suicidal and/or had been diagnosed with a psychotic disorder (assessed with single items)<br><br>Other:<br>• Not applicable	Germany	Germany and Netherlands	German study population	222
			Netherlands		Dutch study population	211

Lenferink, van Dijk, 2024 [17]	Target population:<br>• Bereaved parents living in Sweden<br><br>Was the study population described as representative?<br>• No<br><br>Recruitment process:<br>• Participants in this study are individuals involved in a randomized controlled trial assessing the effectiveness of a self-help app, My Grief, designed to assist bereaved parents in the grieving process<br>• Potential participants were identified by locating children diagnosed with a malignancy who died between 2010 and 2020 through the Swedish Childhood Cancer registry and the Cause of Death Registry, and subsequently identifying their parents via the Swedish Population Register at the Swedish Tax Agency<br>• Invitation letters were sent to potential participants by post between September and November 2021<br>• Additional participants were recruited through social media and newsletter advertisements from two non-profit organizations for bereaved parents in Sweden<br><br>Survey type:<br>• Online questionnaire	Bereavement or specification of type of bereavement:<br>• Loss of a child<br><br>Time since bereavement:<br>• Between 1 and 10 years ago<br><br>Elevated grief symptoms:<br>• Elevated symptom levels of PG (Prolonged Grief Disorder-13, cut-off >16)<br><br>Language or writing reading level/proficiency:<br>• Ability to understand and respond in Swedish<br><br>Age criterion:<br>• Not applicable<br><br>Citizenship:<br>• Not applicable<br><br>Residence:<br>• Not applicable<br><br>Nature of death:<br>• Not applicable<br><br>Health status:<br>• Exclusion criteria were self-reported ongoing suicidal thoughts or psychosis, assessed with single items in the screening questionnaire<br><br>Other:<br>• Access to a smartphone	Sweden	Netherlands, Sweden and Norway	None	248

Lundorff, 2021 [18]	Target population:<br>• Register-sampled cohort of bereaved spouses<br><br>Was the study population described as representative?<br>• No<br><br>Recruitment process:<br>• This study utilizes data from The Aarhus Bereavement Study (TABstudy), a multi-wave cohort study examining factors related to normal and pathological bereavement adjustment<br>• The study sample was identified using data from the Danish Civil Registration System (DCRS)<br>• Eligible participants were initially sent a condolence letter, followed by recruitment through telephone interviews<br><br>Survey type:<br>• Postal survey	Bereavement or specification of type of bereavement:<br>• Loss of a spouse from January 2017 to March 2018<br><br>Time since bereavement:<br>• Not applicable<br><br>Elevated grief symptoms:<br>• Not applicable<br><br>Language or writing reading level/proficiency:<br>• Not applicable<br><br>Age criterion:<br>• ≥ 25 years<br><br>Citizenship:<br>• Not applicable<br><br>Residence:<br>• Metropolitan area of Aarhus<br><br>Nature of death:<br>• Not applicable<br><br>Health status:<br>• Not applicable<br><br>Other:<br>• Not applicable	Denmark	Denmark	Study population at time point 1	847
					Study population at time point 2	777
					Study population at time point 3	753

Redican, 2023 [19]	Target population:<br>• Danish bereaved spouses<br><br>Was the study population described as representative?<br>• No<br><br>Recruitment process:<br>• The data for this study were obtained from The Aarhus Bereavement Study (TAB Study), an ongoing longitudinal cohort study exploring grief trajectories in adults who have lost a spouse or parent<br>• Participants were identified using the Danish Civil Registration System<br><br>Survey type:<br>• No information	Bereavement or specification of type of bereavement:<br>• Loss of a spouse<br><br>Time since bereavement:<br>• Not applicable<br><br>Elevated grief symptoms:<br>• Not applicable<br><br>Language or writing reading level/proficiency:<br>• Not applicable<br><br>Age criterion:<br>• ≥ 18 years<br><br>Citizenship:<br>• Not applicable<br><br>Residence:<br>• Metropolitan area of Aarhus<br><br>Nature of death:<br>• Not applicable<br><br>Health status:<br>• Not applicable<br><br>Other:<br>• Not applicable	Denmark	United Kingdom and Denmark	Study population at time point 4	584

Maciejewski, 2016 [20]	Target population:<br>• Community-dwelling bereaved individuals<br><br>Was the study population described as representative?<br>• No<br><br>Recruitment process:<br>• Not described<br><br>Survey type:<br>• Interview	Bereavement or specification of type of bereavement:<br>• Bereavement<br><br>Time since bereavement:<br>• Not described<br><br>Elevated grief symptoms:<br>• Not described<br><br>Language or writing reading level/proficiency:<br>• Not described<br><br>Age criterion:<br>• Not described<br><br>Citizenship:<br>• Not described<br><br>Residence:<br>• Not described<br><br>Nature of death:<br>• Not described<br><br>Health status:<br>• Not described<br><br>Other:<br>• Not described	USA	USA, Switzerland and Netherlands	None	268
Matthews, 2019 [21]	Target population:<br>• Family members affected by work deaths<br><br>Was the study population described as representative?<br>• No<br><br>Recruitment process:<br>• Due to the hidden nature of the population, participants were recruited through an advertisement detailing the study and providing the web address for the study's home page<br>• The advertisement was circulated through established industry and community support networks, and promoted on social media (Twitter, Facebook), trade union websites, radio interviews, industry newsletters, and newspapers	Bereavement or specification of type of bereavement:<br>• Bereavement<br><br>Time since bereavement:<br>• Not applicable<br><br>Elevated grief symptoms:<br>• Not applicable<br><br>Language or writing reading level/proficiency:<br>• Ability to read and write in English<br><br>Age criterion:<br>• > 18 years<br><br>Citizenship:<br>• Not applicable<br><br>Residence:<br>• Not applicable	Australia	Australia	Australian study population	92
			Canada		Canadian study population	25
			USA		American study population	23
			United Kingdom		UK study population	8
			Total Cohort		Entire study population	148

	• Interested family members were directed to the study's home page, where they could access an ethics-approved Participant Information Sheet with full details about the study<br><br>Survey type:<br>• Online survey	Nature of death:<br>• Sudden death of a family member through a work process-related injury<br>• Exclusion of deaths due to workplace diseases and medical conditions, such as mesothelioma, heart attack or stroke<br><br>Health status:<br>• Not applicable<br><br>Other:<br>• Not applicable				
Mauro, 2019 [22]	Target population:<br>• Bereaved participants with impairing grief<br><br>Was the study population described as representative?<br>• No<br><br>Recruitment process:<br>• Participants with persistent, impairing grief were enrolled at psychiatric research clinics affiliated with Columbia University, Massachusetts General Hospital, University of Pittsburgh Medical Center and University of California San Diego<br>• This was part of a collaborative treatment study funded by the National Institute of Mental Health (NIMH)<br><br>Survey type:<br>• Structured clinical interview	Bereavement or specification of type of bereavement:<br>• Bereavement<br><br>Time since bereavement:<br>• ≥ 6 months<br><br>Elevated grief symptoms:<br>• Positive telephone screening for impairing grief was conducted using the Brief Grief Questionnaire, with scores ⩾30 on the ICG<br><br>Language or writing reading level/proficiency:<br>• Not applicable<br><br>Age criterion:<br>• Not applicable<br><br>Citizenship:<br>• Not applicable<br><br>Residence:<br>• Not applicable<br><br>Nature of death:<br>• Not applicable<br><br>Health status:<br>• Participants were excluded if they had a current substance use disorder (within the past 6 months), a lifetime history of psychotic disorder, bipolar I disorder, cognitive impairment (Montreal Cognitive Assessment score <21 or Mini-Mental State score <24), active suicidal ideation requiring hospitalization, concurrent psychotherapy, or a pending lawsuit or disability claim related to the death<br><br>Other:<br>• Not applicable	USA	USA	Study population with impairing grief	261
	Target population:<br>• Bereaved participants without persistent impairing grief	Bereavement or specification of type of bereavement:<br>• Bereavement<br><br>Time since bereavement:			Study population without impairing grief	86

	Was the study population described as representative?<br>• No<br><br>Recruitment process:<br>• Bereaved participants who did not have persistent, impairing grief were enrolled in other ongoing research studies at the Late-Life Depression Prevention and Treatment Center at University of Pittsburgh Medical Center and the Center for Anxiety and Traumatic Stress Disorders at Massachusetts General Hospital<br><br>Survey type:<br>• No information	• Not applicable<br><br>Elevated grief symptoms:<br>• Clinical interview in which a DSM-IV mood or anxiety diagnosis was identified as the primary problem<br>• ICG score of 20 or below<br><br>Language or writing reading level/proficiency:<br>• Not applicable<br><br>Age criterion:<br>• Not applicable<br><br>Citizenship:<br>• Not applicable<br><br>Residence:<br>• Not applicable<br><br>Nature of death:<br>• Not applicable<br><br>Health status:<br>• Not applicable<br><br>Other:<br>• Not applicable				
Nielsen, 2020 [23]	Target population:<br>• Danish family caregivers<br><br>Was the study population described as representative?<br>• No<br><br>Recruitment process:<br>• Participants were identified through patients registered in the Danish National Database of Reimbursed Prescriptions due to terminal illness<br>• Each week, an information letter and questionnaire were sent to newly registered patients, requesting that their closest family caregiver complete the questionnaire<br><br>Survey type:<br>• Postal survey	Bereavement or specification of type of bereavement:<br>• Not applicable<br><br>Time since bereavement:<br>• Not applicable<br><br>Elevated grief symptoms:<br>• Not applicable<br><br>Language or writing reading level/proficiency:<br>• Not applicable<br><br>Age criterion:<br>• Not applicable<br><br>Citizenship:<br>• Not applicable<br><br>Residence:<br>• Not applicable<br><br>Nature of death:<br>• Not applicable	Denmark	Denmark	Study population at time point 1 and 2 (6 months and 36 months after bereavement)	1622

		Health status:<br>• Not applicable<br><br>Other:<br>• Completion of questionnaires at all three time points, including the functional impairment item of the PG-13 scale before bereavement (T0), and the total PG-13 at 6 months post-bereavement (T1)				
O'Connor, 2019 [24]	Target population:<br>• Elderly bereaved persons who lost a spouse<br><br>Was the study population described as representative?<br>• No<br><br>Recruitment process:<br>• Participants were identified using the Danish Civil Registration System (CPR) and were invited to participate in the study eight weeks after their loss<br><br>Survey type:<br>• Written questionnaire	Bereavement or specification of type of bereavement:<br>• Loss of spouse during 2006<br><br>Time since bereavement:<br>• Not applicable<br><br>Elevated grief symptoms:<br>• Not applicable<br><br>Language or writing reading level/proficiency:<br>• Not applicable<br><br>Age criterion:<br>• 65-80 years<br><br>Citizenship:<br>• Not applicable<br><br>Residence:<br>• County of Aarhus, Denmark<br><br>Nature of death:<br>• Not applicable<br><br>Health status:<br>• Not applicable<br><br>Other:<br>• Not applicable	Denmark	Denmark and Netherlands	Study population at time point 1 (6 months after bereavement)	206
Peinado, 2024 [25]	Target population:<br>• National sample of Spain<br><br>Was the study population described as representative?<br>• Yes<br><br>Recruitment process:<br>• Adult participants were recruited from an online research panel, utilizing quota sampling techniques to ensure the sample reflected the national population in terms of age, gender, household income, and	Bereavement or specification of type of bereavement:<br>• Not applicable<br><br>Time since bereavement:<br>• Not applicable<br><br>Elevated grief symptoms:<br>• Not applicable<br><br>Language or writing reading level/proficiency:<br>• Not applicable<br><br>Age criterion:	Spain	Spain and United Kingdom	Representative national sample	1498

	political region<br><br>Survey type:<br>• No information	• Not applicable<br><br>Citizenship:<br>• Not applicable<br><br>Residence:<br>• Not applicable<br><br>Nature of death:<br>• Not applicable<br><br>Health status:<br>• Not applicable<br><br>Other:<br>• Not applicable				
	Target population:<br>• Spanish bereaved population<br><br>Was the study population described as representative?<br>• Yes<br><br>Recruitment process:<br>• Adult participants were recruited from an online research panel, utilizing quota sampling techniques to ensure the sample reflected the national population in terms of age, gender, household income, and political region<br><br>Survey type:<br>• No information	Bereavement or specification of type of bereavement:<br>• Lifetime bereavement<br><br>Time since bereavement:<br>• > 6 months<br><br>Elevated grief symptoms:<br>• Not applicable<br><br>Language or writing reading level/proficiency:<br>• Not applicable<br><br>Age criterion:<br>• Not applicable<br><br>Citizenship:<br>• Not applicable<br><br>Residence:<br>• Not applicable<br><br>Nature of death:<br>• Not applicable<br><br>Health status:<br>• Not applicable<br><br>Other:<br>• Not applicable			Bereaved individuals from representative national sample	947

Redican, 2024 [26]	Target population:<br>• Nationwide sample of adults living in Ukraine<br><br>Was the study population described as representative?<br>• Yes<br><br>Recruitment process:<br>• Participants were drawn from an existing research panel that, before the war, was nationally representative according to the most recent Ukrainian census data<br>• To create a sample as representative as possible of the adult population of Ukraine under current circumstances, non-probability, quota sampling methods were used<br>• Quota variables included sex, age, and region of Ukraine. Table 1 shows the targeted quotas for sex, age, and region from the available census data, alongside the actual sample composition<br><br>Survey type:<br>• Online survey	Bereavement or specification of type of bereavement:<br>• Not applicable<br><br>Time since bereavement:<br>• Not applicable<br><br>Elevated grief symptoms:<br>• Not applicable<br><br>Language or writing reading level/proficiency:<br>• Capable of completing the survey in Ukrainian<br><br>Age criterion:<br>• ≥ 18 years<br><br>Citizenship:<br>• Not applicable<br><br>Residence:<br>• Ukraine<br><br>Nature of death:<br>• Not applicable<br><br>Health status:<br>• Not applicable<br><br>Other:<br>• Not applicable	Ukraine	United Kingdom, Ireland, Ukraine and Israel	None	2050
Rosner, 2021 [27]	Target population:<br>• Representative sample to the German population<br><br>Was the study population described as representative?<br>• Yes<br><br>Recruitment process:<br>• We obtained a representative sample of the German population with the help of a demographic consulting company (USUMA GmbH, Berlin, Germany)<br>• The sampling process included (a) dividing Germany into 258 sample areas representing the entire country, (b) randomly selecting households within each area using a random route procedure, and (c) randomly selecting one member from each household	Bereavement or specification of type of bereavement:<br>• Not applicable<br><br>Time since bereavement:<br>• Not applicable<br><br>Elevated grief symptoms:<br>• Not applicable<br><br>Language or writing reading level/proficiency:<br>• Sufficient German skills<br><br>Age criterion:<br>• ≥ 14 years<br><br>Citizenship:<br>• Not applicable<br><br>Residence:<br>• Ukraine	Germany	Germany	None	2498

	• The sample was representative of the German micro census concerning age, gender, and geographic region<br><br>Survey type:<br>• Interviews	Nature of death:<br>• Not applicable<br><br>Health status:<br>• Not applicable<br><br>Other:<br>• Not applicable				
Shevlin, 2023 [28]	Target population:<br>• Bereaved UK general population<br><br>Was the study population described as representative?<br>• Yes<br><br>Recruitment process:<br>• Data for the first wave of the C19PRC study were collected by the survey company Qualtrics<br>• Quota sampling methods, a non-probability-based approach where quotas of participants with specific characteristics are targeted, were employed to ensure the sample was nationally representative in terms of age, sex, and household income<br>• Data collection for the fifth wave occurred in two phases: in phase 1, adults who had participated in Wave 4 were contacted by Qualtrics via email, SMS, or in-app notifications and invited to continue their participation<br>• In phase 2 (from April 8th to April 20th, 2021), participants who had completed any earlier wave (waves one to three) were recontacted and invited to join in wave five<br><br>Survey type:<br>• Online survey	Bereavement or specification of type of bereavement:<br>• Lifetime bereavement<br><br>Time since bereavement:<br>• > 6 months<br><br>Elevated grief symptoms:<br>• Not applicable<br><br>Language or writing reading level/proficiency:<br>• Sufficient German skills<br><br>Age criterion:<br>• ≥ 18 years<br><br>Citizenship:<br>• Not applicable<br><br>Residence:<br>• Ukraine<br><br>Nature of death:<br>• Not applicable<br><br>Health status:<br>• Not applicable<br><br>Other:<br>• Not applicable	United Kingdom	United Kingdom and Ireland	None	1771
Siddiqua, 2024 [29]	Target population:<br>• Bereaved individuals of Lahore, Pakistan<br><br>Was the study population described as representative?<br>• No<br><br>Recruitment process:<br>• A convenience sampling technique was used, with participants being recruited	Bereavement or specification of type of bereavement:<br>• Bereavement<br><br>Time ce bereavement:<br>• Not applicable<br><br>Elevated grief symptoms:<br>• Not applicable<br><br>Language or writing reading level/proficiency:	Pakistan	Pakistan and USA	None	737

	through social media websites, mobile apps, and in person by a team of medical students<br><br>Survey type:<br>• Online survey	• Not applicable<br><br>Age criterion:<br>• ≥ 18 years<br><br>Citizenship:<br>• Not applicable<br><br>Residence:<br>• Lahore, Pakistan<br><br>Nature of death:<br>• Bereavement due to COVID-19<br><br>Health status:<br>• Exclusion of participants with a diagnosed mental health disorder e.g. major depression, anxiety, schizophrenia<br><br>Other:<br>• Not applicable				
Singer, 2022 [30]	Target population:<br>• Bereaved samples<br><br>Was the study population described as representative?<br>• No<br><br>Recruitment process:<br>• All three studies were conducted using online surveys<br>• Participants who had experienced bereavement were recruited through Amazon's Mechanical Turk (MTurk) service<br><br>Survey type:<br>• Online survey	Bereavement or specification of type of bereavement:<br>• Loss of a family member or friend<br><br>Time since bereavement:<br>• ≤ 18 months<br><br>Elevated grief symptoms:<br>• Not applicable<br><br>Language or writing reading level/proficiency:<br>• English proficiency<br><br>Age criterion:<br>• ≥ 18 years<br><br>Citizenship:<br>• USA<br><br>Residence:<br>• Not applicable<br><br>Nature of death:<br>• Not applicable<br><br>Health status:<br>• Not applicable<br><br>Other:<br>• Not applicable	USA	USA	None	151

	Target population:<br>• Bereaved samples<br><br>Was the study population described as representative?<br>• No<br><br>Recruitment process:<br>• All three studies were survey studies completed online<br>• Bereaved participants were recruited using Amazon's Mechanical Turk (MTurk) service<br><br>Survey type:<br>• Online survey	Bereavement or specification of type of bereavement:<br>• Death of a parent, child or spouse<br><br>Time since bereavement:<br>• ≤ 18 months<br><br>Elevated grief symptoms:<br>• Not applicable<br><br>Language or writing reading level/proficiency:<br>• English proficiency<br><br>Age criterion:<br>• ≥ 18 years<br><br>Citizenship:<br>• USA<br><br>Residence:<br>• Not applicable<br><br>Nature of death:<br>• Not applicable<br><br>Health status:<br>• Not applicable<br><br>Other:<br>• Not applicable				147
	Target population:<br>• Bereaved samples<br><br>Was the study population described as representative?<br>• No<br><br>Recruitment process:<br>• All three studies were survey studies completed online<br>• Bereaved participants were recruited using Amazon's Mechanical Turk (MTurk) service<br><br>Survey type:<br>• Online survey					302
Specht, 2022 [31]	Target population:<br>• Individuals seeking treatment who filled out an online screening survey to join an internet-based program for post-traumatic stress disorder (PTSD) or depression, offered in Arabic<br><br>Was the study population described as representative?<br>• No<br><br>Recruitment process:<br>• Participants were recruited through internet advertisements, e.g., social media and a program website<br><br>Survey type:	Bereavement or specification of type of bereavement:<br>• Experienced one or more losses of family members or friends<br><br>Time since bereavement:<br>• Not applicable<br><br>Elevated grief symptoms:<br>• Not applicable<br><br>Language or writing reading level/proficiency:<br>• Able to read and write in Arabic<br><br>Age criterion:<br>• ≥ 18 years<br><br>Citizenship:<br>• Not applicable	Germany	Germany	None	1051

	• Online survey	Residence:<br>• Not applicable<br><br>Nature of death:<br>• Not applicable<br><br>Health status:<br>• Not applicable<br><br>Other:<br>• Access to the internet				
Tay, 2019 [32]	Target population:<br>• West Papuan refugees residing in Kiung<br><br>Was the study population described as representative?<br>• Yes<br><br>Recruitment process:<br>• Our sampling frame utilized census data gathered the previous year by the PNG government, which identified all West Papuans living in Kiunga<br>• To update this census, we conducted a house-to-house survey in the nine villages where West Papuans were predominantly located in the Kiunga area<br><br>Survey type:<br>• Interviews	Bereavement or specification of type of bereavement:<br>• Not applicable<br><br>Time since bereavement:<br>• Not applicable<br><br>Elevated grief symptoms:<br>• Not applicable<br><br>Language or writing reading level/proficiency:<br>• Not applicable<br><br>Age criterion:<br>• ≥ 18 years<br><br>Citizenship:<br>• Not applicable<br><br>Residence:<br>• Not applicable<br><br>Nature of death:<br>• Not applicable<br><br>Health status:<br>• Not applicable<br><br>Other:<br>• Born in West Papua or having at least one parent from that region	Papua New Guinea	Australia	None	470
Treml, 2024 [33]	Target population:<br>• Representative sample of the population in Germany<br><br>Was the study population described as representative?<br>• Yes	Bereavement or specification of type of bereavement:<br>• Not applicable<br><br>Time since bereavement:<br>• Not applicable<br><br>Elevated grief symptoms:<br>• Not applicable	Germany	Germany	None	2509

	Recruitment process:<br>• The sample size was determined by the ADM sampling consortium (ADM Arbeitskreis Deutscher Markt- und Sozialforschungsinstitute e.V.), which provides a framework for creating representative samples of private households and their inhabitants<br>• The random sampling process involved three stages:<br><br>1) Random selection of 258 regional sample point areas representing different parts of the country<br>2) Random selection of target households within those sample point areas using the random-route method<br>3) Random selection of one individual within each target household using a Kish-selection grid<br>• The multi-stage sampling design ensured equal probability of household selection<br>• Design weighting was used to equalize selection probabilities within households<br>• Non-response distortions were corrected through weighting adjustments<br>• Distribution at the household level was adjusted to reflect the population, and additional weighting at the individual level corrected biases based on age, gender, and geographic region<br><br>Survey type:<br>• Written questionnaires with interviewers available to provide assistance	Language or writing reading level/proficiency:<br>• Fluent in German<br><br>Age criterion:<br>• ≥ 16 years<br><br>Citizenship:<br>• Not applicable<br><br>Residence:<br>• Not applicable<br><br>Nature of death:<br>• Not applicable<br><br>Health status:<br>• Not applicable<br><br>Other:<br>• Not applicable				
Zhang, 2020 [34]	Target population:<br>• Shidu parents<br><br>Was the study population described as representative?<br>• No<br><br>Recruitment process:<br>• We collected altogether 800 Shidu families from 11 communities of a district in Shanghai by stratified random sampling and selected one parent from each Shidu family as the participant<br>• Of them, 155 Shidu parents met the inclusion criteria	Bereavement or specification of type of bereavement:<br>• Loss of child<br><br>Time since bereavement:<br>• ≥ 1 year<br><br>Elevated grief symptoms:<br>• Not applicable<br><br>Language or writing reading level/proficiency:<br>• Not applicable<br><br>Age criterion:<br>• Both Shidu parents : > 49 years old	China	China	None	149

	• To update the census, we conducted a house-to-house survey, focusing on the nine villages where West Papuans were concentrated in the Kiunga area<br>• Local social workers who already knew and supported the Shidu families assisted the research by providing a brief description of the study during their routine support group meetings or home visits<br><br>Survey type:<br>• Interview	Citizenship:<br>• Not applicable<br><br>Residence:<br>• Not applicable<br><br>Nature of death:<br>• Not applicable<br><br>Health status:<br>• Individuals with serious mental disorders (e.g., schizophrenia, Alzheimer's disease, intellectual disability) were excluded from the study<br><br>Other:<br>• Only one biological child<br>• No adoption of a child by the time of study participation				
Zhou, 2020 [35]	Target population:<br>• Shidu parents<br><br>Was the study population described as representative?<br>• No<br><br>Recruitment process:<br>• Participants were recruited through collaborating community institutes, support groups, public interest organizations, and government agencies across various geographic regions of China<br><br>Survey type:<br>• Written questionnaire filled out by participants or staff after reading questions out loud	Bereavement or specification of type of bereavement:<br>• Loss of an only child<br><br>Time since bereavement:<br>• ≥ 6 months<br><br>Elevated grief symptoms:<br>• Not applicable<br><br>Language or writing reading level/proficiency:<br>• Not applicable<br><br>Age criterion:<br>• Both Shidu parents : > 49 years old<br><br>Citizenship:<br>• Not applicable<br><br>Residence:<br>• Not applicable<br><br>Nature of death:<br>• Not applicable<br><br>Health status:<br>• Participants with severe mental or physical illnesses that prevented them from completing the survey were excluded<br><br>Other:<br>• No biological or adopted child at the time of the survey	China	China, Switzerland and USA	None	1024

Table 3: Items 1a-b, 5, and 7b-j of “Table 3: Elements of the utilized data extraction tool for every included study”

**First author, public-ation year**	**Sample characteristics**											
	**Location of Data Collec-tion (Nation)**	**Location of affiliated institutio n (Nation)**	**Potential added specifi-cation**	**Portion of female study partici-pants in percent**	**Portion of male study partici-pants in percent**	**Portion of diverse/ gender non-confor-ming partici-pants in percent**	**Mean age in years**	**Standard deviation of age**	**Educational level of study participants**	**Mean time in months since death at time of assessm ent**	**Relation-ship to the deceased - Deceased is…**	**Portion of study participants bereaved through a violent death/unnatu -ral in percent**	**Portion of study participants bereaved through a non-violent/ natural death**

Aoun, 2020 [1]	Australia	Australia	None	73	27	0	63.5	12.3	• No formal education: 0.8%<br>• Below primary school: -<br>• Primary school: 2.4%<br>• Completion of primary school: -<br>• No formal education/ Primary school: -<br>• Secondary education: 30.7%<br>• Completion of secondary education: 35.6%<br>• Lower than tertiary education: -<br>• Lower than College/ University degree: -<br>• College/ University education: -<br>• College/ University degree: 30.5%<br>• Other: -	21.6	• Spouse/ partner: 73.7%<br>• Child: 4.2%<br>• Parent: 18.9%<br>• Sibling: 1.8%<br>• Cousins: -<br>• Grandparent: -<br>• 1st degree relative/ Close family: -<br>• Another relative: -<br>• Someone other than a partner or a child: -<br>• Friend/other: 1.3%<br>• Friend/ other relative: -<br>• Other: -<br>• No response: -	0	100
Ben-Ezra, 2020 [2]	African Cross-national	Israel, Ireland, Scotland, Switzer-land and Northern Ireland	None	49.6	50.4	No infor-mation	30.45	N/A	• No formal education: -<br>• Below primary school: -<br>• Primary school: -<br>• Completion of primary school: -<br>• No formal education/ Primary school: 0.2%<br>• Secondary education: 7.8%<br>• Completion of secondary education: -<br>• Lower than tertiary education: -<br>• Lower than College/ University degree: -<br>• College/ University education: 91.9%<br>• College/ University degree: -<br>• Other: -	No infor-mation	No information	No information	No information

	Nigeria			49.8	50.2		30.15	8.72	• No formal education: -<br>• Below primary school: -<br>• Primary school: -<br>• Completion of primary school: -<br>• No formal education/ Primary school: 0.1%<br>• Secondary education: 8.3%<br>• Completion of secondary education: -<br>• Lower than tertiary education: -<br>• Lower than College/ University degree: -<br>• College/ University education: 91.7%<br>• College/ University degree: -<br>• Other: -				
	Kenya			49.1	50.9		32.23	9.36	• No formal education: -<br>• Below primary school: -<br>• Primary school: -<br>• Completion of primary school: -<br>• No formal education/ Primary school: 0.1%<br>• Secondary education: 6%<br>• Completion of secondary education: -<br>• Lower than tertiary education: -<br>• Lower than College/ University degree: -<br>• College/ University education: 93.9%<br>• College/ University degree: -<br>• Other: -				

	Ghana			50	50		28.96	7.93	• No formal education: -<br>• Below primary school: -<br>• Primary school: -<br>• Completion of primary school: -<br>• No formal education/ Primary school: 0.8%<br>• Secondary education: 10.8%<br>• Completion of secondary education: -<br>• Lower than tertiary education: -<br>• Lower than College/ University degree: -<br>• College/ University education: -<br>• College/ University degree: 88.4%<br>• Other: -				
Boelen, 2020 [3]	Nether-lands	Nether-lands	None	83	17	No infor-mation	43.82	14.78	N/A	46.47	• Spouse/ partner: 44.9%<br>• Child: 15.9%<br>• Parent: -<br>• Sibling: -<br>• Cousins: -<br>• Grandparent: -<br>• 1st degree relative/ Close family: -<br>• Another relative: 38.9%<br>• Someone other than a partner or a child: -<br>• Friend/other: -<br>• Friend/ other relative: -<br>• Other: -<br>• No response: -	20	80

Boelen, 2018 [4]	Nether-lands	Nether-lands and Australia	Study popu-lation at time point 1	76.2	23.8	No infor-mation	53.83	13.92	• No formal education: -<br>• Below primary school: -<br>• Primary school: -<br>• Completion of primary school: -<br>• No formal education/ Primary school: -<br>• Secondary education: -<br>• Completion of secondary education: -<br>• Lower than tertiary education: 60.0%<br>• Lower than College/ University degree: -<br>• College/ University education: 39.5%<br>• College/ University degree: -<br>• Other: -	28.64	• Spouse/partner: 66.0%<br>• Child: 10.7%<br>• Parent: -<br>• Sibling: -<br>• Cousins: -<br>• Grandparent: -<br>• 1st degree relative/ Close family: -<br>• Another relative: -<br>• Someone other than a partner or a child: 23.2%<br>• Friend/other: -<br>• Friend/ other relative: -<br>• Other: -<br>• No response: -	13.3	86.7
			Study popu-lation at time point 2	No infor-mation	No infor-mation	No infor-mation	No infor-mation	No infor-mation	No information	25.65	No information	No information	No information

Boelen, 2019 [5]	No infor-mation	Nether-lands	None	90.9	9.1	No infor-mation	41.8	12.5	• No formal education: -<br>• Below primary school: -<br>• Primary school: -<br>• Completion of primary school: -<br>• No formal education/ Primary school: -<br>• Secondary education: -<br>• Completion of secondary education: -<br>• Lower than tertiary education: 44.6%<br>• Lower than College/University degree: -<br>• College/ University education: 54.6%<br>• College/University degree: -<br>• Other: -	42.4	• Spouse/ partner: 31.4%<br>• Child: 19.1%<br>• Parent: -<br>• Sibling: -<br>• Cousins: -<br>• Grandparent: -<br>• 1st degree relative/ Close family: -<br>• Another relative: -<br>• Someone other than a partner or a child: 49.5%<br>• Friend/other: -<br>• Friend/other relative: -<br>• Other: -<br>• No response: -	17.2	82.2
Boelen, Spuji, 2019 [6]	No infor-mation	Nether-lands	None	56.4	43.6	No infor-mation	11.7	2.7	No information	34.6	• Spouse/partner: -<br>• Child: -<br>• Parent: 81.8%<br>• Sibling: 8.9%<br>• Cousins: -<br>• Grandparent: -<br>• 1st degree relative/Close family: -<br>• Another relative: 7.9%<br>• Someone other than a partner or a child: -<br>• Friend/other: -<br>• Friend/other relative: -<br>• Other: -<br>• No response: -	22.3	72.8

Bonanno, 2020 [7]	No information	USA	Study population at time point 1	66	34	No information	55.3	7.2	• No formal education: -<br>• Below primary school: -<br>• Primary school: -<br>• Completion of primary school: -<br>• No formal education/ Primary school: -<br>• Secondary education: -<br>• Completion of secondary education: -<br>• Lower than tertiary education: -<br>• Lower than College/ University degree: 39.2%<br>• College/ University education: -<br>• College/ University degree: 60.8%<br>• Other: -	2.67	• Spouse/partner: 100%<br>• Child: -<br>• Parent: -<br>• Sibling: -<br>• Cousins: -<br>• Grandparent: -<br>• 1st degree relative/ Close family: -<br>• Another relative: -<br>• Someone other than a partner or a child: -<br>• Friend/other: -<br>• Friend/other relative: -<br>• Other: -<br>• No response: -	No information	No information
		USA	Study population at time point 2							14.25	• Spouse/partner: 100%<br>• Child: -<br>• Parent: -<br>• Sibling: -<br>• Cousins: -<br>• Grandparent: -<br>• 1st degree relative/ Close family: -<br>• Another relative: -<br>• Someone other than a partner or a child: -<br>• Friend/other: -<br>• Friend/other relative: -<br>• Other: -<br>• No response: -	No information	No information
		USA	Study population at time point 3	No information	No information	No information	No information	No information	No information	24.92	• Spouse/partner: 100%<br>• Child: -<br>• Parent: -<br>• Sibling: -<br>• Cousins: -<br>• Grandparent: -<br>• 1st degree relative/ Close family: -<br>• Another relative: -<br>• Someone other than a partner or a child: -<br>• Friend/other: -<br>• Friend/ other relative: -<br>• Other: -<br>• No response: -	No information	No information
Eberle, 2023 [8]	Switzerland	Switzerland	None	84.6	15.4	No information	26.89	12.08	• No formal education: -<br>• Below primary school: -<br>• Primary school: 1.8%	No information	No information	No information	No information

									• Completion of primary school: -<br>• No formal education/ Primary school: -<br>• Secondary education: 76.7%<br>• Completion of secondary education: -<br>• Lower than tertiary education: -<br>• Lower than College/ University degree: -<br>• College/ University education: 19.9%<br>• College/University degree: -<br>• Other: 1.6%				
Eisma, 2023 [9]	Nether-lands	Netherl-ands	None	92	8	No infor-mation	52.76	13.96	• No formal education: -<br>• Below primary school: -<br>• Primary school: 0%<br>• Completion of primary school: -<br>• No formal education/ Primary school: -<br>• Secondary education: 46%<br>• Completion of secondary education: -<br>• Lower than tertiary education: -<br>• Lower than College/University degree: -<br>• College/ University education: 54%<br>• College/University degree: -<br>• Other: -	33	• Spouse/partner: 46%<br>• Child: 13%<br>• Parent: 27%<br>• Sibling: 10%<br>• Cousins: -<br>• Grandparent: -<br>• 1st degree relative/Close family: -<br>• Another relative: -<br>• Someone other than a partner or a child: -<br>• Friend/other: -<br>• Friend/other relative: -<br>• Other: 5%<br>• No response: -	25	75
Hane-veld, 2022 [10]	Germany	Germany	None	80.3	19.7	No infor-mation	52.6	13	• No formal education: -<br>• Below primary school: -<br>• Primary school: -<br>• Completion of primary school: -<br>• No formal education/ Primary school: -<br>• Secondary education: -<br>• Completion of secondary education: 59%<br>• Lower than tertiary education: -<br>• Lower than College/University degree: -<br>• College/ University education: -<br>• College/University degree: -	43.7	• Spouse/partner: 39.9%<br>• Child: 21.8%<br>• Parent: 26.4%<br>• Sibling: -<br>• Cousins: -<br>• Grandparent: -<br>• 1st degree relative/Close family: -<br>• Another relative: -<br>• Someone other than a partner or a child: -<br>• Friend/other: -<br>• Friend/other relative: -<br>• Other: 11.9%<br>• No response: -	21.8	77.7

									• Other: -				
Hyland, 2024 [11]	Republic of Ireland	Ireland and United Kingdom	Irish study population	No information	No information	No information	No information	No information	No information	No information	No information	No information	No information
	United Kingdom		UK study population										
Killi-kelly, 2023 [12]	Cross-national	Switzerland, Canada, Greece, Germany, USA, Iran and Turkey	Cross-national sample	76	24	No information	23.87	8.68	• No formal education: -<br>• Below primary school: -<br>• Primary school: -<br>• Completion of primary school: -<br>• No formal education/ Primary school: -<br>• Secondary education: -<br>• Completion of secondary education: 63.4%<br>• Lower than tertiary education: -<br>• Lower than College/University degree: -<br>• College/ University education:-<br>• College/University degree: 35.6%<br>• Other: 0.1%	No information	• Spouse/partner: -<br>• Child: -<br>• Parent: -<br>• Sibling: -<br>• Cousins: -<br>• Grandparent: 50.4 %<br>• 1st degree relative/Close family: 17.1 %<br>• Another relative: -<br>• Someone other than a partner or a child: -<br>• Friend/other: -<br>• Friend/other relative: 32.5 %<br>• Other: -<br>• No response: -	20.9	78.9
	Greece		Greek sample	No information	No information	No information	No information	No information	No information	No information	No information	No information	No information
	Cyprus		Cypriot sample										
	Turkey		Turkish sample										
	Iran		Iranian sample										
	USA		American sample										

Killi-kelly, 2019 [13]	Israel	Switzer-land and Israel	None	54.8	45.2	0	41.4	14.5	• No formal education: -<br>• Below primary school: -<br>• Primary school: 2.4%<br>• Completion of primary school: -<br>• No formal education/ Primary school: -<br>• Secondary education: 28.7%<br>• Completion of secondary education: -<br>• Lower than tertiary education: -<br>• Lower than College/University degree: -<br>• College/ University education: 68.6%<br>• College/University degree: -<br>• Other: 0.4%	No infor-mation	No information	No information	No information
Kokou-Kpolou, 2020 [14]	France	France, Canada and USA	French popu-lation	83.6	16.4	0	53.64	7.27	• No formal education: -<br>• Below primary school: -<br>• Primary school: 0%<br>• Completion of primary school: -<br>• No formal education/ Primary school: -<br>• Secondary education: 58.9%<br>• Completion of secondary education: -<br>• Lower than tertiary education: -<br>• Lower than College/University degree: -<br>• College/ University education: 41.1%<br>• College/University degree: -<br>• Other: -	268.32	• Spouse/partner: 100%<br>• Child: -<br>• Parent: -<br>• Sibling: -<br>• Cousins: -<br>• Grandparent: -<br>• 1st degree relative/Close family: -<br>• Another relative: -<br>• Someone other than a partner or a child: -<br>• Friend/other: -<br>• Friend/other relative: -<br>• Other: -<br>• No response: -	65.75	34.25
	Togo		Togolese popu-lation	88.3	11.7	0	56.04	11.69	Education<br>• No formal education: -<br>• Below primary school: -<br>• Primary school: 69.1%<br>• Completion of primary school: -<br>• No formal education/ Primary school: -<br>• Secondary education: 30.9%<br>• Completion of secondary education: -<br>• Lower than tertiary education: -	112.56	• Spouse/partner: 100%<br>• Child: -<br>• Parent: -<br>• Sibling: -<br>• Cousins: -<br>• Grandparent: -<br>• 1st degree relative/Close family: -<br>• Another relative: -<br>• Someone other than a partner or a child: -<br>• Friend/other: -<br>• Friend/other relative: -	17.28	82.72

									• Lower than College/University degree: -<br>• College/ University education: 0%<br>• College/University degree: -<br>• Other: -		• Other: -<br>• No response: -		
Lenfer-ink, 2022 [15]	Nether-lands	Nether-lands	None	75	25	0	51.9	12.93	• No formal education: -<br>• Below primary school: -<br>• Primary school: -<br>• Completion of primary school: -<br>• No formal education/ Primary school: -<br>• Secondary education: -<br>• Completion of secondary education: -<br>• Lower than tertiary education: 58%<br>• Lower than College/University degree: -<br>• College/ University education: 42%<br>• College/University degree: -<br>• Other: -	57.48	• Spouse/partner: 22%<br>• Child: 39%<br>• Parent: 14%<br>• Sibling: 17%<br>• Cousins: -<br>• Grandparent: -<br>• 1st degree relative/Close family: -<br>• Another relative: -<br>• Someone other than a partner or a child: -<br>• Friend/other: -<br>• Friend/other relative: -<br>• Other: 9%<br>• No response: -	100	0
Lenfer-ink, 2023 [16]	Germany	Germany and Nether-lands	German study popu-lation	81.5	18.5	0	44.8	16.9	• No formal education: -<br>• Below primary school: -<br>• Primary school: 0.5%<br>• Completion of primary school: -<br>• No formal education/ Primary school: -<br>• Secondary education: 54.9%<br>• Completion of secondary education: -<br>• Lower than tertiary education: -<br>• Lower than College/University degree: -<br>• College/ University education: 44.6%<br>• College/University degree: -<br>• Other: -	86.4	• Spouse/partner: 24.8%<br>• Child: 15.8%<br>• Parent: 30.2%<br>• Sibling: 5.4%<br>• Cousins: -<br>• Grandparent: 15.3%<br>• 1st degree relative/Close family: -<br>• Another relative: -<br>• Someone other than a partner or a child: -<br>• Friend/other: 8.6%<br>• Friend/other relative: -<br>• Other: -<br>• No response: -	24.9	76.1
	Nether-lands		Dutch study popu-lation	81	19	0	41.3	16.7	• No formal education: -<br>• Below primary school: -<br>• Primary school: 0.9%<br>• Completion of primary school: -<br>• No formal education/ Primary school: -<br>• Secondary education: 43.6%	63.36	• Spouse/partner: 30.3%<br>• Child: 7.6%<br>• Parent: 29.9%<br>• Sibling: 1.9%<br>• Cousins: -<br>• Grandparent: 18.5%<br>• 1st degree relative/Close family: -	21.8	78.2

									• Completion of secondary education: -<br>• Lower than tertiary education: -<br>• Lower than College/University degree: -<br>• College/ University education: 55.5%<br>• College/University degree: -<br>• Other: -		• Another relative: -<br>• Someone other than a partner or a child: -<br>• Friend/other: 11.8%<br>• Friend/other relative: -<br>• Other: -<br>• No response: -		
Lenfer-ink, van Dijk, 2024 [17]	Sweden	Nether-lands, Sweden and Norway	None	80.2	19.8	0	46.89	10.19	• No formal education: -<br>• Below primary school: -<br>• Primary school: -<br>• Completion of primary school: -<br>• No formal education/ Primary school: -<br>• Secondary education: -<br>• Completion of secondary education: -<br>• Lower than tertiary education: 44.4%<br>• Lower than College/University degree: -<br>• College/ University education: 55.6%<br>• College/University degree: -<br>• Other: -	57.93	• Spouse/partner: -<br>• Child: 100%<br>• Parent: -<br>• Sibling: -<br>• Cousins: -<br>• Grandparent: -<br>• 1st degree relative/Close family: -<br>• Another relative: -<br>• Someone other than a partner or a child: -<br>• Friend/other: -<br>• Friend/other relative: -<br>• Other: -<br>• No response: -	18.6	57.7
Lun-dorff, 2021 [18]	Denmark	Denmark	Study popu-lation at time point 1	70	30	0	70.4	9.7	• No formal education: -<br>• Below primary school: -<br>• Primary school: 56.8%<br>• Completion of primary school: -<br>• No formal education/ Primary school: -<br>• Secondary education: 39.8%<br>• Completion of secondary education: -<br>• Lower than tertiary education: -<br>• Lower than College/University degree: -<br>• College/ University education: -<br>• College/University degree: -<br>• Other: -	2 (time point)	No information	2.7	73.1

			Study population at time point 2	No information	No information	No information	No information	No information	No information	6 (time point)	No information	No information	No information
			Study population at time point 3							11 (time point)			
Redican, 2023 [19]	Denmark	United Kingdom and Denmark	Study population at time point 4	67.6	32.4	No information	62.69	14.29	No information	48 (time point)	• Spouse/partner: - 72.8%<br>• Child: -<br>• Parent: -<br>• Sibling: -<br>• Cousins: -<br>• Grandparent: -<br>• 1st degree relative/ Close family: -<br>• Another relative: -<br>• Someone other than a partner or a child: -<br>• Friend/other: -<br>• Friend/other relative: -<br>• Other: 27.2%<br>• No response: -	No information	No information
Maciejewski, 2016 [20]	USA	USA, Switzerland and Netherlands	None	73.7	26.3	No information	61.8	18.7	• No formal education: -<br>• Below primary school: -<br>• Primary school: -<br>• Completion of primary school: -<br>• No formal education/ Primary school: -<br>• Secondary education: 39.6%<br>• Completion of secondary education: -<br>• Lower than tertiary education: -<br>• Lower than College/ University degree: -<br>• College/ University education: 60.4%<br>• College/University degree: -<br>• Other: -	No information	• Spouse/partner: 83.9%<br>• Child: -<br>• Parent: -<br>• Sibling: -<br>• Cousins: -<br>• Grandparent: -<br>• 1st degree relative/Close family: -<br>• Another relative: -<br>• Someone other than a partner or a child: -<br>• Friend/other: -<br>• Friend/other relative: -<br>• Other: 16.1%<br>• No response: -	No information	No information
Matthews, 2019 [21]	Australia	Australia	Australian study population	No information	No information	No information	No information	No information	No information	No information	No information	No information	No information

	Canada		Can-adian study population										
	USA		American study population										
	United Kingdom		UK study population										
	Total Cohort		Entire study population	89.86	10.14		48.56	12.13		76.8	• Spouse/partner: 38.51%<br>• Child: 35.13%<br>• Parent: 10.15%<br>• Sibling: 14.86%<br>• Cousins: 1.35%<br>• Grandparent: -<br>• 1st degree relative/Close family: -<br>• Another relative: -<br>• Someone other than a partner or a child: -<br>• Friend/other: -<br>• Friend/other relative: -<br>• Other: -<br>• No response: -	100	0
Mauro, 2019 [22]	USA	USA	Study population with impairing grief	78.5	21.5	No information	52	14.5	• No formal education: -<br>• Below primary school: -<br>• Primary school: -<br>• Completion of primary school: -<br>• No formal education/ Primary school: -<br>• Secondary education: -<br>• Secondary education or less: 10.7%<br>• Completion of secondary education: -<br>• Lower than tertiary education: -<br>• Lower than College/ University degree: -<br>• College/ University education: 34.9%%<br>• College/University degree: 54.4%<br>• Other: -	25.2	• Spouse/partner: 36.4%<br>• Child: 29.1%<br>• Parent: 19.2%<br>• Sibling:<br>• Cousins: -<br>• Grandparent: -<br>• 1st degree relative/Close family: -<br>• Another relative: -<br>• Someone other than a partner or a child: -<br>• Friend/other: -<br>• Friend/other relative: 15.3%<br>• Other: -<br>• No response: -	33.3	66.7
			Study population	69.8	30.2	No information	61.3	22.5	No information	154.8	• Spouse/partner: 17.4%<br>• Child: 44.2%<br>• Parent: 7.0%	10.5	89.5

			without impairing grief								• Sibling: -<br>• Cousins: -<br>• Grandparent: -<br>• 1st degree relative/Close family: -<br>• Another relative: -<br>• Someone other than a partner or a child: -<br>• Friend/other: -<br>• Friend/other relative: 31.4%<br>• Other: -<br>• No response: -		
Nielsen, 2020 [23]	Denmark	Denmark	Study population at time point 1	70	30	0	61.8	No information	No relevant information	6 (time point)	• Spouse/partner: 65%<br>• Child: -<br>• Parent: -<br>• Sibling: -<br>• Cousins: -<br>• Grandparent: -<br>• 1st degree relative/Close family: -<br>• Another relative: -<br>• Someone other than a partner or a child: -<br>• Friend/other: -<br>• Friend/other relative: -<br>• Other: 35%<br>• No response: -	0	100
			Study population at time point 2	70	30	0	61.8	No information	No relevant information	36 (time point)	• Spouse/partner: 65%<br>• Child: -<br>• Parent: -<br>• Sibling: -<br>• Cousins: -<br>• Grandparent: -<br>• 1st degree relative/Close family: -<br>• Another relative: -<br>• Someone other than a partner or a child: -<br>• Friend/other: -<br>• Friend/other relative: -<br>• Other: 35%<br>• No response: -	0	100
O'Connor, 2019 [24]	Denmark	Denmark and Netherlands	Study population at time point 1	59	41	No information	72.5	4.2	No information	6 (time point)	• Spouse/partner: 100%<br>• Child: -<br>• Parent: -<br>• Sibling: -<br>• Cousins: -<br>• Grandparent: -<br>• 1st degree	No information	No information

											relative/Close family: -<br>• Another relative: -<br>• Someone other than a partner or a child: -<br>• Friend/other: -<br>• Friend/other relative: -<br>• Other: -<br>• No response: -		
Pein-ado, 2024 [25]	Spain	Spain and United Kingdom	Repre-sentative national sample	No infor-mation	No infor-mation	No infor-mation	No infor-mation	No infor-mation	No information	No infor-mation	No information	No information	No information
			Berea-ved individ-uals from repre-sentative national sample	47.9	52.1	No infor-mation	46.73	12.49	• No formal education: -<br>• Below primary school: -<br>• Primary school: -<br>• Completion of primary school: -<br>• No formal education/ Primary school: 3.7%<br>• Secondary education: 46.7%<br>• Completion of secondary education: -<br>• Lower than tertiary education: -<br>• Lower than College/University degree: -<br>• College/ University education: -<br>• College/University degree: 49.6%<br>• Other: -	No infor-mation	No information	No information	No information
Redi-can, 2024 [26]	Ukraine	United Kingdom, Ireland, Ukraine and Israel	None	48.3	51.7	No infor-mation	No infor-mation	No infor-mation	• No formal education: -<br>• Below primary school: -<br>• Primary school: -<br>• Completion of primary school: 2.8%<br>• No formal education/ Primary school: -<br>• Secondary education: -<br>• Completion of secondary education: 39.3%<br>• Lower than tertiary education: -<br>• Lower than College/University degree: -<br>• College/ University education: -<br>• College/University degree: 57.9%	No info-rmation	No information	19	81

									• Other: -				
Rosner, 2021 [27]	Germany	Germany	None	53.2	46.8	No information	48.2	17.81	• No formal education: -<br>• Below primary school: -<br>• Primary school: 2.4%<br>• Completion of primary school: -<br>• No formal education/ Primary school: -<br>• Secondary education: 84.4%<br>• Completion of secondary education:<br>• Lower than tertiary education: -<br>• Lower than College/University degree: -<br>• College/ University education: 9.9%<br>• College/University degree: -<br>• Other: -	105	• Spouse/partner: 17.4%<br>• Child: 3.5%<br>• Parent: 40.9%<br>• Sibling: -<br>• Cousins: -<br>• Grandparent: -<br>• 1st degree relative/Close family: -<br>• Another relative: -<br>• Someone other than a partner or a child: -<br>• Friend/other: -<br>• Friend/other relative: 37.7%<br>• Other: -<br>• No response: -	No information	No information
Shevlin, 2023 [28]	United Kingdom	United Kingdom and Ireland	None	49.9	50.1	0	No information	No information	No information	No information	No information	No information	No information
Siddiqua, 2024 [29]	Pakistan	Pakistan and USA	None	33.1	66.9	No information	No information	No information	Education<br>• No formal education: -<br>• Below primary school: -<br>• Primary school: -<br>• Completion of primary school: -<br>• No formal education/ Primary school: -<br>• Secondary education: 43.3%<br>• Completion of secondary education: 34.1%<br>• Lower than tertiary education: -<br>• Lower than College/University degree: -<br>• College/ University education: -<br>• College/University degree: 12.6%<br>• Other: -	No information	• Spouse/partner: 13.9%<br>• Child: -<br>• Parent: 32.6%<br>• Sibling: 13.6%<br>• Cousins: -<br>• Grandparent: -<br>• 1st degree relative/Close family: -<br>• Another relative: -<br>• Someone other than a partner or a child: -<br>• Friend/other: -<br>• Friend/other relative: 40.4%<br>• Other: -<br>• No response: -	0	100
Singer, 2022 [30]	USA	USA	Study population 1	58.9	41.1	No information	34.84	No information	• No formal education: -<br>• Below primary school: -<br>• Primary school: -<br>• Completion of primary school: -	5.14	• Spouse/partner: -<br>• Child: -<br>• Parent: 94.2%<br>• Sibling: -<br>• Cousins: -	No information	No information

									• No formal education/ Primary school: -<br>• Secondary education: -<br>• Completion of secondary education: -<br>• Lower than tertiary education: -<br>• Lower than College/University degree: -<br>• College/ University education: -<br>• College/University degree: 30.82%<br>• Other: -		• Grandparent: -<br>• 1st degree relative/Close family: -<br>• Another relative: -<br>• Someone other than a partner or a child: -<br>• Friend/other: -<br>• Friend/other relative: -<br>• Other: 5.8%<br>• No response: -		
			Study popu-lation 2	61.2	38.8	No infor-mation	37.5	No infor-mation	• No formal education: -<br>• Below primary school: -<br>• Primary school: -<br>• Completion of primary school: -<br>• No formal education/ Primary school: -<br>• Secondary education: -<br>• Completion of secondary education: -<br>• Lower than tertiary education: -<br>• Lower than College/University degree: -<br>• College/ University education: -<br>• College/University degree: 40%<br>• Other: -	5.5	• Spouse/partner: -<br>• Child: -<br>• Parent: 78.3%<br>• Sibling: -<br>• Cousins: -<br>• Grandparent: -<br>• 1st degree relative/Close family: -<br>• Another relative: -<br>• Someone other than a partner or a child: -<br>• Friend/other: -<br>• Friend/other relative: -<br>• Other: -<br>• No response: -	No information	No information
			Study popu-lation 3	58.3	41.7	No infor-mation	37.31	No infor-mation	• No formal education: -<br>• Below primary school: -<br>• Primary school: -<br>• Completion of primary school: -<br>• No formal education/ Primary school: -<br>• Secondary education: -<br>• Completion of secondary education: -<br>• Lower than tertiary education: -<br>• Lower than College/University degree: -<br>• College/ University education: -<br>• College/University degree:	5.77	• Spouse/partner: -<br>• Child: -<br>• Parent: 62%<br>• Sibling: -<br>• Cousins: -<br>• Grandparent: -<br>• 1st degree relative/Close family: -<br>• Another relative: -<br>• Someone other than a partner or a child: -<br>• Friend/other: -<br>• Friend/other relative: -<br>• Other: -<br>• No response: -	No information	No information

									50%<br>• Other: -				
Specht, 2022 [31]	Germany	Germany	None	73.5	26.5	0	25.4	7.52	• No formal education: -<br>• Below primary school: -<br>• Primary school: -<br>• Completion of primary school: -<br>• No formal education/ Primary school: -<br>• Secondary education: -<br>• Completion of secondary education: -<br>• Lower than tertiary education: -<br>• Lower than College/University degree: 59.8%<br>• College/ University education: -<br>• College/University degree: 40.2%<br>• Other: -	73.5	• Spouse/partner: -<br>• Child: -<br>• Parent: -<br>• Sibling: -<br>• Cousins: -<br>• Grandparent: 34.6%<br>• 1st degree relative/Close family: 37.2%<br>• Another relative: -<br>• Someone other than a partner or a child: -<br>• Friend/other: -<br>• Friend/other relative: 28.2%<br>• Other: -<br>• No response: -	17.6	82.4
Tay, 2019 [32]	Papua New Guinea	Australia	None	44.1	55.9	No infor-mation	35.8	0.65	• No formal education: -<br>• Below primary school: -<br>• Primary school: -<br>• Completion of primary school: 46.6%<br>• No formal education/ Primary school: -<br>• Secondary education: 9.5%<br>• Completion of secondary education: -<br>• Lower than tertiary education: -<br>• Lower than College/University degree: -<br>• College/ University education: -<br>• College/University degree: -<br>• Other: -	No infor-mation	No information	No information	No information
Treml, 2024 [33]	Germany	Germany	None	50.9	49	0.1	49.48	17.81	• No formal education: -<br>• Below primary school: -<br>• Primary school: 26.7%<br>• Completion of primary school: -<br>• No formal education/ Primary school: -<br>• Secondary education: 49.0%<br>• Completion of secondary education: -<br>• Lower than tertiary education:	102.88	• Spouse/partner: 17.4%<br>• Child: 2.6%<br>• Parent: 40.9%<br>• Sibling: -<br>• Cousins: -<br>• Grandparent: -<br>• 1st degree relative/Close family: -<br>• Another relative: -<br>• Someone other than a partner or a child: -	11	89

									-<br>• Lower than College/University degree: -<br>• College/ University education: 22.5%<br>• College/University degree: -<br>• Other: 1.8%		• Friend/other: -<br>• Friend/other relative: 39.1%<br>• Other: -<br>• No response: -		
Zhang, 2020 [34]	China	China	None	60.4	39.6	No infor-mation	62.25	4.88	• No formal education: -<br>• Below primary school: -<br>• Primary school: 49.7%<br>• Completion of primary school: -<br>• No formal education/ Primary school: -<br>• Secondary education: 36.9%<br>• Completion of secondary education: -<br>• Lower than tertiary education: -<br>• Lower than College/University degree: -<br>• College/ University education: 13.4%<br>• College/University degree: -<br>• Other: -	134.16	• Spouse/partner: -<br>• Child: 100%<br>• Parent: -<br>• Sibling: -<br>• Cousins: -<br>• Grandparent: -<br>• 1st degree relative/Close family: -<br>• Another relative: -<br>• Someone other than a partner or a child: -<br>• Friend/other: -<br>• Friend/other relative: -<br>• Other: -<br>• No response: -	28.2	71.8
Zhou, 2020 [35]	China	China, Switzer-land and USA	None	62.4	37	No infor-mation	59.91	7.26	• No formal education: -<br>• Below primary school: 24.4%<br>• Primary school: -<br>• Completion of primary school: -<br>• No formal education/ Primary school: -<br>• Secondary education: -<br>• Completion of secondary education: -<br>• Lower than tertiary education: -<br>• Lower than College/University degree: -<br>• College/ University education: -<br>• College/University degree: -<br>• Other: 65.5%	112.92	• Spouse/partner: -<br>• Child: 100%<br>• Parent: -<br>• Sibling: -<br>• Cousins: -<br>• Grandparent: -<br>• 1st degree relative/Close family: -<br>• Another relative: -<br>• Someone other than a partner or a child: -<br>• Friend/other: -<br>• Friend/other relative: -<br>• Other: -<br>• No response: -	49.8	49.4

Table 4: Items 1a-b and 8-11 of “Table 3: Elements of the utilized data extraction tool for every included study”

**First author, publication year**	**Specification of study population(s)**	**Instrument(s)**	**Diagnostic algorithm**						**Prevalence estimates**	**Longitudinal prevalence estimates**
			**Which diagnostic criteria were applied?**	**For ICD-11 PGD prevalences: Which ICD-11 criteria were fulfilled due to the eligibility criteria or assessment/analysis process?**	**Further relevant specification of diagnostic algorithm**	**Do the applied criteria represent a raised diagnostic threshold for ICD-11?**	**Do the applied criteria represent a lowered diagnostic threshold for ICD-11?**	**For ICD-11 PGD prevalences: Which ICD-11 criteria were omitted?**		
Aoun, 2020 [1]	None	• the Prolonged Grief Disorder (PG-13)	ICD-11 PGD:<br>• Bereavement<br>• Duration Criterion<br>• ≥1 separation distress symptom<br>• ≥1 accompanying symptom<br>• Social/functional impairment	• Bereavement	None	No	Yes	• Culture criterion	8.70% (95% CI: 6.2–11.9%)	N/A
Ben-Ezra, 2020 [2]	African Cross-national	• the Inventory of Complicated Grief-Revised (ICG-R)	ICD-11 PGD:<br>• Bereavement<br>• Duration Criterion<br>• ≥1 separation distress symptom<br>• ≥3 accompanying symptoms<br>• Social/functional impairment	• None	None	Yes	Yes	• Culture criterion	3.70% (95% CI: 3.1-4.5%)	N/A
	Nigeria								4.60% (95% CI: 3.4-6.0%)	
	Kenya								3.40% (95% CI: 2.5-4.8%)	
	Ghana								2.60% (95% CI: 1.5-4.4%)	
Boelen, 2020 [3]	None	• the Inventory of Complicated Grief-Revised (ICG-R)<br>• 15-item depression subscale from the Symptom-Checklist-90 (SCL depression)	ICD-11 PGD:<br>• ≥1 separation distress symptom (symptoms 1–2)<br>• ≥1 accompanying symptom (symptoms 3–12)<br>• Functional impairment item	• Bereavement<br>• Duration criterion	None	No	Yes	• Culture criterion	19.80%	N/A
			PGD 2009:<br>• Endorsement of the separation distress symptom (symptom 1)<br>• ≥5 accompanying symptoms (symptoms 2–10)<br>• Functional impairment item			Yes	Yes		11.20%	

			PGD-BD:<br>• ≥1 separation distress symptom (symptoms 1–2)<br>• ≥1 accompanying symptom (symptoms 3–7)<br>• Functional impairment item			No	Yes		19.20%	
			CG:<br>• ≥1 separation distress symptom (symptoms 1–4)<br>• ≥2 accompanying symptoms (symptoms 5–12)<br>• Functional impairment item			Yes	Yes		21.50%	
Boelen, 2018 [4]	Study population at time point 1	• the Inventory of Complicated Grief-Revised (ICG-R)<br>• Beck depression inventory (BDI-II)	ICD-11 PGD:<br>• ≥ 1 separation distress symptom<br>• ≥ 1 additional symptom<br>• Functional impairment item	• Bereavement<br>• Duration criterion: range of time since loss: 6-120 months	None	No	Yes	• Culture criterion	18%	N/A
	Study population at time point 2			• Bereavement<br>• Duration criterion		No	Yes		8.20%	
Boelen, 2019 [5]	None	• the Inventory of Complicated Grief-Revised (ICG-R)<br>• Symptom-Checklist-90 (SCL-depression)	ICD-11 PGD:<br>• ≥1 separation distress symptom (symptoms 1–2)<br>• ≥1 accompanying symptom (symptoms 3–12)<br>• Functional impairment item	• Bereavement<br>• Duration criterion	None	No	Yes	• Culture criterion	19.20%	N/A
			ICD-11 PGD (with minimum of two accompanying symptoms):<br>• ≥1 separation distress symptom (symptoms 1–2)<br>• ≥2 accompanying symptoms (symptoms 3–12)<br>• Functional impairment item			Yes	Yes		17.60%	
			ICD-11 PGD (with minimum of three accompanying symptoms):<br>• ≥1 separation distress symptom (symptoms 1–2)<br>• ≥3 accompanying symptoms (symptoms 3–12)<br>• Functional impairment item			Yes	Yes		15.40%	

			ICD-11 PGD (with minimum of four accompanying symptoms):<br>• ≥1 separation distress symptom (symptoms 1–2)<br>• ≥4 accompanying symptoms (symptoms 3–12)<br>• Functional impairment item			Yes	Yes		11.10%	
			ICD-11 PGD (with minimum of five accompanying symptoms):<br>• ≥1 separation distress symptom (symptoms 1–2)<br>• ≥5 accompanying symptoms (symptoms 3–12)<br>• Functional impairment item			Yes	Yes		8.30%	
			ICD-11 PGD (with minimum of six accompanying symptoms):<br>• ≥1 separation distress symptom (symptoms 1–2)<br>• ≥6 accompanying symptoms (symptoms 3–12)<br>• Functional impairment item			Yes	Yes		5.30%	
Boelen, Spuji 2019 [6]	None	• the Inventory of prolonged grief for children (IPG-C)<br>• the Inventory of prolonged grief for adolescents (IPG-A)<br>• Child PTSD symptom scale (CPSS)<br>• Children's depression inventory (CDI)	ICD-11 PGD:<br>• ≥ 1 separation distress symptom (symptoms 1–2)<br>• ≥ 1 accompanying symptom (symptoms 3–12)<br>• Functional impairment item	• Bereavement<br>• Duration criterion	None	No	Yes	• Culture criterion	12.40%	N/A
			ICD-11 PGD_less conservative:<br>• ≥ 1 separation distress symptom (symptoms 1–2)<br>• ≥ 1 accompanying symptom (symptoms 3–12)<br>• Functional impairment item		Symptoms considered present when rated with the intermediate or highest response on the three-point frequency-scale.	No	Yes		43.00%	
Bonanno, 2020 [7]	Study population at time point 1	• Self developed Structured Clinical Interview<br>• Global Assessment of Functioning Scale	No prevalence report	• Not applicable	Not applicable	Not applicable	Not applicable	• Not applicable	Not applicable	N/A
	Study population at time point 2		No prevalence report	• Not applicable	Not applicable	Not applicable	Not applicable	• Not applicable	Not applicable	
	Study population at time point 3		ICD-11 PGD:<br>• ≥ 1 separation distress symptom<br>• ≥ 1 accompanying symptom<br>• Functional impairment item	• Bereavement<br>• Duration criterion	None	No	Yes	• Culture criterion	11.7 %	

			ICD-11 PGD (1+3):<br>• ≥ 1 separation distress symptom<br>• ≥ 3 accompanying symptoms<br>• Functional impairment item		None	Yes	Yes		8.80%	
			ICD-11 PGD (1+5):<br>• ≥ 1 separation distress symptom<br>• ≥ 5 accompanying symptoms<br>• Functional impairment item		None	Yes	Yes		4.60%	
Eberle, 2023 [8]	None	• the International Prolonged Grief Disorder Questionnaire (IPGDS)<br>• the Yearning in Situations of Loss – Short Form (YSL-SF)	ICD-11 PGD<br>• Bereavement<br>• Duration Criterion<br>• ≥ 1 separation distress symptom<br>• ≥ 1 accompanying symptom<br>• Functional impairment item<br>• Culture criterion	• None	None	No	No	• None	0.22%	N/A
Eisma, 2023 [9]	None	• the Traumatic Grief Inventory Self-Report Plus (TGI-SR+)	ICD-11 PGD (1+5):<br>• ≥ 1 separation distress symptom<br>• ≥ 5 accompanying symptoms<br>• Functional impairment item	• Bereavement<br>• Duration criterion	None	Yes	Yes	• Culture criterion	28%	N/A
Haneveld, 2022 [10]	None	• the extended translation of the PG-13 (PG-13+9)	ICD-11 PGD:<br>• Bereavement<br>• Duration criterion<br>• ≥ 1 separation distress symptom<br>• ≥ 1 accompanying symptom<br>• Functional impairment item	• Bereavement<br>• Duration criterion	None	No	Yes	• Culture criterion	76%	N/A
			ICD-11 PGD (12 months):<br>• Bereavement<br>• Duration criterion<br>• ≥ 1 separation distress symptom<br>• ≥ 1 accompanying symptom<br>• Functional impairment item	• Bereavement	Changed duration criterion to 12 months.	Yes	Yes	• Culture criterion	57%	
Hyland, 2024 [11]	Irish study population	• the International Grief Questionnaire (IGQ)	IGQ ICD-11 PGD:<br>• Bereavement<br>• Duration criterion<br>• ≥ 1 separation distress symptom<br>• ≥ 1 accompanying symptom<br>• Functional impairment item<br>• Cultural criterion	• Bereavement		No	No	• None	10.90% (95% CI: 9.0-12.9%)	N/A

		• the International Prolonged Grief Disorder Questionnaire (IPGDS)	IPGDS ICD-11 PGD (strict criteria):<br>• Bereavement<br>• Duration criterion<br>• ≥ 1 separation distress symptom<br>• ≥ 1 accompanying symptom<br>• Functional impairment item<br>• Cultural criterion		On the 0-4 scale the severity of separation distress symptom(s), the accompanying symptom(s) and the functional impairment must be rated as 4 ("always") or higher.	No	No	• None	9.60%	N/A
			IPGDS ICD-11 PGD (moderate criteria):<br>• Bereavement<br>• Duration criterion<br>• ≥ 1 separation distress symptom<br>• ≥ 1 accompanying symptom<br>• Functional impairment item<br>• Cultural criterion		On the 0-4 scale the severity of separation distress symptom(s), the accompanying symptom(s) and the functional impairment must be rated as 3 ("often") or higher.	No	Yes	• None	14.30%	
	UK study population	• The International Grief Questionnaire (IGQ)	IGQ ICD-11 PGD:<br>• Bereavement<br>• Duration criterion<br>• ≥ 1 separation distress symptom<br>• ≥ 1 accompanying symptom<br>• Functional impairment item<br>• Cultural criterion			No	No	• None	15.30% (95% CI: 13.0-17.6%)	
		• International Prolonged Grief Disorder Scale (IPGDS)	IPGDS ICD-11 PGD (strict criteria):<br>• Bereavement<br>• Duration criterion<br>• ≥ 1 separation distress symptom<br>• ≥ 1 accompanying symptom<br>• Functional impairment item<br>• Cultural criterion		On the 0-4 scale the severity of separation distress symptom(s), the accompanying symptom(s) and the functional impairment must be rated as 4 ("always") or higher.	No	No	• None	14.40%	
			IPGDS ICD-11 PGD (moderate		On the 0-4 scale	No	Yes	• None	19.40%	

			criteria):<br>• Bereavement<br>• Duration criterion<br>• ≥ 1 separation distress symptom<br>• ≥ 1 accompanying symptom<br>• Functional impairment item<br>• Cultural criterion		the severity of separation distress symptom(s), the accompanying symptom(s) and the functional impairment must be rated as 3 ("often") or higher.					
Killikelly, 2023 [12]	Cross-national	• the Inventory of Complicated Grief-Revised (ICG-R)	ICD-11 PGD:<br>• Bereavement<br>• Duration criterion<br>• ≥ 1 separation distress symptom<br>• ≥ 1 accompanying symptom<br>• Functional impairment item<br>• Culture Criterion	• Bereavement<br>• Duration criterion	Symptoms had to have a score of ≥ 4 point to be considered present.	No	No	• None	3.60%	N/A
			ICD-11 PGD moderate:<br>• Bereavement<br>• Duration criterion<br>• ≥ 1 separation distress symptom<br>• ≥ 1 accompanying symptom<br>• Functional impairment item<br>• Culture Criterion		Symptoms had to have a score of ≥ 3 point to be considered present.	No	Yes		18.00%	
			ICD-11 Maciejewski PGD:<br>• Bereavement<br>• Duration criterion<br>• ≥ 1 separation distress symptom<br>• 3-5 accompanying symptoms<br>• Functional impairment item<br>• Culture Criterion		Symptoms had to have a score of ≥ 4 point to be considered present.	Yes	No		15.20%	
	Greece/Cyprus		ICD-11 PGD:<br>• Bereavement<br>• Duration criterion<br>• ≥ 1 separation distress symptom<br>• ≥ 1 accompanying symptom<br>• Functional impairment item<br>• Culture Criterion		Symptoms had to have a score of ≥ 4 point to be considered present.	No	No		6.90%	
			ICD-11 PGD moderate:<br>• Bereavement<br>• Duration criterion<br>• ≥ 1 separation distress symptom<br>• ≥ 1 accompanying symptom<br>• Functional impairment item<br>• Culture Criterion		Symptoms had to have a score of ≥ 3 point to be considered present.	No	Yes		2.10%	
			ICD-11 Maciejewski PGD:<br>• Bereavement<br>• Duration criterion		Symptoms had to have a score of ≥ 4 point to be	Yes	No		2.90%	

			• ≥ 1 separation distress symptom<br>• 3-5 accompanying symptoms<br>• Functional impairment item<br>• Culture Criterion		considered present.				
	Turkey/Iran		ICD-11 PGD:<br>• Bereavement<br>• Duration criterion<br>• ≥ 1 separation distress symptom<br>• ≥ 1 accompanying symptom<br>• Functional impairment item<br>• Culture Criterion		Symptoms had to have a score of ≥ 4 point to be considered present.	No	No		3.20%
			ICD-11 PGD moderate:<br>• Bereavement<br>• Duration criterion<br>• ≥ 1 separation distress symptom<br>• ≥ 1 accompanying symptom<br>• Functional impairment item<br>• Culture Criterion		Symptoms had to have a score of ≥ 3 point to be considered present.	No	Yes		1.50%
			ICD-11 Maciejewski PGD:<br>• Bereavement<br>• Duration criterion<br>• ≥ 1 separation distress symptom<br>• 3-5 accompanying symptoms<br>• Functional impairment item<br>• Culture Criterion		Symptoms had to have a score of ≥ 4 point to be considered present.	Yes	No		1.40%
	USA		ICD-11 PGD:<br>• Bereavement<br>• Duration criterion<br>• ≥ 1 separation distress symptom<br>• ≥ 1 accompanying symptom<br>• Functional impairment item<br>• Culture Criterion		Symptoms had to have a score of ≥ 4 point to be considered present.	No	No		2.80%
			ICD-11 PGD moderate:<br>• Bereavement<br>• Duration criterion<br>• ≥ 1 separation distress symptom<br>• ≥ 1 accompanying symptom<br>• Functional impairment item<br>• Culture Criterion		Symptoms had to have a score of ≥ 3 point to be considered present.	No	Yes		18.10%
			ICD-11 Maciejewski PGD:<br>• Bereavement<br>• Duration criterion<br>• ≥ 1 separation distress symptom<br>• 3-5 accompanying symptoms<br>• Functional impairment item<br>• Culture Criterion		Symptoms had to have a score of ≥ 4 point to be considered present.	Yes	No		12.10%

Killikelly, 2019 [13]	None	• the Inventory of Complicated Grief-Revised (ICG-R)	ICD-11 PGD:<br>• Bereavement<br>• Duration criterion<br>• ≥ 1 separation distress symptom<br>• ≥ 1 accompanying symptom<br>• Functional impairment item	• Bereavement	None	No	Yes	• Culture criterion	2%	N/A
			ICD-11 PGD variation:<br>• Bereavement<br>• Duration criterion<br>• ≥ 1 separation distress symptom<br>• ≥ 3 accompanying symptoms			Yes	Yes	• Culture criterion<br>• Functional impairment	4.20%	
Kokou-Kpolou, 2020 [14]	French study population	• the Prolonged Grief Scale (PGS)–11	ICD-11 Model 1:<br>• ≥ 1 separation distress symptom<br>• ≥ 5 accompanying symptoms<br>• Functional impairment item	• Bereavement<br>• Duration criterion	None	Yes	Yes	• Culture criterion	21.90%<br>(95% CI:<br>13.0-36.0%)	N/A
			ICD-11 Model 2:<br>• ≥ 1 separation distress symptom<br>• ≥ 3 accompanying symptoms<br>• Functional impairment item			Yes			26%<br>(95% CI:<br>16.0-41.0%)	
	Togolese study population		ICD-11 Model 1:<br>• ≥ 1 separation distress symptom<br>• ≥ 5 accompanying symptoms<br>• Functional impairment item			Yes			15.40%<br>(95% CI:<br>10.0-23.0%)	
			ICD-11 Model 2:<br>• ≥ 1 separation distress symptom<br>• ≥ 3 accompanying symptoms<br>• Functional impairment item			Yes			17.30%<br>(95% CI:<br>12.0-25.0%)	
Lenferink, 2022 [15]	None	• the Traumatic Grief Inventory Self-Report Plus (TGI-SR+)	ICD-11 PGD:<br>• ≥ 1 separation distress symptom<br>• ≥ 1 accompanying symptom<br>• Functional impairment item	• Bereavement<br>• Duration criterion	None	No	Yes	• Culture criterion	33%	N/A

Lenferink, 2023 [16]	German and dutch study population	• the TGICA (modified TGI-SR+)	ICD-11 PGD:<br>• ≥ 1 separation distress symptom<br>• ≥ 1 accompanying symptom<br>• Functional impairment item	• Bereavement<br>• Duration criterion	None	No	Yes	• Culture criterion	6.20%	N/A
			ICD-11 PGD:<br>• ≥ 1 separation distress symptom<br>• ≥ 2 accompanying symptoms<br>• Functional impairment item			Yes	Yes		5.50%	
			ICD-11 PGD:<br>• ≥ 1 separation distress symptom<br>• ≥ 3 accompanying symptoms<br>• Functional impairment item			Yes	Yes		4.60%	
			ICD-11 PGD:<br>• ≥ 1 separation distress symptom<br>• ≥ 4 accompanying symptoms<br>• Functional impairment item			Yes	Yes		3.20%	
			ICD-11 PGD:<br>• ≥ 1 separation distress symptom<br>• ≥ 5 accompanying symptoms<br>• Functional impairment item			Yes	Yes		3.20%	
			ICD-11 PGD:<br>• ≥ 1 separation distress symptom<br>• ≥ 6 accompanying symptoms<br>• Functional impairment item			Yes	Yes		2.50%	
Lenferink, van Dijk, 2024 [17]	None	• the Traumatic Grief Inventory Self-Report Plus (TGI-SR+)	ICD-11 PGD:<br>• ≥ 1 separation distress symptom<br>• ≥ 1 accompanying symptom<br>• Functional impairment item	• Bereavement<br>• Duration criterion	None	No	Yes	• Culture criterion	32%	N/A
			ICD-11 PGD (conservative):<br>• ≥ 1 separation distress symptom<br>• ≥ 5 accompanying symptoms<br>• Functional impairment item			Yes	Yes		26%	
Lundorff, 2021 [18]	Study population at time point 1	• the Prolonged Grief Disorder (PG-13)<br>• the Inventory of Complicated Grief-	ICD-11 PGD:<br>• Bereavement<br>• ≥ 1 separation distress symptom<br>• ≥ 1 accompanying symptom<br>• Functional impairment item	• Bereavement	None	No	Yes	• Culture criterion<br>• Duration criterion	20.90% (95% CI: 18.2-23.8%)	N/A

		Revised (ICG-R)	ICD-11 PGD variation:<br>• Bereavement<br>• ≥ 1 separation distress symptom<br>• ≥ 6 accompanying symptoms			Yes	Yes	• Culture criterion<br>• Functional impairment<br>• Duration criterion	5.80%<br>(95% CI: 4.4-7.7%)	N/A
	Study population at time point 2		ICD-11 PGD:<br>• Bereavement<br>• Duration criterion<br>• ≥ 1 separation distress symptom<br>• ≥ 1 accompanying symptom<br>• Functional impairment item			No	Yes	• Culture criterion	18.90%<br>(95% CI: 16.2-21.8%)	Dual-point prevalence of ICD-11 PGD at time point 1 and 2: 10.4% (95% CI: 8.3-12.8%)
			ICD-11 PGD variation:<br>• Bereavement<br>• Duration criterion<br>• ≥ 1 separation distress symptom<br>• ≥ 6 accompanying symptoms			Yes	Yes	• Culture criterion<br>• Functional impairment	5.30%<br>(95% CI: 3.8-7.1%)	Dual-point prevalence of ICD-11 PGD variation at time point 1 and 2: 3.2% (95% CI: 2.30-4.7%)
	Study population at time point 3		ICD-11 PGD:<br>• Bereavement<br>• Duration criterion<br>• ≥ 1 separation distress symptom<br>• ≥ 1 accompanying symptom<br>• Functional impairment item			No	Yes	• Culture criterion	13.40%<br>(95% CI: 11.0-16.0%)	Dual-point prevalence of ICD-11 PGD at time point 2 and 3: 9.8% (95% CI: 7.7-12.1%)
			ICD-11 PGD variation:<br>• Bereavement<br>• Duration criterion<br>• ≥ 1 separation distress symptom<br>• ≥ 6 accompanying symptoms			Yes	Yes	• Culture criterion<br>• Functional impairment	3.10%<br>(95% CI: 2.0-4.6%)	Dual-point prevalence of ICD-11 PGD variation at time point 2 and 3: 1.8% (95% CI: 1.0-3.0%)
Redican, 2023 [19]	Study population at time point 4	• the Aarhus Prolonged Grief Disorder Scale (A-PGDs)	ICD-11 PGD:<br>• Bereavement<br>• Duration criterion<br>• ≥ 1 separation distress symptom<br>• ≥ 1 accompanying symptom<br>• Functional impairment item<br>• Culture Criterion	• Bereavement	None	No	No	• None	1.50%<br>(95% CI: 0.5-2.6%)	N/A

			ICD-11 PGD:<br>• Bereavement<br>• Duration criterion<br>• ≥ 1 separation distress symptom<br>• ≥ 1 accompanying symptom<br>• Functional impairment item			No	Yes	• Culture Criterion	2.50%<br>(95% CI: 1.2-3.8%)	
Maciejewski, 2016 [20]	None	• the Inventory of Complicated Grief-Revised (ICG-R)<br>• the ICD-11 prolonged grief disorder (ICD-11) test	PGD:<br>• ≥ 1 separation distress symptom<br>• ≥ 5 accompanying symptoms	• Bereavement<br>• Duration criterion	None	Yes	Yes	• Functional impairment criterion<br>• Cultural criterion	11.90%<br>(95% CI: 8.1-15.8%)	N/A
			CG:<br>• ≥ 1 separation distress symptom<br>• ≥ 2 accompanying symptoms			Yes	Yes		30.20%<br>(95% CI: 24.7-35.7%)	
			ICD-11 PGD:<br>• ≥ 1 separation distress symptom<br>• ≥ 3 accompanying symptoms			Yes	Yes		12.70%<br>(95% CI: 8.7-16.7%)	
Matthews, 2019 [21]	Australia study population	• the Prolonged Grief Disorder (PG-13)	ICD-11 PGD:<br>• Bereavement<br>• Duration criterion<br>• ≥ 1 separation distress symptom<br>• ≥ 5 accompanying symptoms<br>• Functional impairment	• Bereavement	None	Yes	Yes	• Culture criterion	62.50%	N/A
	Canadian study population					Yes	Yes		17.19%	
	US study population					Yes	Yes		14.06%	
	UK study population					Yes	Yes		6.25%	
	Entire study population					Yes	Yes		43.20%	
Mauro, 2019 [22]	Study population with impairing grief	• the Structured Clinical Interview for CG (SCI-CG)	ICD-11 PGD variation 1:<br>• ≥ 1 separation distress symptom	• Bereavement<br>• Duration criterion<br>• Functional impairment	None	No	Yes	• Culture criterion<br>• Accompany-ing symptoms	95.80%<br>(95% CI: 93.3–98.2%)	N/A
			ICD-11 PGD:<br>• ≥ 1 separation distress symptom<br>• ≥ 1 accompanying symptom			No		• Culture criterion	95.80%<br>(95% CI: 93.3–98.2%)	
			ICD-11 PGD variation 2:<br>• ≥ 1 separation distress symptom<br>• ≥ 2 accompanying symptoms			Yes			95.00%<br>(95% CI: 92.4–97.7%)	
			ICD-11 PGD variation 3:<br>• ≥ 1 separation distress symptom<br>• ≥ 3 accompanying symptoms			Yes			91.60%<br>(95% CI: 88.2–94.9%)	

			ICD-11 PGD variation 4:<br>• ≥ 1 separation distress symptom<br>• ≥ 4 accompanying symptoms			Yes			86.20%<br>(95% CI:<br>82.0–90.4%)	
			ICD-11 PGD variation 5:<br>• ≥ 1 separation distress symptom<br>• ≥ 5 accompanying symptoms			Yes			72.80%<br>(95% CI:<br>67.4–78.2%)	
			ICD-11 PGD variation 6:<br>• ≥ 1 separation distress symptom<br>• ≥ 6 accompanying symptoms			Yes			45.20%<br>(95% CI:<br>39.2–51.3%)	
			PGDPLOS:<br>• ≥ 1 separation distress symptom<br>• ≥ 5 accompanying symptoms		The accompanying symptoms differed from the ones listed for ICD-11 PGD.	Yes			59%<br>(95% CI:<br>53.0–65.0%)	
	Study population without impairing grief		ICD-11 PGD:<br>• ≥ 1 separation distress symptom<br>• ≥ 1 accompanying symptom	• Bereavement<br>• Duration criterion	None	No	Yes	• Culture criterion<br>• Functional impairment	0%	
			PGDPLOS:<br>• ≥ 1 separation distress symptom<br>• ≥ 5 accompanying symptoms		The accompanying symptoms differed from the ones listed for ICD-11 PGD.	Yes			0%	
Nielsen, 2020 [23]	Study population at time point 1	• the Prolonged Grief Disorder (PG-13)	ICD-11 PGD:<br>• Bereavement<br>• Duration criterion<br>• ≥ 1 separation distress symptom<br>• ≥ 5 accompanying symptoms<br>• Functional impairment	• Bereavement<br>• Duration criterion	None	Yes	Yes	• Culture criterion	7.3%	N/A
	Study population at time point 2								1.9%	
O'Connor, 2019 [24]	None	• the Inventory of Complicated Grief-Revised (ICG-R)	PGD:<br>• ≥ 1 separation distress symptom<br>• ≥ 4 accompanying symptoms	• Bereavement<br>• Duration criterion	None	Yes	Yes	• Culture criterion<br>• Functional	9.20%<br>(95% CI:<br>5.2–13.2%)	N/A

		• the Prolonged grief disorder (PGD) test<br>• the Complicated grief (CG) test<br>• the ICD-11 prolonged grief disorder (ICD-11) test	CG:<br>• ≥ 1 separation distress symptom<br>• ≥ 2 accompanying symptoms			Yes	Yes	impairment	48.10%<br>(95% CI: 41.2–54.9%)	
			ICD-11 PGD:<br>• ≥ 1 separation distress symptom<br>• ≥ 3 accompanying symptoms			Yes	Yes		5.80%<br>(95% CI: 2.6–9.1%)	
Peinado, 2024 [25]	Entire study population	• the International Prolonged Grief Disorder Questionnaire (IPGDS)	ICD-11 PGD:<br>• ≥ 1 separation distress symptom<br>• ≥ 1 accompanying symptom<br>• Functional impairment<br>• Culture criterion	• Bereavement<br>• Duration criterion	None	No	No	• None	9.90%	N/A
	Bereaved Study population								15.70%	
Redican, 2024 [26]	None	• the International Grief Questionnaire (IGQ)	ICD-11 PGD:<br>• Bereavement<br>• Duration criterion<br>• ≥ 1 separation distress symptom<br>• ≥ 1 accompanying symptom<br>• Functional impairment<br>• Culture criterion	• None	None	No	No	• None	11.40%<br>(95% CI: 10.0–12.8%)	N/A
Rosner, 2021 [27]	None	• the extended translation of the PG-13 (PG-13+9)	PGD ICD-11:<br>• Bereavement<br>• Duration criterion<br>• ≥ 1 separation distress symptom<br>• ≥ 1 accompanying symptom<br>• Functional impairment	• None	None	No	Yes	• Culture criterion	1.50%	N/A
			PGD ICD-11 (2+ accessory symptoms):<br>• Bereavement<br>• Duration criterion<br>• ≥ 1 separation distress symptom<br>• ≥ 2 accompanying symptoms<br>• Functional impairment			Yes			1.40%	
			PGD ICD-11 (3+ accessory symptoms):<br>• Bereavement<br>• Duration criterion<br>• ≥ 1 separation distress symptom<br>• ≥ 3 accompanying symptoms<br>• Functional impairment			Yes			1.40%	

			PGD ICD-11 (4+ accessory symptoms):<br>• Bereavement<br>• Duration criterion<br>• ≥ 1 separation distress symptom<br>• ≥ 4 accompanying symptoms<br>• Functional impairment			Yes			1.30%	
			PGD ICD-11 (5+ accessory symptoms):<br>• Bereavement<br>• Duration criterion<br>• ≥ 1 separation distress symptom<br>• ≥ 5 accompanying symptoms<br>• Functional impairment			Yes			1%	
			PGD ICD-11 (6+ accessory symptoms):<br>• Bereavement<br>• Duration criterion<br>• ≥ 1 separation distress symptom<br>• ≥ 6 accompanying symptoms<br>• Functional impairment			Yes			0.70%	
			PGD ICD-11 (7+ accessory symptoms):<br>• Bereavement<br>• Duration criterion<br>• ≥ 1 separation distress symptom<br>• ≥ 7 accompanying symptoms<br>• Functional impairment			Yes			0.50%	
Shevlin, 2023 [28]	None	• the International Prolonged Grief Disorder Questionnaire (IPGDS)	ICD-11 PGD (moderate criteria):<br>• Bereavement<br>• ≥ 1 separation distress symptom<br>• ≥ 1 accompanying symptom<br>• Functional impairment<br>• Culture criterion	• Bereavement<br>• Duration criterion	The diagnostic criteria were met when items were rated ≥ 3 (i.e., 'sometimes', 'often', 'always').	No	Yes	• None	6.90%	N/A
			ICD-11 PGD (strict criteria):<br>• Bereavement<br>• ≥ 1 separation distress symptom<br>• ≥ 1 accompanying symptom<br>• Functional impairment<br>• Culture criterion		The diagnostic criteria were met when items were rated ≥ 4 (i.e., 'often', 'always').	No	No		2.10%	
Siddiqua, 2024 [29]	None	• the Prolonged Grief Disorder (PG-13)	• Bereavement<br>• Duration criterion<br>• ≥ 1 separation distress symptom<br>• ≥ 5 accompanying symptoms<br>• Functional impairment	• Bereavement	None	Yes	Yes	• Culture criterion	15%	N/A
Singer,	Study population	• the Prolonged	• Bereavement	• Bereavement	None	Yes	Yes	• Culture	9.20%	N/A

2022 [30]	1	Grief Disorder (PG-13)	• Duration criterion<br>• ≥ 1 separation distress symptom<br>• ≥ 5 accompanying symptoms<br>• Functional impairment	• Duration criterion				criterion		
	Study population 2								8.10%	
	Study population 3								8.40%	
Specht, 2022 [31]	None	• the Prolonged Grief Disorder (PG-13)	• Bereavement<br>• Duration criterion<br>• ≥ 1 separation distress symptom<br>• ≥ 5 accompanying symptoms<br>• Functional impairment	• Bereavement<br>• Duration criterion	None	Yes	Yes	• Culture criterion	18.80%	N/A
Tay, 2019 [32]	None	• Self developed tool	• Lacks clear description	• Lacks clear description	Lacks clear description	Lacks clear description	Lacks clear description	• Lacks clear description	21.00%	N/A
Treml, 2024 [33]	None	• the Prolonged Grief Disorder (PG-13)	• Bereavement<br>• Duration criterion<br>• ≥ 1 separation distress symptom<br>• ≥ 1 accompanying symptom<br>• Functional impairment	• None	None	No	Yes	• Culture criterion	2.30%	N/A
Zhang, 2020 [34]	None	• the Prolonged Grief Disorder (PG-13)	• Bereavement<br>• Duration criterion<br>• ≥ 1 separation distress symptom<br>• ≥ 5 accompanying symptoms<br>• Functional impairment	• Bereavement<br>• Duration criterion	None	Yes	Yes	• Culture criterion	22.20%	N/A
Zhou, 2020 [35]	None	• the Prolonged Grief Disorder (PG-13)<br>• Depression subscale of the Symptom Checklist-90 (SCL-depression	• Bereavement<br>• Duration criterion<br>• ≥ 1 separation distress symptom<br>• ≥ 1 accompanying symptom<br>• Functional impairment	• Bereavement<br>• Duration criterion	None	No	Yes	• Culture criterion	35.50%	N/A

References

1. Aoun SM, Kissane DW, Cafarella PA, Rumbold B, Hogden A, Jiang L, Bear N. Grief, depression, and anxiety in bereaved caregivers of people with motor neurone disease: a population-based national study. Amyotroph Lateral Scler Front Degener. 2020 Oct 1;21(7–8):593–605.

2. Ben-Ezra M, Hyland P, Karatzias T, Maercker A, Hamama-Raz Y, Lavenda O, Mahat-Shamir M, Shevlin M. A cross-country psychiatric screening of ICD-11 disorders specifically associated with stress in Kenya, Nigeria and Ghana. Eur J Psychotraumatology. 2020 Feb 13;11(1):1720972.

3. Boelen PA, Lenferink LIM. Comparison of six proposed diagnostic criteria sets for disturbed grief. Psychiatry Res. 2020 Mar;285:112786.

4. Boelen PA, Lenferink LIM, Nickerson A, Smid GE. Evaluation of the factor structure, prevalence, and validity of disturbed grief in DSM-5 and ICD-11. J Affect Disord. 2018 Nov;240:79–87.

5. Boelen PA, Lenferink LIM, Smid GE. Further evaluation of the factor structure, prevalence, and concurrent validity of DSM-5 criteria for Persistent Complex Bereavement Disorder and ICD-11 criteria for Prolonged Grief Disorder. Psychiatry Res. 2019 Mar;273:206–10.

6. Boelen PA, Spuij M, Lenferink LIM. Comparison of DSM-5 criteria for persistent complex bereavement disorder and ICD-11 criteria for prolonged grief disorder in help-seeking bereaved children. J Affect Disord. 2019 May;250:71–8.

7. Bonanno GA, Malgaroli M. Trajectories of grief: Comparing symptoms from the *DSM-5* and ICD-11 diagnoses. Depress Anxiety. 2020 Jan;37(1):17–25.

8. Eberle DJ, Maercker A. Stress-associated symptoms and disorders: A transdiagnostic comparison. Clin Psychol Psychother. 2023 Sep;30(5):1047–57.

9. Eisma MC, Lenferink LIM. Co-occurrence of approach and avoidance in prolonged grief: a latent class analysis. Eur J Psychotraumatology. 2023 Nov 23;14(2):2190544.

10. Haneveld J, Rosner R, Vogel A, Kersting A, Rief W, Steil R, Comtesse H. Same name, same content? Evaluation of DSM-5-TR and ICD-11 prolonged grief criteria. J Consult Clin Psychol. 2022 Apr;90(4):303–13.

11. Hyland P, Redican E, Karatzias T, Shevlin M. The International Grief Questionnaire (IGQ): A new measure of *ICD-11* prolonged grief disorder. J Trauma Stress. 2024 Feb;37(1):141–53.

12. Killikelly C, Kagialis A, Henneman S, Coronado H, Demanarig D, Farahani H, Özdoğru AA, Yalçın B, Yockey A, Gosnell CL, Jia F, Maisel M, Stelzer E, Wilson D, Anderson J, Charles K, Cummings JP, Faas C, Knapp B, Koneczny B, Koch C, Bauer LM, Cuccolo C, Edlund JE, Heermans GF, McGillivray S, Shane-Simpson C, Staples A, Zheng Z, Zlokovich MS, Irgens MS. Measurement and assessment of grief in a large international sample. J Affect Disord. 2023 Apr;327:306–14.

13. Killikelly C, Lorenz L, Bauer S, Mahat-Shamir M, Ben-Ezra M, Maercker A. Prolonged grief disorder: Its co-occurrence with adjustment disorder and post-traumatic stress disorder in a bereaved Israeli general-population sample. J Affect Disord. 2019 Apr;249:307–14.

14. Kokou-Kpolou CK, Cénat JM, Noorishad PG, Park S, Bacqué MF. A comparison of prevalence and risk factor profiles of prolonged grief disorder among French and Togolese bereaved adults. Soc Psychiatry Psychiatr Epidemiol. 2020 Jun;55(6):757–64.

15. Lenferink LIM, Eisma MC, Smid GE, De Keijser J, Boelen PA. Valid measurement of DSM-5 persistent complex bereavement disorder and DSM-5-TR and ICD-11 prolonged grief disorder: The Traumatic Grief Inventory-Self Report Plus (TGI-SR+). Compr Psychiatry. 2022 Jan;112:152281.

16. Lenferink LIM, Franzen M, Ten Klooster PM, Knaevelsrud C, Boelen PA, Heeke C. The Traumatic Grief Inventory-Clinician Administered: A psychometric evaluation of a new interview for ICD-11 and DSM-5-TR prolonged grief disorder severity and probable caseness. J Affect Disord. 2023 Jun;330:188–97.

17. Lenferink LIM, Van Dijk I, Eisma MC, Eklund R, Boelen PA, Sveen J. Psychometric evaluation of the Swedish Traumatic Grief Inventory Self-Report Plus (TGI-SR+) in bereaved parents. Clin Psychol Psychother. 2024 Jan;31(1):e2922.

18. Lundorff M, Johannsen M, O'Connor M. Time elapsed since loss or grief persistency? Prevalence and predictors of ICD-11 prolonged grief disorder using different applications of the duration criterion. J Affect Disord. 2021 Jan;279:89–97.

19. Redican E, Vang ML, Komischke-Konnerup K, Elklit A, Shevlin M, O'Connor M. Operationalization, implications and correlates of the cultural deviance criterion for ICD-11 and DSM-5 prolonged grief disorder. Death Stud. 2023 Dec 26;1–12.

20. Maciejewski PK, Maercker A, Boelen PA, Prigerson HG. "Prolonged grief disorder" and "persistent complex bereavement disorder", but not "complicated grief", are one and the same diagnostic entity: an analysis of data from the Yale Bereavement Study. World Psychiatry. 2016 Oct;15(3):266–75.

21. Matthews LR, Quinlan MG, Bohle P. Posttraumatic Stress Disorder, Depression, and Prolonged Grief Disorder in Families Bereaved by a Traumatic Workplace Death: The Need for Satisfactory Information and Support. Front Psychiatry. 2019 Aug 30;10:609.

22. Mauro C, Reynolds CF, Maercker A, Skritskaya N, Simon N, Zisook S, Lebowitz B, Cozza SJ, Shear MK. Prolonged grief disorder: clinical utility of ICD-11 diagnostic guidelines. Psychol Med. 2019 Apr;49(5):861–7.

23. Nielsen MK, Christensen KS, Neergaard MA, Bidstrup PE, Guldin MB. Exploring Functional Impairment in Light of Prolonged Grief Disorder: A Prospective, Population-Based Cohort Study. Front Psychiatry. 2020 Dec 9;11:537674.

24. O'Connor M, Lasgaard M, Larsen L, Johannsen M, Lundorff M, Farver-Vestergaard I, Boelen PA. Comparison of proposed diagnostic criteria for pathological grief using a sample of elderly bereaved spouses in Denmark: Perspectives on future bereavement research. J Affect Disord. 2019 May;251:52–9.

25. Peinado V, Valiente C, Contreras A, Trucharte A, Butter S, Murphy J, Shevlin M. ICD -11 prolonged grief disorder: Prevalence, predictors, and co-occurrence in a large representative sample. Int J Psychol. 2024 Feb;59(1):86–95.

26. Redican E, Hyland P, Shevlin M, Martsenkovskyi D, Karatzias T, Ben-Ezra M. Prevalence and correlates of ICD -11 prolonged grief disorder among adults living in Ukraine during the war with Russia. Acta Psychiatr Scand. 2024 May;149(5):425–35.

27. Rosner R, Comtesse H, Vogel A, Doering BK. Prevalence of prolonged grief disorder. J Affect Disord. 2021 May;287:301–7.

28. Shevlin M, Redican E, Hyland P, Murphy J, Karatzias T, McBride O, Bennett K, Butter S, Hartman TK, Vallières F, Bentall RP. Symptoms and levels of ICD-11 Prolonged Grief Disorder in a representative community sample of UK adults. Soc Psychiatry Psychiatr Epidemiol. 2023 Oct;58(10):1535–47.

29. Siddiqua A, Ahmad S, Nawaz I, Zeeshan M, Rao A. The smoldering pandemic: Prevalence of prolonged grief disorder. A cross-sectional study of bereaving adults during the COVID pandemic in Pakistan. Asian J Psychiatry. 2024 Jan;91:103870.

30. Singer J, McLean E, Kahler J, Papa A. An evaluation of common risk factors for prolonged grief disorder using the international classification of diseases-11 criteria. Aging Ment Health. 2022 Nov 2;26(11):2202–7.

31. Specht F, Vöhringer M, Knaevelsrud C, Wagner B, Stammel N, Böttche M. Prolonged grief disorder in Arabic-speaking treatment-seeking populations: Relationship with socio-demographic aspects, loss- and trauma-related characteristics, and mental health support. Front Psychiatry. 2022 Sep 15;13:933848.

32. Tay AK, Mohsin M, Rees S, Tam N, Kareth M, Silove D. The structure and psychosocial correlates of complicated bereavement amongst refugees from West Papua. Soc Psychiatry Psychiatr Epidemiol. 2019 Jun;54(6):771–80.

33. Treml J, Linde K, Brähler E, Kersting A. Prolonged grief disorder in ICD-11 and DSM-5-TR: differences in prevalence and diagnostic criteria. Front Psychiatry. 2024 Feb 8;15:1266132.

34. Zhang H, Shang Z, Wu L, Sun Z, Zhang F, Sun L, Zhou Y, Wang Y, Liu W. Prolonged grief disorder in Chinese Shidu parents who have lost their only child. Eur J Psychotraumatology. 2020 Dec 31;11(1):1726071.

35. Zhou N, Wen J, Stelzer EM, Killikelly C, Yu W, Xu X, Shi G, Luo H, Wang J, Maercker A. Prevalence and associated factors of prolonged grief disorder in Chinese parents bereaved by losing their only child. Psychiatry Res. 2020 Feb;284:112766.

Table 5: Demographic characteristics of study samples by geographic origin

**Continent**	**Country**	**Proportion of female participants**	**Mean age of participants**	**Mean time since loss at the time of assessment in months**	**Proportion of unnatural deaths**
**Asia**	China	60.4 - 62.4	61.08	123.54	28.2-49.8
	Iran/ Turkey	No information	No information	No information	No information
	Israel	54.8	41.4	No information	No information
	Pakistan	33.1	No information	No information	0
**Africa**	Ghana	50	28.96	No information	No information
	Kenya	49.1	32.23	No information	No information
	Nigeria	49.8	30.15	No information	No information
	Tongo	88.3	56.04	112.56	17.28
**Europe**	Cyprus/ Greece	No information	No information	No information	No information
	Denmark	59-70	62.69	6	0-2.7

	France	83.6	53.64	268.32	65.75
	Germany/ Netherlands	50.9-92	48.2	45.09	11-100
	Spain	No information	No information	No information	No information
	Sweden	80.2	46.89	57.93	18.6
	Switzerland	84.6	26.89	No information	No information
	Ukraine	48.3	No information	No information	19
	United Kingdom	49.9	No information	No information	No information
	Republic of Ireland	No information	No information	No information	No information
**North America**	Canada	No information	No information	No information	No information
	USA	58.3-78.5	52	15.35	10.5-33.3
**Oceania**	Australia	73	63.5	21.6	0
	Papua New Guinea	44.1	35.8	No information	No information

Supplement G

Table 1A: Prevalence estimates from the category "general population"

Category of study population	Application of ICD-11 criteria	Mean time since bereavement/ Time point of assessments post-loss in months	Geographic Origin	Prevalence	Publication
GP	PGD assessed according to the ICD-11	No information	Spain	9.90%	[25] Peinado, 2024
	PGD assessed according to the ICD-11	No information	Ukraine	11.40% (95% CI: 10.0–12.8%)	[26] Redican, 2024
	Lowered diagnostic threshold	102.88	Germany/Netherlands	2.30%	[33] Treml, 2024
	Lowered diagnostic threshold	105	Germany/Netherlands	1.50%	[27] Rosner, 2021
	Combined raised and lowered diagnostic threshold	105	Germany/Netherlands	1.40%	[27] Rosner, 2021
	Combined raised and lowered diagnostic threshold	105	Germany/Netherlands	1.40%	[27] Rosner, 2021
	Combined raised and lowered diagnostic threshold	105	Germany/Netherlands	1.30%	[27] Rosner, 2021
	Combined raised and lowered diagnostic threshold	105	Germany/Netherlands	1%	[27] Rosner, 2021
	Combined raised and lowered diagnostic threshold	105	Germany/Netherlands	0.70%	[27] Rosner, 2021
	Combined raised and lowered diagnostic threshold	105	Germany/Netherlands	0.50%	[27] Rosner, 2021
	Combined raised and lowered diagnostic threshold	No information	Ghana	2.60% (95% CI: 1.5-4.4%)	[2] Ben-Ezra, 2020
	Combined raised and lowered diagnostic threshold	No information	Kenya	3.40% (95% CI: 2.5-4.8%)	[2] Ben-Ezra, 2020
	Combined raised and lowered diagnostic threshold	No information	Nigeria	4.60% (95% CI: 3.4-6.0%)	[2] Ben-Ezra, 2020

Table 1B: Mean, IQR, Q1 and Q3 for the category "general population" for all estimates and divided by application of ICD-11 criteria

Category of study population	ICD-11 Application	Q2	Q1	Q3	IQR
GP	All ICD-11 Applications	1.50	1.15	4	2.85
	Combined raised and lowered diagnostic threshold	1.40	0.85	3	2.15

Table 2A: Prevalence estimates for the category "bereaved adults"

Category of study population	Application of ICD-11 criteria	Mean time since bereavement/ Time point of assessments post-loss in months	Geographic Origin	Prevalence	Publication
BA	PGD assessed according to the ICD-11	48	Denmark	1.50% (95% CI: 0.5-2.6%)	[19] Redican, 2023
	PGD assessed according to the ICD-11	No information	Iran/Turkey	3.20%	[12] Killikelly, 2023
	PGD assessed according to the ICD-11	No information	Cyprus/Greece	6.90%	[12] Killikelly, 2023
	PGD assessed according to the ICD-11	No information	Republic of Ireland	10.90% (95% CI: 9.0-12.9%)	[11] Hyland, 2024
	PGD assessed according to the ICD-11	No information	Republic of Ireland	9.60%	[11] Hyland, 2024
	PGD assessed according to the ICD-11	No information	United Kingdom	15.30% (95% CI: 13.0-17.6%)	[11] Hyland, 2024
	PGD assessed according to the ICD-11	No information	United Kingdom	14.40%	[11] Hyland, 2024
	PGD assessed according to the ICD-11	No information	United Kingdom	2.10%	[28] Shevlin, 2023
	PGD assessed according to the ICD-11	No information	USA	2.80%	[12] Killikelly, 2023
	Lowered diagnostic threshold	2	Denmark	20.90% (95% CI: 18.2-23.8%)	[18] Lundorff, 2021
	Lowered diagnostic threshold	6	Denmark	18.90% (95% CI: 16.2-21.8%)	[18] Lundorff, 2021
	Lowered diagnostic threshold	11	Denmark	13.40% (95% CI: 11.0-16.0%)	[18] Lundorff, 2021
	Lowered diagnostic threshold	24.92	USA	11.7%	[7] Bonanno, 2020
	Lowered diagnostic threshold	25.65	Germany/Netherlands	8.20%	[4] Boelen, 2018
	Lowered diagnostic threshold	28.64	Germany/Netherlands	18%	[4] Boelen, 2018
	Lowered diagnostic threshold	46.47	Germany/Netherlands	19.80%	[3] Boelen, 2020
	Lowered diagnostic threshold	46.47	Germany/Netherlands	19.20%	[3] Boelen, 2020

Table 2B: Mean, IQR, Q1 and Q3 for the category "bereaved adults" for all estimates and divided by application of ICD-11 criteria

Category of study population	ICD-11 Application	Q2	Q1	Q3	IQR

BA	All ICD-11 Applications	9.2	3.7	17.245	13.545
	PGD assessed according to the ICD-11	6.90	2.45	12.65	10.2
	Lowered diagnostic threshold	13.85	6.2	19.2	13
	Combined raised and lowered diagnostic threshold	9	4.6	17.19	12.59

Table 3A: Prevalence estimates from the category "bereaved adults with impairing/intense grief"

Category of study population	Application of ICD-11 criteria	Mean time since bereavement/ Time point of assessments post-loss in months	Geographic Origin	Prevalence	Publication
BA_IG	Lowered diagnostic threshold	25.2	USA	95.80% (95% CI: 93.3–98.2%)	[22] Mauro, 2019
	Lowered diagnostic threshold	25.2	USA	95.80% (95% CI: 93.3–98.2%)	[22] Mauro, 2019
	Lowered diagnostic threshold	42.4	Germany/ Netherlands	19.20%	[5] Boelen, 2019
	Lowered diagnostic threshold	43.7	Germany/ Netherlands	76%	[10] Haneveld, 2022
	Combined raised and lowered diagnostic threshold	25.2	USA	95.00% (95% CI: 92.4–97.7%)	[22] Mauro, 2019
	Combined raised and lowered diagnostic threshold	25.2	USA	91.60% (95% CI: 88.2–94.9%)	[22] Mauro, 2019
	Combined raised and lowered diagnostic threshold	25.2	USA	86.20% (95% CI: 82.0–90.4%)	[22] Mauro, 2019
	Combined raised and lowered diagnostic threshold	25.2	USA	72.80% (95% CI: 67.4–78.2%)	[22] Mauro, 2019
	Combined raised and lowered diagnostic threshold	25.2	USA	45.20% (95% CI: 39.2–51.3%)	[22] Mauro, 2019
	Combined raised and lowered diagnostic threshold	25.2	USA	59% (95% CI: 53.0–65.0%)	[22] Mauro, 2019
	Combined raised and lowered diagnostic threshold	42.4	Germany/ Netherlands	17.60%	[5] Boelen, 2019
	Combined raised and lowered diagnostic threshold	42.4	Germany/ Netherlands	15.40%	[5] Boelen, 2019
	Combined raised and lowered diagnostic threshold	42.4	Germany/ Netherlands	11.10%	[5] Boelen, 2019

	Combined raised and lowered diagnostic threshold	42.4	Germany/ Netherlands	8.30%	[5] Boelen, 2019
	Combined raised and lowered diagnostic threshold	42.4	Germany/ Netherlands	5.30%	[5] Boelen, 2019
	Combined raised and lowered diagnostic threshold	43.7	Germany/ Netherlands	57%	[10] Haneveld, 2022
	Combined raised and lowered diagnostic threshold	73.5	Germany/ Netherlands	18.80%	[31] Specht, 2022

Table 3B: Mean, IQR, Q1 and Q3 for the category "bereaved adults with impairing/intense grief" for all estimates and divided by application of ICD-11 criteria

Category of study population	**ICD-11 Application**	**Q2**	**Q1**	**Q3**	**IQR**
BA_IG	All ICD-11 Applications	57	16.5	88.9	72.4
	Lowered diagnostic threshold	85.9	47.6	95.8	48.2
	Combined raised and lowered diagnostic threshold	45.2	13.25	79.5	66.25

Table 4: Prevalence estimates from the category "bereaved caregivers"

**Category of study population**	**Application of ICD-11 criteria**	**Mean time since bereavement/ Time point of assessments post-loss in months**	**Geographic Origin**	**Prevalence**	**Publication**
BC	Lowered diagnostic threshold	21.6	Australia	8.70% (95% CI: 6.2–11.9%)	[1] Aoun, 2020
	Combined raised and lowered diagnostic threshold	6	Denmark	7.30%	[23] Nielsen, 2020
	Combined raised and lowered diagnostic threshold	36	Denmark	1.90%	[23] Nielsen, 2020

Table 5A: Prevalence estimates from the category "bereaved parents"

**Category of study population**	**Application of ICD-11 criteria**	**Mean time since bereavement/ Time point of assessments post-loss in months**	**Geographic Origin**	**Prevalence**	**Publication**
BP	Lowered diagnostic threshold	57.93	Sweden	32%	[17] Lenferink, van Dijk, 2024
	Lowered diagnostic threshold	57.93	Sweden	26%	[17] Lenferink, van Dijk, 2024
	Combined raised and lowered diagnostic threshold	112.92	China	22.20%	[34] Zhang, 2020
	Combined raised and lowered diagnostic threshold	134.16	China	35.50%	[35] Zhou, 2020

Table 5B: Mean, IQR, Q1 and Q3 for the category "bereaved parents" for all estimates and divided by application of ICD-11 criteria

Category of study population	ICD-11 Application	Q2	Q1	Q3	IQR
BP	All ICD-11 Applications	29	24.1	33.75	9.65

Table 6: Prevalence estimates from the category "bereaved children and adolescents"

Category of study population	Application of ICD-11 criteria	Mean time since bereavement/ Time point of assessments post-loss in months	Geographic Origin	Prevalence	Publication
BCA	Lowered diagnostic threshold	34.6	Germany/ Netherlands	12.40%	[6] Boelen, Spuji 2019
	Lowered diagnostic threshold	34.6	Germany/ Netherlands	43.00%	[6] Boelen, Spuji 2019

Table 7: Prevalence estimates for the category "non-representative community adults"

Category of study population	Application of ICD-11 criteria	Mean time since bereavement/ Time point of assessments post-loss in months	Geographic Origin	Prevalence	Publication
NRCA	PGD assessed according to the ICD-11	No information	Switzerland	0.22%	[8] Eberle, 2023
	Lacks clear description	No information	Papua New Guinea	21%	[32] Tay, 2019

References

1. Aoun SM, Kissane DW, Cafarella PA, Rumbold B, Hogden A, Jiang L, Bear N. Grief, depression, and anxiety in bereaved caregivers of people with motor neurone disease: a population-based national study. Amyotroph Lasteral Scler Front Degener. 2020 Oct 1;21(7–8):593–605.
2. Ben-Ezra M, Hyland P, Karatzias T, Maercker A, Hamama-Raz Y, Lavenda O, Mahat-Shamir M, Shevlin M. A cross-country psychiatric screening of ICD-11 disorders specifically associated with stress in Kenya, Nigeria and Ghana. Eur J Psychotraumatology. 2020 Feb 13;11(1):1720972.
3. Boelen PA, Lenferink LIM. Comparison of six proposed diagnostic criteria sets for disturbed grief. Psychiatry Res. 2020 Mar;285:112786.
4. Boelen PA, Lenferink LIM, Nickerson A, Smid GE. Evaluation of the factor structure, prevalence, and validity of disturbed grief in DSM-5 and ICD-11. J Affect Disord. 2018 Nov;240:79–87.
5. Boelen PA, Lenferink LIM, Smid GE. Further evaluation of the factor structure, prevalence, and concurrent validity of DSM-5 criteria for Persistent Complex Bereavement Disorder and ICD-11 criteria for Prolonged Grief Disorder. Psychiatry Res. 2019 Mar;273:206–10.
6. Boelen PA, Spuij M, Lenferink LIM. Comparison of DSM-5 criteria for persistent complex bereavement disorder and ICD-11 criteria for prolonged grief disorder in help-seeking bereaved children. J Affect Disord. 2019 May;250:71–8.
7. Bonanno GA, Malgaroli M. Trajectories of grief: Comparing symptoms from the *DSM-5* and ICD-11 diagnoses. Depress Anxiety. 2020 Jan;37(1):17–25.
8. Eberle DJ, Maercker A. Stress-associated symptoms and disorders: A transdiagnostic comparison. Clin Psychol Psychother. 2023 Sep;30(5):1047–57.
9. Eisma MC, Lenferink LIM. Co-occurrence of approach and avoidance in prolonged grief: a latent class analysis. Eur J Psychotraumatology. 2023 Nov 23;14(2):2190544.

10. Haneveld J, Rosner R, Vogel A, Kersting A, Rief W, Steil R, Comtesse H. Same name, same content? Evaluation of DSM-5-TR and ICD-11 prolonged grief criteria. J Consult Clin Psychol. 2022 Apr;90(4):303–13.
11. Hyland P, Redican E, Karatzias T, Shevlin M. The International Grief Questionnaire (IGQ): A new measure of *ICD-11* prolonged grief disorder. J Trauma Stress. 2024 Feb;37(1):141–53.
12. Killikelly C, Kagialis A, Henneman S, Coronado H, Demanarig D, Farahani H, Özdoğru AA, Yalçın B, Yockey A, Gosnell CL, Jia F, Maisel M, Stelzer E, Wilson D, Anderson J, Charles K, Cummings JP, Faas C, Knapp B, Koneczny B, Koch C, Bauer LM, Cuccolo C, Edlund JE, Heermans GF, McGillivray S, Shane-Simpson C, Staples A, Zheng Z, Zlokovich MS, Irgens MS. Measurement and assessment of grief in a large international sample. J Affect Disord. 2023 Apr;327:306–14.
13. Killikelly C, Lorenz L, Bauer S, Mahat-Shamir M, Ben-Ezra M, Maercker A. Prolonged grief disorder: Its co-occurrence with adjustment disorder and post-traumatic stress disorder in a bereaved Israeli general-population sample. J Affect Disord. 2019 Apr;249:307–14.
14. Kokou-Kpolou CK, Cénat JM, Noorishad PG, Park S, Bacqué MF. A comparison of prevalence and risk factor profiles of prolonged grief disorder among French and Togolese bereaved adults. Soc Psychiatry Psychiatr Epidemiol. 2020 Jun;55(6):757–64.
15. Lenferink LIM, Eisma MC, Smid GE, De Keijser J, Boelen PA. Valid measurement of DSM-5 persistent complex bereavement disorder and DSM-5-TR and ICD-11 prolonged grief disorder: The Traumatic Grief Inventory-Self Report Plus (TGI-SR+). Compr Psychiatry. 2022 Jan;112:152281.
16. Lenferink LIM, Franzen M, Ten Klooster PM, Knaevelsrud C, Boelen PA, Heeke C. The Traumatic Grief Inventory-Clinician Administered: A psychometric evaluation of a new interview for ICD-11 and DSM-5-TR prolonged grief disorder severity and probable caseness. J Affect Disord. 2023 Jun;330:188–97.
17. Lenferink LIM, Van Dijk I, Eisma MC, Eklund R, Boelen PA, Sveen J. Psychometric evaluation of the Swedish Traumatic Grief Inventory Self-Report Plus (TGI-SR+) in bereaved parents. Clin Psychol Psychother. 2024 Jan;31(1):e2922.
18. Lundorff M, Johannsen M, O'Connor M. Time elapsed since loss or grief persistency? Prevalence and predictors of ICD-11 prolonged grief disorder using different applications of the duration criterion. J Affect Disord. 2021 Jan;279:89–97.
19. Redican E, Vang ML, Komischke-Konnerup K, Elklit A, Shevlin M, O'Connor M. Operationalization, implications and correlates of the cultural deviance criterion for ICD-11 and DSM-5 prolonged grief disorder. Death Stud. 2023 Dec 26;1–12.
20. Maciejewski PK, Maercker A, Boelen PA, Prigerson HG. "Prolonged grief disorder" and "persistent complex bereavement disorder", but not "complicated grief", are one and the same diagnostic entity: an analysis of data from the Yale Bereavement Study. World Psychiatry. 2016 Oct;15(3):266–75.
21. Matthews LR, Quinlan MG, Bohle P. Posttraumatic Stress Disorder, Depression, and Prolonged Grief Disorder in Families Bereaved by a Traumatic Workplace Death: The Need for Satisfactory Information and Support. Front Psychiatry. 2019 Aug 30;10:609.
22. Mauro C, Reynolds CF, Maercker A, Skritskaya N, Simon N, Zisook S, Lebowitz B, Cozza SJ, Shear MK. Prolonged grief disorder: clinical utility of ICD-11 diagnostic guidelines. Psychol Med. 2019 Apr;49(5):861–7.
23. Nielsen MK, Christensen KS, Neergaard MA, Bidstrup PE, Guldin MB. Exploring Functional Impairment in Light of Prolonged Grief Disorder: A Prospective, Population-Based Cohort Study. Front Psychiatry. 2020 Dec 9;11:537674.
24. O'Connor M, Lasgaard M, Larsen L, Johannsen M, Lundorff M, Farver-Vestergaard I, Boelen PA. Comparison of proposed diagnostic criteria for pathological grief using a sample of elderly bereaved spouses in Denmark: Perspectives on future bereavement research. J Affect Disord. 2019 May;251:52–9.
25. Peinado V, Valiente C, Contreras A, Trucharte A, Butter S, Murphy J, Shevlin M. ICD -11 prolonged grief disorder: Prevalence, predictors, and co-occurrence in a large representative sample. Int J Psychol. 2024 Feb;59(1):86–95.
26. Redican E, Hyland P, Shevlin M, Martsenkovskyi D, Karatzias T, Ben-Ezra M. Prevalence and correlates of ICD -11 prolonged grief disorder among adults living in Ukraine during the war with Russia. Acta Psychiatr Scand. 2024 May;149(5):425–35.
27. Rosner R, Comtesse H, Vogel A, Doering BK. Prevalence of prolonged grief disorder. J Affect Disord. 2021 May;287:301–7.

28. Shevlin M, Redican E, Hyland P, Murphy J, Karatzias T, McBride O, Bennett K, Butter S, Hartman TK, Vallières F, Bentall RP. Symptoms and levels of ICD-11 Prolonged Grief Disorder in a representative community sample of UK adults. Soc Psychiatry Psychiatr Epidemiol. 2023 Oct;58(10):1535–47.
29. Siddiqua A, Ahmad S, Nawaz I, Zeeshan M, Rao A. The smoldering pandemic: Prevalence of prolonged grief disorder. A cross-sectional study of bereaving adults during the COVID pandemic in Pakistan. Asian J Psychiatry. 2024 Jan;91:103870.
30. Singer J, McLean E, Kahler J, Papa A. An evaluation of common risk factors for prolonged grief disorder using the international classification of diseases-11 criteria. Aging Ment Health. 2022 Nov 2;26(11):2202–7.
31. Specht F, Vöhringer M, Knaevelsrud C, Wagner B, Stammel N, Böttche M. Prolonged grief disorder in Arabic-speaking treatment-seeking populations: Relationship with socio-demographic aspects, loss- and trauma-related characteristics, and mental health support. Front Psychiatry. 2022 Sep 15;13:933848.
32. Tay AK, Mohsin M, Rees S, Tam N, Kareth M, Silove D. The structure and psychosocial correlates of complicated bereavement amongst refugees from West Papua. Soc Psychiatry Psychiatr Epidemiol. 2019 Jun;54(6):771–80.
33. Treml J, Linde K, Brähler E, Kersting A. Prolonged grief disorder in ICD-11 and DSM-5-TR: differences in prevalence and diagnostic criteria. Front Psychiatry. 2024 Feb 8;15:1266132.
34. Zhang H, Shang Z, Wu L, Sun Z, Zhang F, Sun L, Zhou Y, Wang Y, Liu W. Prolonged grief disorder in Chinese Shidu parents who have lost their only child. Eur J Psychotraumatology. 2020 Dec 31;11(1):1726071.
35. Zhou N, Wen J, Stelzer EM, Killikelly C, Yu W, Xu X, Shi G, Luo H, Wang J, Maercker A. Prevalence and associated factors of prolonged grief disorder in Chinese parents bereaved by losing their only child. Psychiatry Res. 2020 Feb;284:112766.
